# Inhibition of sympathetic tone via hypothalamic descending pathway propagates glucocorticoid-induced endothelial impairment and osteonecrosis of the femoral head

**DOI:** 10.1038/s41413-024-00371-3

**Published:** 2024-11-08

**Authors:** Wenkai Shao, Bo Wang, Ping Wang, Shuo Zhang, Song Gong, Xiaodong Guo, Deyu Duan, Zengwu Shao, Weijian Liu, Lei He, Fei Gao, Xiao Lv, Yong Feng

**Affiliations:** 1 Department of Orthopedics, Union Hospital, Tongji Medical College, Huazhong University of Science and Technology, Wuhan, 430022 China; 2 Department of Rehabilitation, Wuhan No.1 Hospital, Tongji Medical College, Huazhong University of Science and Technology, Wuhan, 430022 China

**Keywords:** Pathogenesis, Metabolic bone disease

## Abstract

Osteonecrosis of the femoral head (ONFH) is a common complication of glucocorticoid (GC) therapy. Recent advances demonstrate that sympathetic nerves regulate bone homeostasis, and GCs lower the sympathetic tone. Here, we show that the dramatically decreased sympathetic tone is closely associated with the pathogenesis of GC-induced ONFH. GCs activate the glucocorticoid receptor (GR) but hinder the activation of the mineralocorticoid receptor (MR) on neurons in the hypothalamic paraventricular nucleus (PVN). This disrupts the balance of corticosteroid receptors (GR/MR) and subsequently reduces the sympathetic outflow in the PVN. Vascular endothelial cells rapidly react to inhibition of sympathetic tone by provoking endothelial apoptosis in adult male mice treated with methylprednisolone (MPS) daily for 3 days, and we find substantially reduced H-type vessels in the femoral heads of MPS-treated ONFH mice. Importantly, treatment with a GR inhibitor (RU486) in the PVN promotes the activation of MR and rebalances the ratio of GR and MR, thus effectively boosting sympathetic outflow, as shown by an increase in tyrosine hydroxylase expression in both the PVN and the sympathetic postganglionic neurons and an increase in norepinephrine levels in both the serum and bone marrow of the femoral head of MPS-treated mice. Rebalancing the corticosteroid receptors mitigates GC-induced endothelial impairment and ONFH and promotes angiogenesis coupled with osteogenesis in the femoral head, while these effects are abolished by chemical sympathectomy with 6-OHDA or adrenergic receptor-β2 (Adrb2) knockout. Furthermore, activating Adrb2 signaling in vivo is sufficient to rescue the GC-induced ONFH phenotype. Mechanistically, norepinephrine increases the expression of the key glycolytic gene 6-phosphofructo-2-kinase/fructose-2,6-bisphosphatase 3 (PFKFB3) via Adrb2-cyclic AMP response element-binding protein (CREB) signaling. Endothelial-specific overexpression of PFKFB3 attenuates endothelial impairment and prevents severe osteonecrosis in MPS-treated Adrb2 knockout mice. Thus, GC inhibits sympathetic tone via the hypothalamic descending pathway, which, in turn, acts as a mediator of GC-induced ONFH.

## Introduction

Osteonecrosis of the femoral head (ONFH) is a serious ischemic bone illness with a high disability rate that often occurs among patients experiencing long-term and/or high-dose GC therapy.^1^ Knowledge gaps continue to restrict clinical care despite the identification of numerous pathophysiological mechanisms in relation to GC-induced ONFH. The femoral head is highly dependent on the vasculature, which serves a critical function in maintaining bone homeostasis by actively regulating bone formation and supplying nutrients to bone cells.^2^ Patients with ONFH have a strong association with major adverse cardiovascular events compared with healthy subjects.^3^ Generally, GCs trigger early apoptosis to endothelial cells (ECs) and disrupt skeletal angiogenesis.^4,5^ Injured ECs drive coagulation abnormalities and promote hypofibrinolysis. Subsequent hemodynamic changes and reduced vasodilation capability trigger inadequate blood supply and oxygen deprivation.^6^ These aberrant alternations, together with the accumulative adverse effects of GCs, cause extensive ischemic cell death, which ultimately leads to structural deterioration or even femoral head collapse.^7^ Numerous studies have noted that vessel injury is an initiating factor for ONFH pathogenesis.^8–10^ However, the underlying mechanism of the vasculature impairment in ONFH remains elusive.

Interoception exerts numerous physiological responses by triggering postganglionic sympathetic nerves inside target organs and tissues, like the skin, lung, kidney, liver, and bone, thereby enabling the body to protect itself against external threats and drug administration.^11^ The sympathetic nervous system (SNS) participates in inflammation, immunity, and bone metabolism.^12–14^ SNS overactivation usually inhibits bone formation and promotes bone resorption,^15^ whereas sympathetic denervation displayed low bone mass and delayed bone fracture healing.^16,17^ The sympathetic tone was attenuated in patients treated with GC.^18,19^ Beckmann et al. first noted the loss of sympathetic nerves in the femoral head of GC-induced ONFH.^20^ However, the underlying mechanism responsible for the SNS in GC-induced osteonecrosis remains unclear. The paraventricular nucleus (PVN) of the hypothalamus regulates sympathetic outflow by integrating and coordinating the release of neurotransmitters in peripheral sympathetic postganglionic neurons among organ centers.^11^ The continuous activation of the mineralocorticoid receptor (MR) has been reported to promote the survival of neurons and sympathetic outflow in the PVN.^21^ Nevertheless, excessive or prolonged activation of glucocorticoid receptors (GRs) by synthetic GCs induces neuronal apoptosis and disrupts the normal function of neurons, subsequently causing cognitive, mood, and stress disorders.^22^ A recent advance indicates that rebalancing GR and MR activation in the PVN alleviated neuronal injury after administration of a GR agonist.^23^ Our previous studies showed that altering sympathetic tone could help maintain bone homeostasis and bone-fat balance via the central nervous system (CNS).^24,25^ Therefore, it is reasonable to speculate that dysfunction of SNS participate in GCs induced ONFH.

H-type or CD31^hi^Endomucin (EMCN)^hi^ vessels are a critical constituent within the microenvironment of the metabolically specialized bone that couple angiogenesis and bone formation. Our previous study demonstrated that GCs lead to vascular senescence and a decrease in H-type vessels through inhibition of osteoclast angiogenesis coupling factor.^26^ The endothelium relies largely on glycolysis to complete angiogenesis.^27,28^ Anomalous glycolysis has been documented as a crucial pathological process in illnesses related with EC.^29^ Glycolysis is mainly stimulated by the regulatory enzyme 6-phosphofructo-2-kinase/fructose-2,6-bisphosphatase 3 (PFKFB3), and blockade or genetic deletion of endothelial PFKFB3 could attenuate EC-associated diseases.^30,31^ An interesting observation is that vascular network is closely associated with sympathetic nerves that have a perivascular distribution on bone.^32^ Persistent activation of SNS has been demonstrated to increase vascular density and promote proteins secretion involved in angiogenesis during wound healing and tumor growth.^33,34^ Zahalka et al. showed that sympathetic nerves activate an angio-metabolic switch in aberrant cancer that is highly reliant on angiogenesis.^35^ Miranda et al. further demonstrated that glycolytic metabolism was promoted by the SNS-mediated adrenergic receptor-β2 (Adrb2) signaling pathway.^36^ Given the effect of the SNS on endothelial glycolysis, we hypothesized that sympathetic nerves may regulate vascular homeostasis in the femoral head.

Here, we explored regulating the sympathetic descending pathway in the PVN protects the femoral head against GC-induced osteonecrosis. Sympathetic denervation in the femoral head further aggravated GC-induced osteonecrosis. Mechanistically, elevated sympathetic tone stimulated PFKFB3 expression and endothelial glycolysis, resulting in an increase in H-type vessels in the femoral head, whereas deletion of Adrb2 or CREB inhibition attenuated these effects. Finally, overexpression of PFKFB3 rescued GC-induced osteonecrosis in an Adrb2 knockout mice. Our results shed light on the underlying mechanism the correlation of sympathetic tone in the hypothalamic PVN and the angiogenesis of H-type vessel in the femoral head, which offers a novel therapeutic strategy for GC-induced ONFH.

## Results

### Sympathetic nervous system dysfunction occurred in GC-induced ONFH

**Fig. 1:**
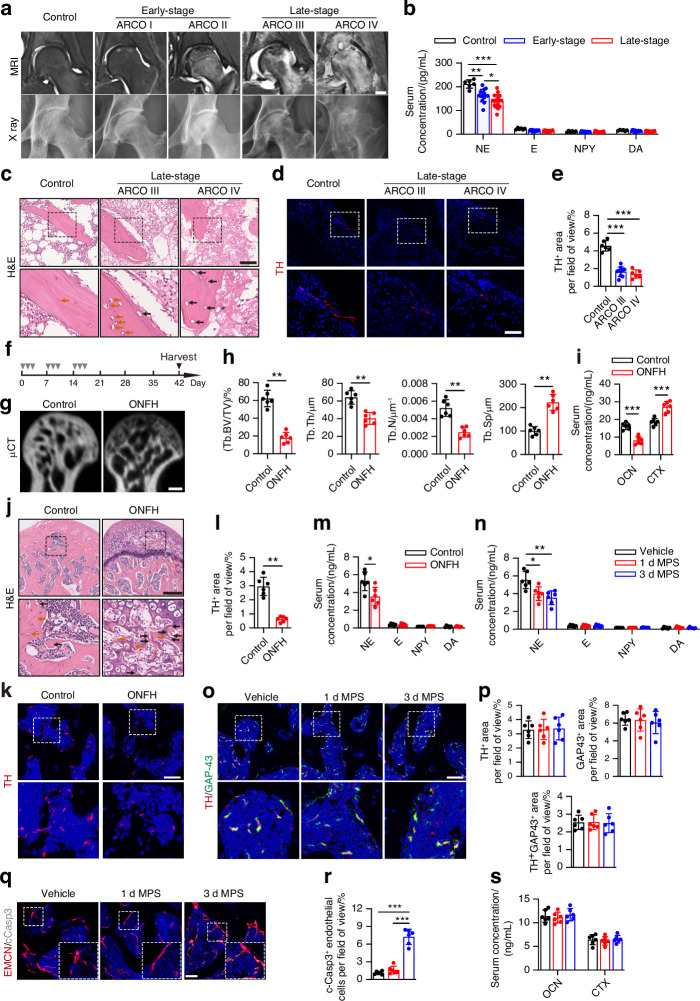
Sympathetic nervous system dysfunction occurred in GC-induced ONFH. **a** Representative human femoral head MRI and X-ray images for the healthy control and different ARCO stages ranging from ARCO I to ARCO IV (early-stage: ARCO I and ARCO II, late-stage: ARCO III and ARCO IV). Scale bar: 2 cm. **b** Quantitative analysis of ELISA assay for NE, E, NPY, and DA levels in human serum from the healthy control and GC-induced ONFH patients under early- and late-stage. **c** Representative H&E staining images of human femoral heads tissues from the healthy control and late-stage patients. Scale bar: 100 μm. Brown arrows indicate dying osteocytes, black arrows empty osteocytic lacunae. **d**, **e** Representative images of immunofluorescence of TH^+^ sympathetic nerves (red) and quantitative analysis for human femoral heads from the healthy control and late-stage patients. Scale bar: 200 μm. **f** Experimental design graph for GC-induced ONFH model establishment. 12-week-old mice were treated with methylprednisolone (MPS) at 20 mg/kg or vehicle on the first 3 days of a week for 3 weeks, and then were harvested 6 weeks after the first injection. One down arrow (grey) represented one time injection. **g**, **h** μCT reconstruction images of femoral heads and quantification of Tb. BV/TV, Tb.Th, Tb. N and Tb. Sp. Scale bar: 1 mm. **i** Quantitative analysis of ELISA assay for OCN and CTX levels in serum. **j** Representative H&E staining images of femoral heads tissues from healthy control mice and GC-induced ONFH mice. Scale bar: 200 μm. Brown arrows indicate dying osteocytes, black arrows empty osteocytic lacunae. **k**, **l** Representative images of immunofluorescence of TH^+^ sympathetic nerves (green) and quantitative analysis for femoral heads from healthy control mice and GC-induced ONFH mice. Scale bar: 100 μm. **m** Quantitative analysis of ELISA assay for NE, E, NPY, and DA levels in serum from healthy control mice and GC-induced ONFH mice. **n** − **s** 12-week-old mice were treated with MPS at 20 mg/kg or vehicle daily for different time periods as indicated. **o**, **p** Representative immunofluorescence co-staining of TH (red) and GAP43 (green) and quantitative analysis for TH^+^ sympathetic nerves and GAP43^+^ nerves in femoral heads. Scale bar: 100 μm. **q**, **r** Representative immunofluorescence co-staining of EMCN (red) and c-Caspase-3 (white) and quantitative analysis for vessels expressing both EMCN and c-Caspase-3 in femoral heads. Scale bar: 50 μm. Quantitative analysis of ELISA assay for (**n**) NE, E, NPY, DA, (**s**) OCN, and CTX levels in serum. All data were presented as means ± SD, *n* ≥ 6 per group; *P < 0.05. **P < 0.01. ***P < 0.001. Statistical significance was determined by two-tailed Student’s *t*-test (**h**, **i**, **l**, **m**). Statistical significance was determined by one-way ANOVA with Bonferroni post hoc test (**b**, **e**, **n**, **p**, **r**, **s**)

To explore how the SNS changes in GC-induced ONFH, we collected clinical serum and femoral head specimens in accordance with Association Research Circulation Osseous (ARCO) guidelines. Clinical imaging data revealed that MRI was more sensitive than X-rays in identifying early lesions prior to collapse in the femoral head, and bone destruction gradually worsened as the ARCO stage advanced (Fig. 1a). The levels of NE were significantly reduced in patients’ serum with GC-induced ONFH in comparison to the controls, and NE levels in the late stage were relative lower to the early stage; however, the levels of other sympathetic neurotransmitters were unchanged, as shown by enzyme-linked immunosorbent assay (ELISA) (Fig. 1b). Hematoxylin and eosin (H&E) staining images revealed that marrow structures were filled with a larger amount of fat vacuoles, granular eosinophilic material, and degenerative cells with pyknotic nuclei and apparent cytoplasm in the femoral heads of the patients with ONFH (Fig. 1c); and an increasing number of dying osteocytes and empty osteocytic lacunae (a hallmark of osteonecrosis) were observed in the trabecular bones of the femoral heads of the patients with ONFH (Fig. 1c). These necrotic changes were more pronounced in patients at ARCO stage IV than in patients at ARCO stage III (Fig. 1c). Immunostaining revealed a significantly decrease in the number of TH^+^ sympathetic nerves in the femoral heads of the patients with ONFH at both ARCO stages III and IV relative to the control samples, among whom the TH^+^ sympathetic nerve density did not correlate with the ARCO stage (Fig. 1d, e). We then treated 12-week-old male C57BL/6 mice with methylprednisolone (MPS) to establish the GC-induced ONFH model, in which mice received MPS or vehicle on the first 3 days of each week for 3 weeks (Fig. 1f). Microcomputed tomography (μCT)-reconstructed images of femoral heads demonstrated that more substantial necrotic lesions with low density and notable bone loss emerged in the femoral heads of MPS-treated ONFH mice relative to those of vehicle-treated healthy mice (Fig. 1g). MPS treatment significantly decreased the trabecular bone volume (Tb. BV/TV), trabecular thickness (Tb. Th), and trabecular number (Tb. N), while obviously increasing the trabecular separation (Tb. Sp) compared to that of vehicle-treated mice (Fig. 1h). The serum levels of osteocalcin, a marker of osteoblastic bone formation, were significantly decreased, and the levels of osteoclast bone resorption marker carboxyterminal collagen crosslinks (CTX) were significantly increased in the ONFH mice relative to the control (Fig. 1i), suggesting an increased bone catabolism. H&E staining showed MPS treatment resulted in a higher number of dying osteocytes and empty osteocytic lacunae in the trabecular bone, as well as more fat vacuoles and anomalous cells with pyknotic nuclei and cytoplasmic voids in the marrow regions of the femoral heads, which were rarely identified in the vehicle-treated mice (Fig. 1j). We also found a lower number of TH^+^ sympathetic nerves in the femoral head and lower serum NE levels among the sympathetic neurotransmitters in mice after MPS treatment (Fig. 1k–m), suggesting that SNS dysfunction may be closely related to ONFH pathogenesis.

Given that GC treatment rapidly suppressed the sympathetic tone,^18,19^ mice were treated with MPS daily for 1 or 3 days to investigate the impact of GC treatment on femoral head homeostasis in the early stages of osteonecrosis. ELISA showed that only serum NE levels progressively decreased among the sympathetic neurotransmitters in the MPS-treated mice compared to the vehicle-treated mice (Fig. 1n). Growth-associated protein 43 (GAP-43) participates in the regulation of axon regeneration, synaptic function maintenance, and neurotransmitter release.^37,38^ However, there were no notable variations in GAP-43^+^ sympathetic nerve abundance in the femoral heads of vehicle- and MPS-treated mice, as shown with co-immunostaining of GAP-43 and TH (Fig. 1o, p). These findings indicate that a decrease in serum NE levels may not be linked with the function of local sympathetic nerve fibers in the femoral head of mice receiving MPS treatment daily for 1 and 3 days. Notably, we found that almost all apoptotic cells were EMCN^+^ vascular ECs in the mice treated with MPS daily for 3 days but not for 1 day, as shown by co-immunostaining for EMCN and c-Caspase3 in the femoral heads (Fig. 1q, r), whereas MPS treatment did not induce EC apoptosis in the distal femur, liver or kidney (Fig. S1a‒d). The bone remodeling process was unaffected during these periods of MPS treatment relative to that of the vehicle-treated mice, as revealed by ELISA for the serum levels of OCN and CTX (Fig. 1s). Therefore, suppressive sympathetic tone occurred earlier than vessel injury following GC treatment, and ECs were the initial cell type to display an impaired phenotype in the femoral head microenvironment relative to other tissues.

### GCs undermine hypothalamic sympathetic outflow by disrupting the balance of the nuclear GR and MR

**Fig. 2:**
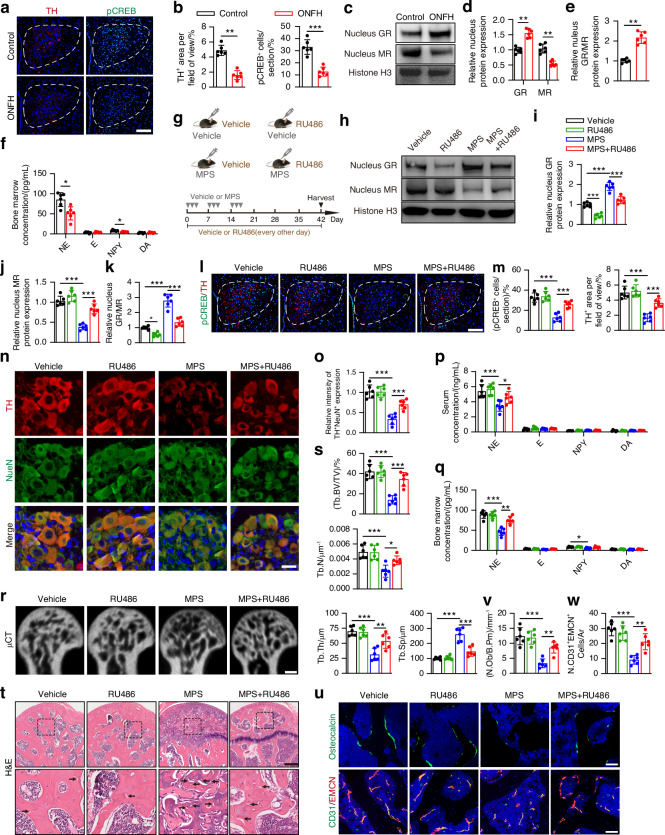
Rebalance of GR and MR activation stimulates sympathetic outflow and prevent ONFH in GC-treated mice. **a**, **b** Representative images of immunofluorescence staining and quantitative analysis of the TH (red) and pCREB (green) in the hypothalamic PVN from healthy control mice and ONFH mice. 12-week-old mice were treated with MPS at 20 mg/kg or vehicle on the first 3 days of a week for 3 weeks, and then were harvested 6 weeks after the first injection. Scale bar: 100 μm. **c**−**e** Representative images of WB and quantitative analysis of nucleus GR and MR expression in the PVN of the hypothalamus from vehicle-treated healthy mice and GC-induced ONFH mice. **f** Quantitative analysis of ELISA assay for NE, E, NPY, DA levels in bone marrow of the femoral heads. **g** Experimental design graph for exploring the effects of GR inhibitor (RU486) treatment in PVN on the femoral heads of vehicle- or MPS-treated mice. Mice were harvested 6 weeks after the first injection of MPS. One down arrow (grey) represented one time treatment with MPS or vehicle. **g**−**w** Mice were grouped as vehicle- and MPS-treated mice and their littermates with vehicle or RU486 treatment in PVN. **h**−**k** Representative images of WB and quantitative analysis of nucleus GR and MR expression in the hypothalamic PVN. **l, m** Representative immunofluorescence co-staining of TH (red) with pCREB (green) and quantitative analysis of density of pCREB^+^ and TH^+^ cells in the hypothalamic PVN. Scale bar: 100 μm. **n**, **o** Representative images of immunofluorescence co-staining of TH (red) and NeuN (green) and quantitative analysis of TH^+^ NeuN^+^ postganglionic neurons in paravertebral chain ganglia (L2-L4) **p**, **q** Quantitative analysis of ELISA assay for NE levels in serum and bone marrow of the femoral heads. **r**, **s** μCT reconstruction images and quantitative analysis of Tb. BV/TV, Tb. Th, Tb. N and Tb. Sp of femoral heads. Scale bar: 1 mm. **t** H&E staining images of femoral heads. Scale bar: 200 μm. Black arrows indicate empty osteocytic lacunae. **u** Representative images of immunofluorescence staining of OCN (green) and co-staining of CD31 (green) with EMCN (red) (**v, w**) and quantitative analysis of the number of OCN^+^ osteoblasts and CD31^+^EMCN^+^ cells in the femoral heads. Scale bar: 50 μm. All data were presented as means ± SD, *n* = 6 per group; *P < 0.05. **P < 0.01. ***P < 0.001. Statistical significance was determined by one-way ANOVA with Bonferroni post hoc test (**i**−**k, m, o**−**q, s, v, w**). Statistical significance was determined by two-tailed Student’s *t*-test (**b, d**−**f**)

The hypothalamic paraventricular nucleus is crucial for sympathetic outflow.^11^ CREB phosphorylation in PVN neurons directly stimulates TH activation (a sign of sympathetic outflow in the CNS).^39^ Our results demonstrated that sympathetic tone, as well as CREB phosphorylation, were significantly repressed in the PVN of the hypothalamus of the MPS-treated ONFH mice relative to the vehicle-treated healthy mice (Fig. 2a, b). We then investigated the nuclear protein levels of MR and GR in the hypothalamic PVN of the MPS-treated mice with ONFH. MPS treatment notably increased the nuclear GR expression but decreased the nuclear MR expression in the PVN of the hypothalamus compared to that of mice given vehicle treatment, indicating that MPS treatment in mice disrupted the balance of nuclear GR and MR activation in the PVN (Fig. 2c–e). We also observed that daily treatment with MPS for 1 and 3 days caused decreased levels of nuclear MR, TH expression, and CREB phosphorylation and increased levels of nuclear GR expression in the PVN of the MPS-treated mice relative to the vehicle-treated mice (Fig. S2a–e).

Considering that hypothalamic PVN stimulates catecholamine release from the adrenal medulla by coordinating the hypothalamus-pituitary-adrenal (HPA) axis, we then examined whether GC treatment interfered with the function of the HPA axis in MPS-treated ONFH mice. Decreased *Crh* mRNA levels in the PVN and corticosterone levels in the serum were observed in MPS-treated ONFH mice relative to vehicle-treated healthy mice and in mice treated with MPS daily for 1 and 3 days relative to mice treated with vehicle (Fig. S3a, b), suggesting that repression of the HPA axis occurred in both the early-stages and late-stages of MPS-treated ONFH mice. Intriguingly, ELISA showed significantly decreased NE levels among the sympathetic neurotransmitters in the bone marrow of the femoral heads of the MPS-treated ONFH mice relative to those of the vehicle-treated mice, whereas no obvious changes were observed in the levels of NE in the adrenal medulla of mice in response to MPS treatment (Fig. 2f and Fig. S3c). Sympathetic tone regulation of bone homeostasis begins when leptin, a hormone that is mainly secreted by adipocytes, which stimulates sympathetic outflow and neurotransmitter secretion.^40^ Although fat accumulation at the necrotic regions of the femoral head is also related to GC-induced osteonecrosis,^7^ serum leptin levels showed no significant change in the MPS-treated mice compared to the vehicle-treated mice, as demonstrated by ELISA (Fig. S3d). These findings indicate that the decreased NE levels in the femoral head could be derived from GC inhibition of the sympathetic tone in the CNS.

To determine whether restoring the balance of nuclear GR and MR in the PVN regulates CREB-mediated sympathetic outflow, we injected a GR inhibitor (RU486) into the PVN of the MPS-treated mice every other day for 6 weeks to decrease GR overactivation (Fig. 2g). Injection sites were identified by Evans blue staining assays in the PVN of the hypothalamus (Fig. S4a). Western blotting revealed that RU486 treatment significantly decreased GR activation while increasing nuclear translocation of MR in the PVN of the hypothalamus of MPS-treated mice (Fig. 2h–j), suggesting that GR inhibition in the PVN of the MPS-treated mice was sufficient to restore the balance of nuclear GR and MR (Fig. 2k). Consistently, GR inhibition in the PVN resulted in much higher levels of CREB phosphorylation and TH expression in the MPS-treated mice (Fig. 2l, m). The sympathetic projection pathway from the PVN was linked to the femoral head via postganglionic neurons in paravertebral chain ganglia (lumbar levels).^41^ Co-immunostaining for TH and NeuN showed a significant increase of TH expression in sympathetic postganglionic neurons of paravertebral chain ganglia in the PVN in MPS-treated ONFH mice following RU486 treatment (Fig. 2n, o). Restoration of the sympathetic tone was verified by ELISA for neurotransmitter levels in the MPS-treated mice in response to RU486 treatment in the PVN; RU486 treatment significantly increased NE levels in the serum and bone marrow of the femoral heads among the sympathetic neurotransmitters in MPS-treated mice (Fig. 2p, q). We also observed that RU486 treatment in the PVN partially restored the balance of nuclear GR and MR, significantly promoted CREB-mediated sympathetic outflow, and notably increased NE levels in the serum and bone marrow of the femoral head in mice treated with MPS daily for 3 days (Fig. S2f–l). Intriguingly, co-immunostaining for EMCN and c-Caspase-3 showed that RU486 treatment in mice significantly alleviated endothelial apoptosis in response to MPS treatment daily for 3 days, as shown by the reduction in c-Caspase3^+^ endothelial cells in the femoral heads of MPS-treated mice (Fig. S2m, n). These findings suggest that inhibiting the excessive activation of GR in the PVN of the hypothalamus rebalances nuclear GR and MR, thus stimulating sympathetic outflow and increasing the sympathetic tone of the femoral heads in response to GCs.

### Stimulating sympathetic outflow alleviates GC-induced ONFH

To determine whether the restoration of sympathetic tone by maintaining the balance of MR and GR in the PVN could alleviate GC-induced ONFH, we next investigated the changes in mice by histology and imaging. As expected, μCT-reconstructed images of the femoral heads and trabecular bone microarchitectural parameters confirmed that MPS-induced bone deterioration was markedly attenuated by rebalancing GR and MR activation following RU486 treatment in the PVN, as indicated by the much higher levels of Tb. BV/TV, Tb. Th, and Tb. N, as well as the lower levels of Tb. Sp relative to those of the MPS-treated mice (Fig. 2r, s). H&E staining revealed that RU486 injection in the hypothalamic PVN significantly attenuated trabecular bone destruction with a lower number of empty osteocytic lacunae and reduced bone marrow damage with fewer fat vacuoles or degenerating cells in the femoral heads relative to those of the MPS-treated mice (Fig. 2t). Nevertheless, inhibiting only nuclear GR did not cause any notably unfavorable microarchitectural changes in the RU486-treated mice compared to the vehicle-treated mice, as shown by H&E staining and μCT analysis of the trabecular bone parameters (Fig. 2r–t). Immunostaining for OCN demonstrated that MPS treatment led to a considerable reduction in OCN^+^ mature osteoblasts in the femoral heads compared to those of the vehicle-treated mice, whereas restraining GR overactivation in the PVN significantly reversed the decrease in osteoblasts in response to MPS treatment (Fig. 2u, v). However, RU486 treatment in the PVN resulted in fewer osteoclasts and decreased bone resorptive activity in the MPS-treated mice, as demonstrated by TRAP staining and ELISA for serum CTX (Fig. S4b–d). Intriguingly, a specific vessel, named the H-type (CD31^hi^EMCN^hi^) vessel, which spatiotemporally orchestrates the coupling of angiogenesis and osteogenesis, was markedly reduced in MPS-treated mice with ONFH relative to vehicle-treated control mice, as shown by co-immunostaining for CD31 and EMCN (Fig. 2u, w). Interestingly, H-type vessels were present in larger amounts at the femoral heads compared to the tibia, sternum, distal femur, vertebra, or calvarium in adult wild-type mice (Fig. S5). We also found that the increased sympathetic tone significantly decreased the loss of CD31- and EMCN-double-positive ECs in the MPS-treated mice after RU486 injection in the PVN (Fig. 2u, w). Taken together, the above results suggest that maintaining the balance of nuclear GR and MR in the PVN to stimulate sympathetic outflow protects against GC-induced ONFH by improving trabecular bone microarchitecture and promoting H-type vessel angiogenesis coupled with osteogenesis in the femoral head.

Next, we investigated the effects of stimulating sympathetic outflow by RU486 treatment in the PVN on the trabecular bone microarchitecture in the femoral heads of mice in which GC-induced ONFH and sympathetic denervation had already been initiated (Fig. S6a). We found that RU486 treatment in the PVN increased the serum levels of NE in the MPS-treated mice, whereas NE levels in the bone marrow of the femoral heads showed a slightly increase (Fig. S6b). μCT analysis revealed that the microarchitectural bone deterioration that was previously triggered by MPS was not reversed in the mice receiving RU486 treatment (Fig. S6c, d), indicating that stimulating sympathetic outflow in progressing ONFH mice did not have a protective effect on the femoral head microarchitecture in response to GCs.

### Denervation of sympathetic nerves aggravates GC-induced ONFH

**Fig. 3:**
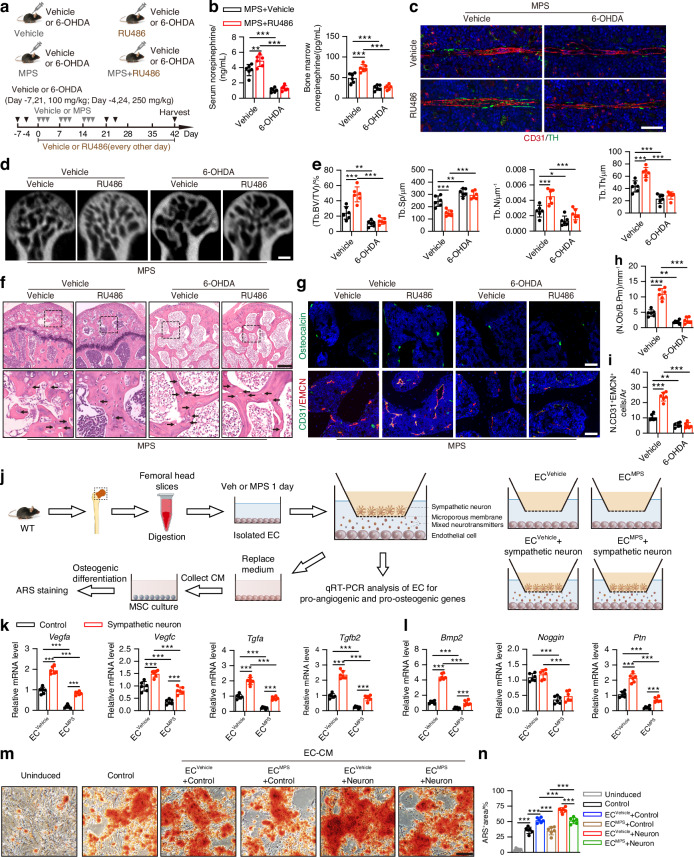
Sympathetic denervation in femoral head blocks sympathetic outflow and aggravates GC-induced ONFH mice. **a** Experimental design graph for examining the effects of 6-OHDA-induced sympathetic denervation on the femoral heads of MPS-treated mice undergoing vehicle or RU486 treatment in PVN. Mice were harvested 6 weeks after the first injection of MPS. One down arrow (grey) represented one time treatment with MPS or vehicle, one down arrow (black) represented one time treatment with 6-OHDA or vehicle. **a**−**i** MPS-treated mice were grouped as vehicle- and 6-OHDA-treated mice and their littermates with vehicle or RU486 treatment in PVN. **b** Quantitative analysis of ELISA assay for NE levels in serum and bone marrow of the femoral heads. **c** Representative immunofluorescence images of TH^+^ sympathetic nerves and CD31^+^ vessels in the bone marrow of mice. Scale bar: 50 μm. **d**, **e** μCT reconstruction images and quantitative analysis of Tb. BV/TV, Tb. Th, Tb. N and Tb. Sp of femoral heads. Scale bar: 1 mm. **f** H&E staining images of femoral heads. Scale bar: 200 μm. Black arrows indicate empty osteocytic lacunae. **g** Representative images of immunofluorescence staining of OCN (green) and co-staining of CD31 (green) with EMCN (red) (**h, i**) and quantitative analysis of the number of OCN^+^ osteoblasts and CD31^+^EMCN^+^ cells in the femoral heads. Scale bar: 50 μm. **j**−**n** ECs were isolated from the femoral heads of 12-week-old wild-type mice. Schematic diagram showing the in vitro co-culture system of sympathetic neurons and ECs following vehicle or MPS treatment for 1 day. Then quantitative RT-PCR analysis of ECs was performed, and conditioned medium (CM) from co-cultured ECs was collected to treat BMSCs during osteogenic differentiation. **k, l** Quantitative RT-PCR analysis of ECs for pro-angiogenic (*Vegfa, Vegfc, Tgfa, and Tgfb2*) and pro-osteogenic (*Bmp2, Noggin, and Ptn*) genes expression. **m**, **n** Representative images of ARS staining and quantification of the positively stained areas in BMSCs after EC-CM treatment. Scale bar: 50 μm. All data were presented as means ± SD, *n* = 6 per group; *P < 0.05. **P < 0.01. ***P < 0.001. Statistical significance was determined by two-way ANOVA with Bonferroni post hoc test

Sympathetic denervation in the femoral head might accompany GC-induced ONFH, or alternatively, it may contribute to the development of osteonecrosis. To further elucidate the role of sympathetic nerves in regulating GC-induced ONFH, we established a 6-OHDA-induced sympathetic denervation mouse model before MPS treatment in which 6-OHDA did not cross the blood brain barrier (Fig. 3a). Immunofluorescence of TH demonstrated that sympathetic nerves were effectively ablated in the femoral heads of the mice following 6-OHDA treatment (Fig. S7a), and NE levels in the serum and femoral head were both notably decreased in 6-OHDA-treated mice relative to vehicle-treated mice (Fig. S7b). MPS treatment further decreased NE levels in the serum and bone marrow of the 6-OHDA-treated mice (Fig. 3b and Fig. S7b). Sympathetic denervation partially abolished the increase in NE levels in the MPS-treated mice in response to RU486 treatment in the PVN (Fig. 3b). Immunofluorescence showed fewer TH^+^ sympathetic nerves in the bone marrow of MPS-treated mice after 6-OHDA treatment (Fig. 3c). Intriguingly, μCT-reconstructed images of the femoral heads revealed that MPS-treated mice with sympathetic denervation induced much more bone destruction and bone loss, as demonstrated by a lower microstructure parameter in Tb. BV/TV, Tb. Th, and Tb. N but a higher Tb. Sp in the trabecular bone (Fig. 3d, e). Unexpectedly, RU486 treatment in the PVN did not reverse the destructive effects on trabecular bone microarchitecture in the femoral heads of MPS-treated mice following 6-OHDA treatment (Fig. 3d, e). These results indicate that the integrity of the local sympathetic nerves in the femoral heads is required for the hypothalamic descending pathway to coordinate sympathetic outflow and orchestrate trabecular bone microarchitecture. H&E staining showed that sympathetic denervation caused much larger areas of aberrant bone marrow and microarchitecture filled with more anomalous cells, granular eosinophilic material, and fat vacuoles in the femoral heads of the MPS-treated mice (Fig. 3f). These mice had a greater amount of empty osteocytic lacunae in trabecular bone following sympathetic denervation relative to the mice with MPS treatment only (Fig. 3f). Sympathetic denervation also abrogated the protective effects of stimulating sympathetic outflow on the femoral heads of the MPS-treated mice (Fig. 3f). Furthermore, sympathetic denervation resulted in fewer osteoblasts and H-type vessels in the femoral heads of the MPS-treated mice, as shown by immunostaining for OCN and co-immunostaining for CD31 and EMCN (Fig. 3g–i). RU486 treatment in the PVN did not reverse the reduction in the number of osteoblasts and H-type vessels in the femoral heads of the mice with sympathetic denervation treated with MPS (Fig. 3g–i). However, 6-OHDA treatment did not induce an osteonecrosis phenotype but did reduce the number of osteoblasts and H-type vessels in the femoral heads of vehicle-treated mice, and these effects were not reversed by RU486 treatment in the PVN (Fig. S7c–g). These findings suggest that sympathetic denervation can abolish the femoral head protection mediated by sympathetic outflow and exacerbate GC-induced ONFH by further disrupting angiogenesis-osteogenesis coupling.

To further examine the effects of sympathetic innervation on angiogenesis-osteogenesis coupling, we isolated ECs from the femoral heads and sympathetic neurons in wild-type (WT) mice and treated ECs with MPS at 100 μmol/L for 24 h prior to coculturing (Fig. 3j), based on the report that the peak concentration of GCs in blood vessels can reach 100 μmol/L throughout high-dose GC administration.^42^ Femoral head ECs were identified by immunostaining for the specific marker von Willebrand factor (vWF), flow cytometry for surface markers (CD31^+^, CD45^−^, and CD106^+^), and the angiogenic ability to form a capillary-like network on Matrigel (Fig. S8a–c). Sympathetic neuron is identified by coimmunostaining for TH and the synaptic marker β-tubulin 3 (Tubb3), which has a slender and polysynaptic shape (Fig. S8d). Quantitative real-time polymerase chain reaction (qRT-PCR) demonstrated that vehicle-treated ECs exhibited upregulated expression of pro-angiogenesis genes (*Vegfa, Vegfc, Tgfa*, and *Tgfb2*) and pro-osteogenesis genes (*Bmp2 and Ptn*) after being cocultured with sympathetic neurons (Fig. 3k, l). MPS treatment markedly decreased the expression of a subset of genes related to pro-angiogenesis (*Vegfa, Vegfc, Tgfa*, and *Tgfb2*) and pro-osteogenesis (*Bmp2, Noggin*, and *Ptn*) relative to vehicle-treated ECs, whereas coculturing ECs with sympathetic neurons significantly increased the expression of these genes, except for *Noggin*, relative to ECs from MPS-treated mice alone (Fig. 3k, l). We then treated mouse bone marrow mesenchymal stem cells (BMSCs) with different conditioned media (CM) collected from vehicle- or MPS-treated ECs after coculture with sympathetic neurons (Fig. 3j). Alizarin Red S (ARS) staining revealed an increase in osteogenesis in BMSCs following conditioned medium treatment from vehicle-treated ECs alone relative to the control, and coculture with sympathetic neurons further promoted the osteogenesis of vehicle-treated EC-CM in BMSCs (Fig. 3m, n). Although MPS-treated EC-CM decreased osteogenesis in BMSCs relative to vehicle-treated EC-CM, CM from MPS-treated ECs cocultured with sympathetic neurons was sufficient to increase osteogenesis in BMSCs (Fig. 3m, n). These findings suggest that sympathetic innervation may offer protection against GC-induced ONFH by increasing the release of pro-angiogenic and pro-osteogenic factors in ECs; thereby, ECs strengthen the angiogenesis-osteogenesis coupling between ECs and BMSCs in an autocrine or paracrine manner.

### Activation of Adrb2 prevents the onset of ONFH with sympathetic denervation

**Fig. 4:**
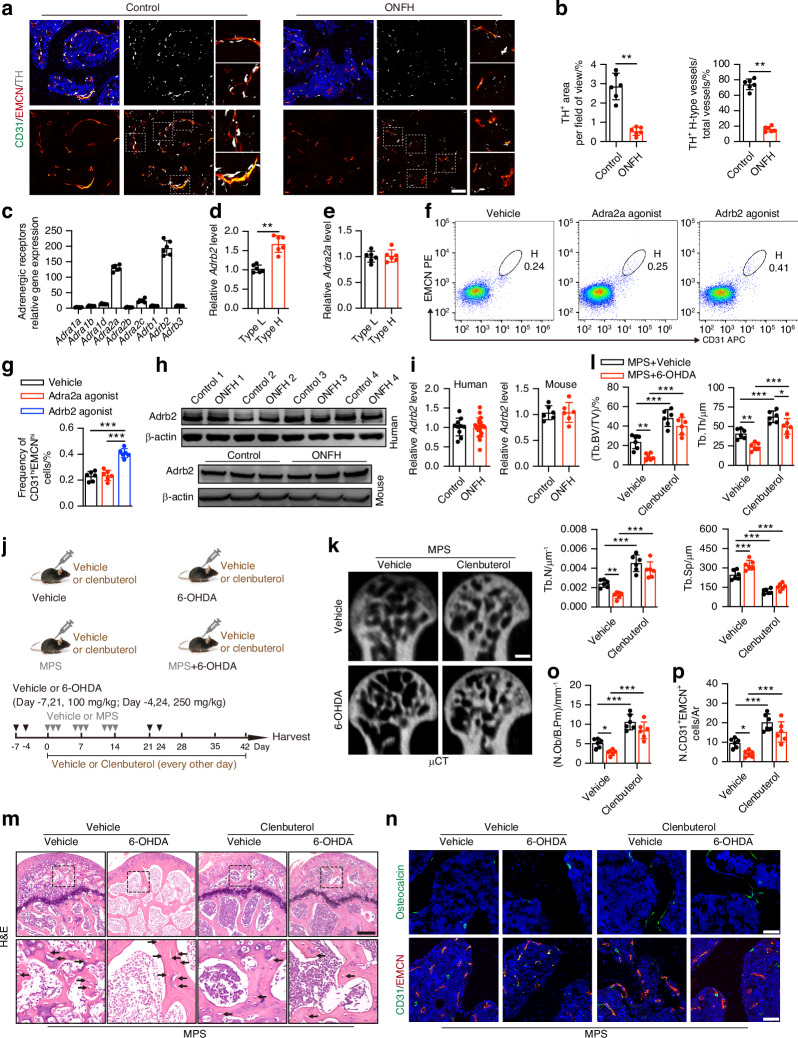
Activation of Adrb2 prevents the onset of osteonecrosis with sympathetic denervation. **a**, **b** Representative immunofluorescence co-staining of CD31 (green), EMCN (red), and TH (white) and quantitative analysis for TH^+^ sympathetic nerves and TH^+^ H-type vessels in the femoral heads from vehicle-treated healthy control mice and MPS-induced ONFH mice. Scale bar: 50 μm. **c** Quantitative RT-PCR analysis of all adrenergic receptors from ECs in the femoral heads of wildtype mice. **d**, **e** Quantitative RT-PCR analysis for *Adrb2* and *Adra2a* expression from H-type and L-type ECs in the femoral heads of wildtype mice. **f**, **g** FACS analysis of the femoral heads single-cell suspensions co-stained with CD31 and EMCN and quantitative analysis of CD31^hi^EMCN^hi^ cells in wildtype mice treated with vehicle, Adra2a agonist (clonidine), or Adrb2 agonist (clenbuterol). **h**, **i** Representative images of WB for Adrb2 expression and quantitative RT-PCR analysis for *Adrb2* gene expression in the femoral heads from humans and mice. **j** Experimental design graph for validating the effects of Adrb2 activation on the femoral heads in MPS-treated mice after 6-OHDA-induced sympathetic denervation. Mice were harvested 6 weeks after the first injection of MPS. One down arrow (grey) represented one time vehicle or MPS treatment, and one down arrow (black) represented one time vehicle or 6-OHDA treatment. **j**−**p** MPS-treated mice were grouped as vehicle and 6-OHDA treatment and their littermates were treated with vehicle or clenbuterol. **k, l** μCT reconstruction images and quantitative analysis of Tb. BV/TV, Tb. Th, Tb. N and Tb. Sp of femoral heads. Scale bar: 1 mm. **m** H&E staining images of femoral heads. Scale bar: 200 μm. Black arrows indicate empty osteocytic lacunae. **n** Representative images of immunofluorescence staining of OCN (green) and co-staining of CD31 (green) with EMCN (red) (**o, p**) and quantitative analysis of the number of OCN^+^ osteoblasts and CD31^+^EMCN^+^ cells in the femoral heads. Scale bar: 50 μm. All data were presented as means ± SD, *n* = 6 per group; *P < 0.05. **P < 0.01. ***P < 0.001. Statistical significance was determined by two-tailed Student’s *t*-test (**b, d, e, i**). Statistical significance was determined by one-way ANOVA with Bonferroni post hoc test (**g**). Statistical significance was determined by two-way ANOVA with Bonferroni post hoc test (**l, o, p**)

Type H vessels, a specific subtype highly expressing CD31 and EMCN, have been shown to facilitate the growth of bone vasculature, couple angiogenesis with osteogenesis, and recruit osteoprogenitors and mesenchymal stem cells (MSCs).^43,44^ In the femoral head, TH^+^ sympathetic nerves were identified as overlapping or adjacent to H-type vessels, suggesting a similar distribution to H-type vessels (Fig. 4a, b), and they were wrapped around the H-type vessels with which they were associated rather than CD31^+^ vessels in the diaphysis;^45^ a notable reduction in TH^+^ sympathetic nerves was observed in the femoral heads of the mice with MPS-induced ONFH relative to the vehicle-treated healthy mice, as evidenced by co-immunostaining for CD31, EMCN, and TH (Fig. 4a, b). To explore how H-type vascular ECs responded to sympathetic outflow, we separated H-type (CD31^hi^EMCN^hi^) and L-type (CD31^lo^EMCN^lo^) vascular ECs in the femoral heads of WT mice by fluorescence-activated cell sorting (FACS) assays (Fig. S9). Among all the adrenergic receptors, *Adrb2* was not only highly expressed in total ECs of the femoral head but also had much higher expression in CD31^hi^EMCN^hi^ ECs relative to CD31^lo^EMCN^lo^ ECs, as shown by qRT**-**PCR (Fig. 4c, d). Although *Adra2a* expression was second only to *Adrb2* expression, there were no significant differences in CD31^hi^EMCN^hi^ and CD31^lo^EMCN^lo^ ECs (Fig. 4c–e). Consistent with these results, flow cytometry analysis showed that the CD31^hi^EMCN^hi^ EC abundance significantly increased in the femoral heads of the 4-week Adrb2 agonist (clenbuterol)-treated mice relative to the vehicle-treated mice, whereas Adra2a agonist (clonidine) treatment for 4 weeks did not affect the number of CD31^hi^EMCN^hi^ ECs (Fig. 4f, g). To measure Adrb2 levels clinically, we found that Adrb2 expression was not significantly different in the femoral heads of late-stage GC-induced ONFH patient tissue relative to normal femoral head tissue, consistent with the comparison of the mice with MPS-induced ONFH to the vehicle-treated mice, as shown by Western blotting and qRT-PCR analysis (Fig. 4h, i). Nevertheless, only ECs, but not osteoblasts, mesenchymal stem cells, and monocytes/macrophages, had elevated *Adrb2* mRNA levels in the femoral heads of mice treated with MPS daily for 3 days compared to those of the vehicle-treated mice (Fig. S10a–d), but in vitro assays showed that MPS at 100 μmol/L did not promote *Adrb2* expression in either ECs or other cells (Fig. S10e–h). These findings suggest that ECs are more likely to respond and increase *Adrb2* levels to compensate for the inhibition of sympathetic outflow in the early stage in MPS-treated mice.

We next used an Adrb2 agonist (clenbuterol) to examine whether mimicking sympathetic outflow without activating the HPA axis alleviates GC-induced ONFH after sympathetic denervation by 6-OHDA (Fig. 4j). Intriguingly, Adrb2 agonist cotreatment every other day for 6 weeks significantly attenuated trabecular bone destruction and bone loss, as shown by μCT reconstruction images and quantitative analysis; MPS-treated mice with Adrb2 activation induced higher Tb. BV/TV, Tb. Th, and Tb. N values, and lower Tb. Sp values in the femoral heads after sympathetic denervation or not (Fig. 4k, l). H&E staining showed that Adrb2 agonist cotreatment notably moderated trabecular bone and bone marrow deterioration, as evidenced by a decrease in empty osteocytic lacunae, anomalous cells, granular eosinophilic material, and fat vacuoles in the femoral heads of MPS-treated mice (Fig. 4m). Furthermore, Adrb2 activation led to higher levels of H-type ECs and osteoblasts in the femoral heads of the MPS-treated mice, regardless of sympathetic denervation, as shown by co-immunostaining for CD31 and EMCN and immunostaining for OCN (Fig. 4n–p). Although sympathetic denervation in MPS-treated mice exhibited a more severe ONFH phenotype, including much larger areas of aberrant bone marrow and microarchitecture filled with more anomalous cells, granular eosinophilic material, and fat vacuoles relative to the mice with MPS treatment only, Adrb2 activation was sufficient to protect the femoral heads from more devastating changes in these mice (Fig. 4m). Of note, the Adrb2 agonist led to lower Tb. BV/TV, Tb. Th, and Tb. N values, and higher Tb. Sp values in the femoral heads of the mice following sympathetic denervation, as evidenced by μCT analysis (Fig. S11a, b). Adrb2 activation significantly increased the number of H-type ECs in the femoral heads of vehicle- and 6-OHDA-treated mice but resulted in a remarkable reduction in OCN^+^ mature osteoblasts in the femoral heads of mice following sympathetic denervation (Fig. S11c–e), suggesting that angiogenesis-osteogenesis coupling appears to occur in mice in response to MPS treatment. Overall, these results indicate that activating Adrb2 can mimic sympathetic outflow in the femoral head close to that seen in a physiological state and thus ameliorate GC-induced ONFH.

### Adrb2 is indispensable in sympathetic nerve-dysregulated ONFH after GC administration

**Fig. 5:**
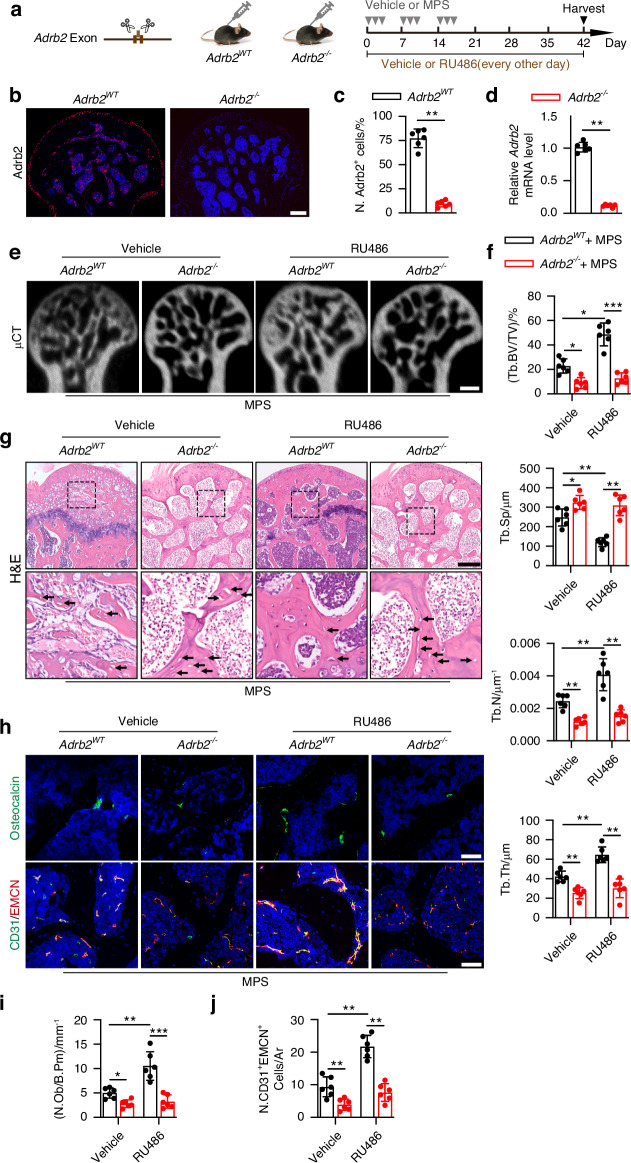
Knockout of Adrb2 abolishes the bone-protective effects of sympathetic outflow and accelerate ONFH in GC-treated mice. **a** Experimental design graph for investigating the effects of Adrb2 deletion on the femoral heads in MPS-treated mice receiving vehicle or RU486 treatment in PVN. Mice were harvested 6 weeks after the first injection of MPS. One down arrow (grey) represented one time treatment with vehicle or MPS. **b, c** Representative images of immunofluorescence staining of Adrb2 (red) and quantitative analysis of Adrb2^+^ cells in the femoral heads from *Adrb2*^*WT*^ *and Adrb2*^*−/−*^ mice. Scale bar: 200 μm. **d** Quantitative RT-PCR analysis for *Adrb2* gene expression from ECs in the femoral heads of *Adrb2*^*WT*^ *and Adrb2*^*−/−*^ mice. **e**−**j** MPS-treated *Adrb2*^*WT*^ *and Adrb2*^*−/−*^ mice were grouped as vehicle or RU486 treatment in PVN. **e**, **f** μCT reconstruction images and quantitative analysis of Tb. BV/TV, Tb. Th, Tb. N and Tb. Sp of femoral heads. Scale bar: 1 mm. **g** H&E staining images of femoral heads. Scale bar: 200 μm. Black arrows indicate empty osteocytic lacunae. **h** Representative images of immunofluorescence staining of OCN (green) and co-staining of CD31 (green) with EMCN (red) (**i**, **j**) and quantitative analysis of the number of OCN^+^ osteoblasts and CD31^+^EMCN^+^ cells in the femoral heads. Scale bar: 50 μm. All data were presented as means ± SD, *n* = 6 per group; *P < 0.05. **P < 0.01. ***P < 0.001. Statistical significance was determined by two-tailed Student’s *t*-test (**c, d**). Statistical significance was determined by two-way ANOVA with Bonferroni post hoc test (**f, i, j**)

To further investigate the functional effects of Adrb2 on the femoral head, Adrb2 global knockout (*Adrb2*^*−/−*^) mice were generated and treated with RU486 in the PVN to stimulate sympathetic outflow during GC-induced ONFH (Fig. 5a). Adrb2 was successfully deleted in the femoral heads of the mice, as evidenced by immunostaining and qRT-PCR analysis of ECs (Fig. 5b–d). μCT-reconstructed images of the femoral heads and trabecular bone microarchitectural parameters revealed that Adrb2 deletion significantly increased MPS-induced trabecular bone deterioration and abolished the beneficial effects of sympathetic outflow on the femoral head, with lower levels of Tb. BV/TV, Tb. Th, and Tb. N and higher levels of Tb. Sp relative to those of the *Adrb2*^*WT*^ mice treated with vehicle or RU486 in the PVN (Fig. 5e, f); these results suggest sympathetic outflow blockage of the hypothalamic descending pathway after Adrb2 deletion. HE staining revealed that Adrb2 knockout induced a more severe ONFH phenotype in MPS-treated mice, as evidenced by the higher empty osteocytic lacunae in the trabecular bones and much larger areas of aberrant bone marrow structures filled with more anomalous cells, granular eosinophilic material, and fat vacuoles in the *Adrb2*^*−/−*^ mice relative to their wild-type littermates (Fig. 5g); and this phenotype was unable to ameliorate following RU486 treatment in the PVN in the femoral heads of MPS-treated *Adrb2*^*−/−*^ mice (Fig. 5g). Adrb2 knockout further abolished the increase in H-type ECs and osteoblasts in the femoral heads of the MPS-treated mice following RU486 treatment in the PVN, as shown by immunostaining for OCN and co-immunostaining for CD31 and EMCN (Fig. 5h–j). Of note, RU486 treatment in the PVN did not result in any significant changes relative to the vehicle-treated *Adrb2*^*WT*^ mice and vehicle-treated *Adrb2*^*−/−*^ mice, and Adrb2 deletion markedly elevated the levels of Tb. N and lowered the levels of Tb. Sp in the femoral heads of vehicle-treated mice (Fig. S12a, b); immunostaining revealed a significant reduction in H-type vessels in vehicle-treated *Adrb2*^*−/−*^ mice relative to vehicle-treated *Adrb2*^*WT*^ mice, whereas the changes in OCN^+^ mature osteoblasts did not reach significance, regardless of RU486 or vehicle treatment in the PVN (Fig. S12c–e), indicating that Adrb2-mediated angiogenesis-osteogenesis coupling may specifically occur in GC-treated mice. Collectively, these findings suggest that knockout of Adrb2 not only aggravates GC-induced ONFH but also abolishes the beneficial effects of sympathetic outflow on the femoral head.

### The sympathetic neurotransmitter NE stimulates endothelial alternation of glycolysis

**Fig. 6:**
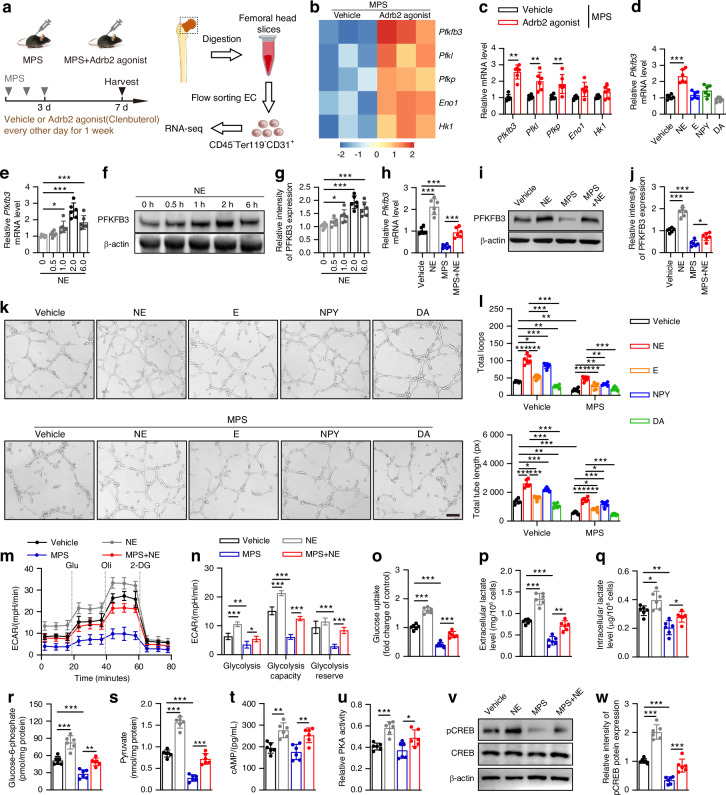
Sympathetic nerves stimulates endothelial alternation of glycolysis**. a** Schematic graph of RNA-seq analysis of the sorted femoral head ECs from vehicle- or MPS-treated mice following Adrb2 agonist (clenbuterol) treatment every other day for 1 week. **b** Heatmap of RNA-seq data showed expression changes encoding glucose metabolism-related genes of the sorted femoral head ECs from vehicle- or MPS-treated mice following Adrb2 agonist (clenbuterol) treatment every other day for 1 week. **c** Quantitative RT-PCR analysis of *Pfkfb3, Pfkl, Pfkp. Eno, and Hk1* genes expression in femoral head ECs from vehicle- or MPS-treated mice following Adrb2 agonist (clenbuterol) treatment every other day for 1 week. **d** Quantitative RT-PCR analysis of *Pfkfb3* gene expression for ECs treated with vehicle, NE, E, NPY, and DA at the concentration of 0.1 μmol/L, respectively. **e** Quantitative RT-PCR analysis of *Pfkfb3* gene expression for femoral head ECs treated with NE for 0–6 h respectively. **f**, **g** Representative images of WB and quantitative analysis of PFKFB3 expression for femoral head ECs treated with NE for 0–6 h respectively. **h** Quantitative RT-PCR analysis of *Pfkfb3* gene expression for femoral head ECs treated with vehicle, NE, MPS, or MPS + NE. **i, j** Representative images of WB and quantitative analysis of PFKFB3 expression for femoral head ECs treated with vehicle, NE, MPS, or MPS + NE. **k, l** Representative tube formation images and quantification of total loops and total tube length of femoral head ECs under different treatments as indicated. Scale bar: 100 μm. **m, n** ECAR profile showing glycolytic function and quantification of glycolytic function parameters for femoral head ECs under different treatments as indicated. Vertical lines indicate the time of addition of glucose (10 mmol/L), oligomycin (1 μmol/L), and 2-DG (50 mmol/L). **o**−**s** Measurement of glucose uptake, extracellular and intracellular lactate levels, and intracellular G-6-P as well as pyruvate levels for femoral head ECs under different treatments as indicated. **t** Quantitative analysis of ELISA assay for cAMP in femoral head ECs in response to different treatments as indicated. **u** Quantitative analysis of PKA activity assay for cell homogenates of femoral head ECs receiving different treatments as indicated. **v, w** Representative images of WB and quantitative analysis of pCREB/CREB expression for femoral head ECs under different treatments as indicated. All data were presented as means ± SD, *n* = 6 per group; *P < 0.05. **P < 0.01. ***P < 0.001. Statistical significance was determined by two-tailed Student’s *t*-test (**c**). Statistical significance was determined by one-way ANOVA with Bonferroni post hoc test (**d, e, g, h, j, n**−**u, w**). Statistical significance was determined by two-way ANOVA with Bonferroni post hoc test (**l**)

To further explore the downstream mechanism by which sympathetic outflow mitigates GC-induced ONFH, we performed transcriptome sequencing analysis of the sorted ECs in the femoral heads of the MPS-treated mice administered vehicle or an Adrb2 agonist (clenbuterol) for 1 week (Fig. 6a). A total of 929 upregulated genes and 1 487 downregulated genes were noted among the differentially expressed genes (DEGs) in the ECs of the MPS-treated mice in response to vehicle or clenbuterol treatment (Fig. S13a, b). Kyoto Encyclopedia of Genes and Genomes (KEGG) analysis indicated that metabolic pathways, glucose metabolism, cAMP signaling, and adrenergic signaling were considerably enriched in DEGs (Fig. S 13c). For glucose metabolism-related genes, RNA-seq data revealed that cotreatment with clenbuterol significantly upregulated the expression of the glycolytic gene *Pfkfb3* in ECs of the MPS-treated mice (Fig. 6b), and the qRT-PCR analysis of sorted ECs confirmed these results. (Fig. 6c). To verify these findings in vivo, we performed co-immunostaining for EMCN and PFKFB3 and showed that cotreatment with an Adrb2 agonist significantly promoted endothelial PFKFB3 expression in the femoral heads of the MPS-treated mice at both 1 and 6 weeks (Fig. S13d, e). To further validate these effects on ECs in vitro, we added different sympathetic neurotransmitters to stimulate femoral head ECs. Intriguingly, only NE markedly promoted *Pfkfb3* gene expression (Fig. 6d). We next added NE and/or MPS to stimulate ECs. Western blotting and qRT-PCR analysis indicated that NE at 0.1 μmol/L promoted PFKFB3 expression within 2 h in a time-dependent manner (Fig. 6e–g); MPS at 100 μmol/L strongly inhibited PFKFB3 expression compared to that of the vehicle-treated ECs, whereas NE cotreatment significantly blocked the inhibition of Pfkfb3 expression in MPS-treated ECs (Fig. 6h–j).

Sympathetic nerves secrete several functional neurotransmitters to affect the formation of new blood vessels.^46^ Thus, we conducted a tube formation assay to determine which of these components was more crucial for angiogenesis regulation in GC-treated ECs. Only NE or NPY treatment resulted in a substantial enhancement in the capacity of femoral head ECs for generating tubes on Matrigel in comparison to vehicle treatment (Fig. 6k, l). Although MPS treatment significantly impeded the ability of ECs to constitute the total length of the tube and the structure of loops and branching points in vehicle-treated ECs, NE or NPY cotreatment had a noticeable proangiogenic role relative to that in the MPS-treated ECs, and many more vascular tube-like forms were observed, as evidenced by the pictures of femoral head ECs alongside the significantly larger total loops and total tube length values following NE cotreatment relative to NPY cotreatment (Fig. 6k, l). Angiogenesis is driven by the glycolytic enzyme PFKFB3 to facilitate endothelial migration and proliferation.^30,31^ Thus, we focused on these metabolic alterations in PFKFB3 to explore the molecular mechanism by which NE stimulates EC angiogenic resistance to MPS-induced endothelial impairment in the femoral head. The results revealed notable inhibitory variations in glycolysis, glycolytic capacity, and glycolytic reserve in the MPS-treated ECs compared to the vehicle-treated ECs, as shown by Seahorse Extracellular Flux analysis, which was used to assess the extracellular acidification rate (ECAR) (Fig. 6m, n). Consistently, the levels of glycolytic metabolites involved in intracellular glucose-6-phosphate, pyruvate, and lactate, as well as extracellular lactate, were all reduced in response to MPS treatment (Fig. 6o–s). NE treatment resulted in essential improvements in glycolytic alterations in both the vehicle- and MPS-treated ECs, as shown by analysis of the ECAR and glycolytic metabolite levels (Fig. 6m–s).

NE regulates the formation of cyclic adenosine monophosphate (cAMP) to promote angiogenic gene expression during physiological and pathological processes,^36,47^ and the activation of protein kinase A (PKA) and subsequent phosphorylation of the transcription factor CREB by PKA mediates the proangiogenic effect of cAMP.^48,49^ Here, ELISAs for cellular cAMP showed that NE-treated ECs had significantly increased cAMP levels relative to the vehicle-treated controls, and NE cotreatment caused notably elevated cAMP levels in response to MPS treatment (Fig. 6t). NE cotreatment also substantially elevated PKA activity in MPS-treated ECs, as shown by the PKA activity assay (Fig. 6u). Intriguingly, CREB phosphorylation was significantly inhibited after MPS treatment relative to that in vehicle-treated ECs, whereas NE cotreatment resulted in higher levels of CREB phosphorylation in MPS-treated ECs (Fig. 6v, w). Similarly, immunofluorescence staining for pCREB demonstrated that treatment of mice with Adrb2 agonist alleviated the inhibition of endothelial CREB phosphorylation in the femoral heads of MPS-treated mice at both 1 week and 6 weeks (Fig. S13f, g). Thus, sympathetic tone can reverse the endothelial glycolytic alterations evoked by GCs as a result of Adrb2-mediated activation of CREB phosphorylation and Pfkfb3 expression.

### NE-induced endothelial metabolic alteration is mediated by the activation of Adrb2/cAMP/CREB/PFKFB3 signaling

**Fig. 7:**
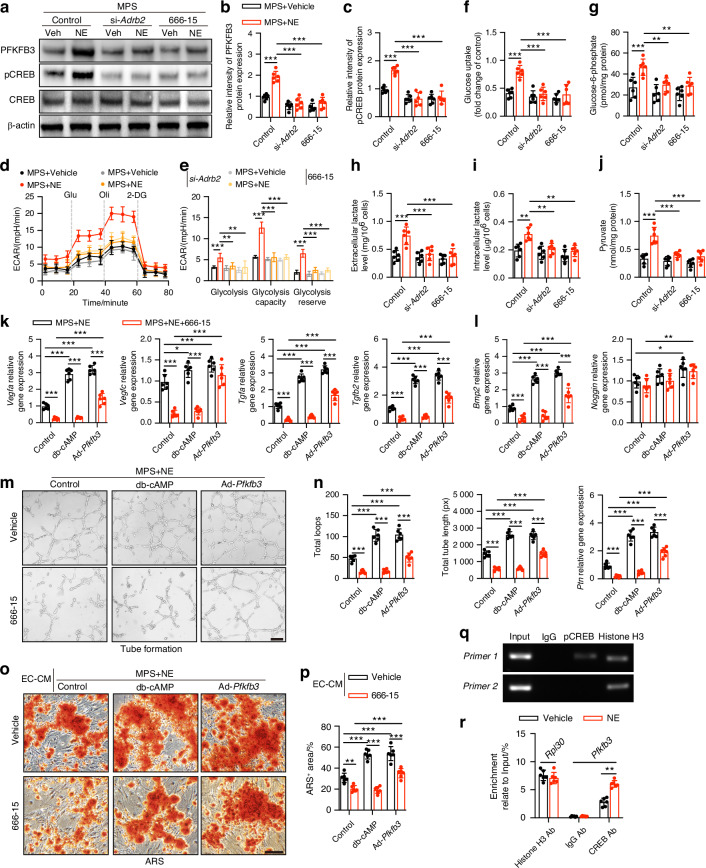
Sympathetic neurotransmitter NE-induced endothelial metabolic alternation and angiogenesis-osteogenesis coupling are mediated by Adrb2/cAMP/CREB/Pfkfb3 signaling. **a**−**c** Representative images of WB and quantitative analysis of PFKFB3 and pCREB/CREB expression for MPS-treated femoral head ECs under vehicle or NE treatment after knockdown of *Adrb2* by si-*Adrb2* or CREB inhibition by 666-15. **d, e** ECAR profile showed glycolytic function and quantification of glycolytic function parameters in MPS-treated femoral head ECs under different treatments as indicated. Vertical lines indicate the time of addition of glucose (10 mmol/L), oligomycin (1 μmol/L), and 2-DG (50 mmol/L). **f**−**j** Measurement of glucose uptake, extracellular and intracellular lactate levels, and intracellular G-6-P as well as pyruvate levels for femoral head ECs under different treatments as indicated. **k, l** Quantitative RT-PCR analysis of pro-angiogenic (*Vegfa, Vegfc, Tgfa, and Tgfb2*) and pro-osteogenic (*Bmp2, Noggin, and Ptn*) genes expression for vehicle or 666-15-treated femoral head ECs with MPS and NE cotreatment following activation of cAMP by db-cAMP or *Pfkfb3* overexpression by Ad-*Pfkfb3*. **m, n** Representative tube formation images and quantification of total loops and total tube length for femoral head ECs under different treatments as indicated. Scale bar: 100 μm. **o, p** Representative images of ARS staining and quantification of the positively stained areas in BMSCs after EC-CM treatment. Scale bar: 50 μm. **q, r** ChIP-qRT-PCR analysis revealed CREB antibody (CREB Ab) immune-precipitate *Pfkfb3* promoter domain enrichment relative to the input DNA in femoral head ECs under vehicle or NE treatment. Normal rabbit anti-IgG acted as a negative control. Histone H3 antibody (Histone H3 Ab) pulldown for *Rpl30* gene enrichment acted as a positive control. All data were presented as means ± SD, *n* = 6 per group; *P < 0.05. **P < 0.01. ***P < 0.001. Statistical significance was determined by two-way ANOVA with Bonferroni post hoc test (**b, c, e**−**l, n, p**). Statistical significance was determined by two-tailed Student’s *t*-test (**r**)

We next explored whether Adrb2/CREB signaling plays a crucial role in the alteration of endothelial glycolysis induced by MPS in vitro. Western blotting analysis showed that the increased PFKFB3 expression caused by NE cotreatment in MPS-treated ECs was significantly reduced via knockdown of Adrb2 by small interfering (si)-RNA or CREB inhibitor (666-15) treatment, and knockdown of Adrb2 blocked NE-induced CREB phosphorylation in MPS-treated ECs (Fig. 7a–c). Seahorse Extracellular Flux analysis revealed that si-*Adrb2* or 666-15 application resulted in reduced glycolysis, glycolytic capacity, and glycolytic reserve in ECs cotreated with MPS and NE (Fig. 7d, e). Similarly, the levels of glycolytic metabolites involved in intracellular glucose-6-phosphate, pyruvate, and lactate, as well as extracellular lactate, in the *Adrb2*-deficient or phospho-CREB-insufficient ECs cotreated with MPS and NE were far less than those in the control ECs (Fig. 7f–j). Si-*Adrb2* or 666-15 application further inhibited PFKFB3 expression, whereas slightly decreased the extracellular acidification rate and the levels of glycolytic metabolites in ECs treated with MPS alone (Fig. 7a–j). These results suggest that blocking Adrb2/CREB signaling could prevent the reversal of glycolytic alterations in MPS-treated ECs following NE treatment.

To further explore whether CREB phosphorylation, which facilitates the transcription of the *Pfkfb3* gene mediated by cAMP-dependent activation, stimulates angiogenesis-osteogenesis coupling, a cAMP analog (db-cAMP) and *Pfkfb3* overexpression adenovirus (Ad-*Pfkfb3*) were applied before MPS and NE cotreatment in femoral head ECs following inhibition of CREB phosphorylation. qRT-PCR analysis revealed significantly higher levels of genes associated with pro-angiogenesis (*Vegfa*, *Vegfc*, *Tgfa*, and *Tgfb2*) and pro-osteogenesis (*Bmp2* and *Ptn*) in the MPS- and NE-cotreated ECs following db-cAMP or Ad-*Pfkfb3* treatment (Fig. 7k, l). *Pfkfb3* overexpression, rather than cAMP activation, significantly reversed the reduction in pro-angiogenic genes (*Vegfa*, *Vegfc*, *Tgfa*, and *Tgfb2*) and pro-osteogenic genes (*Bmp2* and *Ptn*) in phospho-CREB-insufficient ECs cotreated with MPS and NE (Fig. 7k, l). Tube formation assays showed that *Pfkfb3* overexpression led to augmentation of capillary-like network form development on Matrigel in ECs cotreated with NE and MPS and markedly reversed the angiogenic inhibition of phospho-CREB-deficient ECs cotreated with NE and MPS (Fig. 7m). Nevertheless, db-cAMP failed to increase the angiogenic capability of phospho-CREB-insufficient ECs cotreated with MPS and NE, as evidenced by the images of femoral head ECs and much fewer total loops and lower total loop lengths relative to the ECs cotreated with MPS and NE (Fig. 7m, n). We then collected the CM of the MPS and NE-cotreated ECs in response to different treatments and treated mouse BMSCs during osteogenic differentiation. Consistent with the increased levels of pro-osteogenesis genes in ECs, CM from the *Pfkfb3*-overexpressing ECs significantly promoted BMSC osteogenic differentiation in the MPS and NE-cotreated ECs after 666-15 treatment, whereas CM from the db-cAMP treatment did not increase osteogenesis in BMSCs in the MPS and NE-cotreated ECs after inhibition of CREB phosphorylation (Fig. 7o, p). Collectively, these results suggest that the sympathetic transmitter NE stimulates PFKFB3-mediated glycolysis by activating cAMP/CREB signaling to increase angiogenic-osteogenic coupling in GC-treated ECs.

Chromatin immunoprecipitation (ChIP) and qRT**-**PCR assays were further performed to assess the potential binding of the transcription factor CREB to the *Pfkfb3* gene promoter and to evaluate whether NE promotes the binding of CREB to *Pfkfb3*. Results revealed that the CREB antibody in the vehicle-treated ECs, instead of the anti-IgG control, significantly enriched the promoter regions of *Pfkfb3* compared to the input DNA (Fig. 7q, r). Treatment with NE significantly enhanced the enrichment of the *Pfkfb3* promoter by the CREB antibody (Fig. 7q, r). These findings indicate that CREB can bind to the promoter regions of the *Pfkfb3* gene, and an increase in *Pfkfb3* gene expression in ECs stimulated by NE is facilitated by increasing the binding of CREB to its promoters.

### Overexpression of PFKFB3 is required for protection against GC-induced ONFH in Adrb2 KO mice

**Fig. 8:**
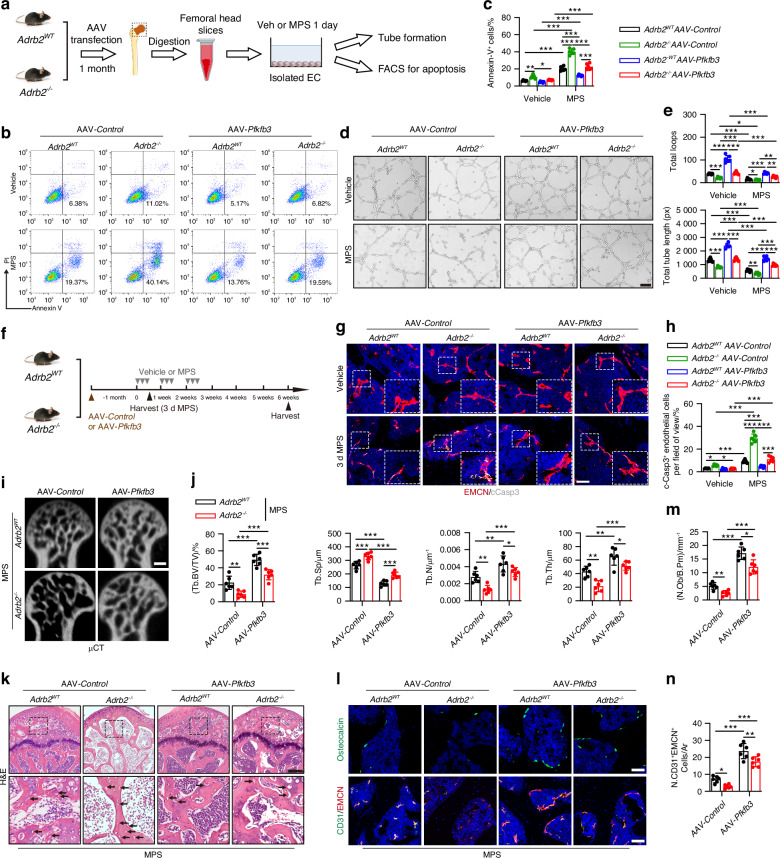
Overexpression of endothelial Pfkfb3 attenuates ONFH in GC-treated *Adrb2*^*WT*^ *and Adrb2*^*−/−*^ mice. **a** ECs were isolated from the femoral heads of *Adrb2*^*WT*^ *and Adrb2*^*−/−*^ mice following adeno-associated viral (AAV)-*Control* or AAV-*Pfkfb3* transfection for 1 month. Schematic diagram showing the procedure of flow cytometry for apoptosis and tube formation assays. **b**, **c** Representative images and quantification of flow cytometry analysis of Annexin V/PI staining in femoral head ECs from different mice as indicated. **d**, **e** Representative tube formation images and quantification of total loops and total tube length of femoral head ECs from different mice as indicated. Scale bar: 100 μm. **f** Experimental design graph for exploring the effects of endothelial PFKFB3 overexpression on the femoral heads in MPS-treated *Adrb2*^*WT*^ *and Adrb2*^*−/−*^ mice. Mice were harvested 3 days after MPS daily injection or 6 weeks after the first injection of MPS. One down arrow (grey) represented one time vehicle or MPS treatment, and brown arrow represented AAV transfection. **g**−**n** MPS-treated *Adrb2*^*WT*^ *and Adrb2*^*−/−*^ mice were grouped as AAV-*Control* and AAV-*Pfkfb3* transfection. **g, h** Representative immunofluorescence co-staining of EMCN (red) and c-Caspase-3 (white) and quantitative analysis for vessels expressing both EMCN and c-Caspase-3 in the femoral heads of 3-days MPS-treated *Adrb2*^*WT*^ *and Adrb2*^*−/−*^ mice and their littermates were received AAV-*Control* and AAV-*Pfkfb3* transfection. Scale bar: 50 μm. **i, j** μCT reconstruction images and quantitative analysis of Tb. BV/TV, Tb. Th, Tb. N and Tb. Sp of femoral heads. Scale bar: 1 mm. **k** H&E staining images of femoral heads. Scale bar: 200 μm. Black arrows indicate empty osteocytic lacunae. **l** Representative images of immunofluorescence staining of OCN (green) and co-staining of CD31 (green) with EMCN (red) (**m, n**) and quantitative analysis of the number of OCN^+^ osteoblasts and CD31^+^EMCN^+^ cells in the femoral heads. Scale bar: 50 μm. All data were presented as means ± SD, *n* = 6 per group; *P < 0.05. **P < 0.01. ***P < 0.001. Statistical significance was determined by two-way ANOVA with Bonferroni post hoc test

To explore the role of PFKFB3 in the regulation of endothelial homeostasis in vivo, we generated adeno-associated virus vectors (Tie2-AAV) to selectively overexpress endothelial PFKFB3 in *Adrb2*^*WT*^ and *Adrb2*^*−/−*^ mice before GC-induced ONFH establishment, in which 1 × 10^11^ vector genomes (vg) (100 µL) of AAV-*Control*-GFP-FLAG or AAV-*Pfkfb3*-GFP-FLAG were intravenously injected into the tails of mice (Fig. S14a). One and three months after the first tail vein injection, we assessed the transfection of AAV to ensure PFKFB3 overexpression in the endothelium. The results confirmed that the fusion protein (FLAG) was expressed in the isolated ECs of the AAV-*Pfkfb3* mice compared to the AAV-*Control* mice, and the endothelium-predominant transfection was further evidenced by co-immunostaining for EMCN and GFP and co-immunostaining for EMCN and PFKFB3 in the femoral heads (Fig. S14b–e); these results suggest long-term endothelial Pfkfb3 overexpression in the femoral heads of mice. We isolated femoral head ECs from *Adrb2* knockout mice after endothelial PFKFB3 transfection for 1 month, and the cells were then treated with vehicle or MPS (Fig. 8a). MPS treatment led to an increase in the apoptosis of femoral head ECs, as evidenced by the higher proportion of Annexin V^+^ cells relative to vehicle-treated ECs from both *Adrb2*^*WT*^ mice and *Adrb2*^*−/−*^ mice, and MPS-treated *Adrb2*^*−/−*^ ECs had a higher percentage of apoptosis relative to MPS-treated *Adrb2*^*WT*^ mice (Fig. 8b, c). However, the level of Annexin V^+^ apoptotic cells was significantly decreased in AAV-*Pfkfb3* MPS-treated ECs from both *Adrb2*^*WT*^ mice and *Adrb2*^*−/−*^ mice (Fig. 8b, c). A tube formation assay revealed that the reduction in capillary-like network structure formation on Matrigel in MPS-treated ECs from *Adrb2*^*−/−*^ mice was reversed after endothelial PFKFB3 overexpression (Fig. 8d, e). We then evaluated whether endothelial impairment in the early stage induced by MPS could be ameliorated in AAV-*Pfkfb3* mice (Fig. 8f). Indeed, MPS treatment daily for 3 days in *Adrb2*^*−/−*^ mice resulted in an increase in the number of c-Caspase3^+^EMCN^+^ double-positive cells relative to *Adrb2*^*WT*^ mice, whereas the accumulation of these apoptotic ECs was markedly reduced in AAV-*Pfkfb3* mice relative to AAV-*Control* mice (Fig. 8g, h). These results suggest that targeting endothelial PFKFB3 can mitigate endothelial impairment and stimulate angiogenesis in the femoral head in response to GC treatment at the early stage regardless of whether the sympathetic pathway is blocked.

**Fig. 9:**
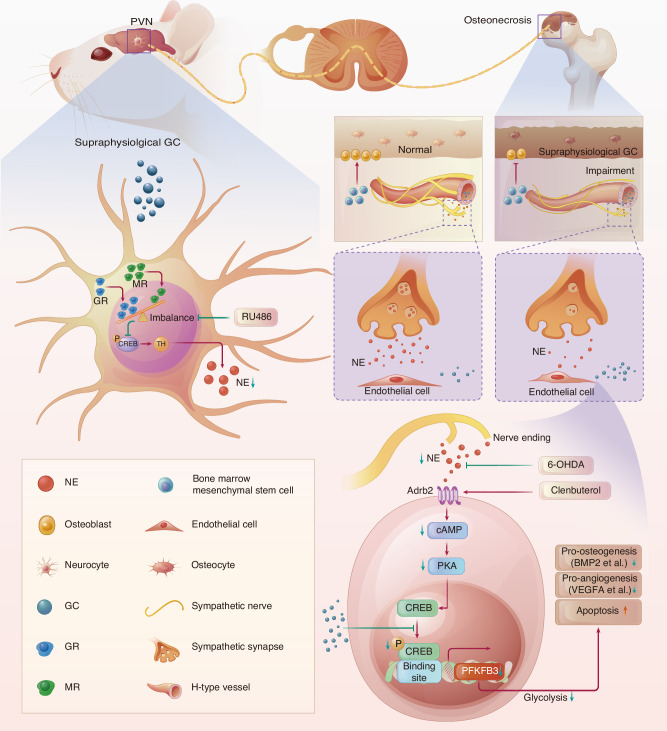
Schematic diagram showing a novel mechanism in the pathogenesis of GC-induced ONFH by which hypothalamic sympathetic descending pathway regulate endothelial metabolism-driven angiogenesis and osteogenesis coupling via transferring NE to the femoral head

We then investigated whether endothelial PFKFB3 overexpression in vivo was sufficient to attenuate microarchitecture destruction in the femoral head in response to GC treatment at the late stage (Fig. 8f). Intriguingly, AAV-*Pfkfb3* transfection led to much higher Tb. BV/TV, Tb. Th, and Tb. N values, as well as lower Tb. Sp values in both the MPS-treated *Adrb2*^*WT*^ mice and MPS-treated *Adrb2*^*−/−*^ mice relative to the AAV-*Control* transfection, as evidenced by μCT-reconstructed images of the femoral heads and trabecular bone microarchitectural parameter analysis (Fig. 8i, j), suggesting a bone-beneficial influence on the femoral head by PFKFB3 overexpression in the endothelial cells. H&E staining showed that AAV-*Pfkfb3* transduction in both *Adrb2*^*WT*^ and *Adrb2*^*−/−*^ mice significantly diminished empty osteocytic lacunae abundance in trabecular bone and reduced the number of cellular lesions with atypia in marrow structures caused by MPS treatment relative to AAV-*Control* mice (Fig. 8k). Furthermore, endothelial PFKFB3 overexpression in mice notably increased the number of H-type ECs and OCN^+^ mature osteoblasts in the femoral heads in response to MPS treatment, regardless of whether Adrb2 was knocked out, as evidenced by immunofluorescence staining for OCN (Fig. 8l, m) and co-immunofluorescence staining for CD31 and EMCN (Fig. 8l, n). Of note, endothelial PFKFB3 overexpression in vehicle-treated mice induced the augmentation of trabecular bone microarchitecture, H-type ECs, and OCN^+^ mature osteoblasts in both *Adrb2*^*WT*^ mice and *Adrb2*^*−/−*^ mice, as evidenced by μCT analysis and immunofluorescence assays, respectively (Fig. S15); this result suggests enhanced angiogenesis-osteogenesis coupling in the femoral head under either normal or GC-induced microarchitecture deterioration. To summarize, these findings reveal the potential therapeutic role of endothelial PFKFB3 in GC-induced ONFH treatment. The graphical abstract reveals the sympathetic tone regulation of endothelial homeostasis in GC-induced ONFH through the hypothalamic descending pathway (Fig. 9).

## Discussion

GC has been widely used for their anti-inflammatory and immunosuppressive effects. Nevertheless, GC use is associated with a high risk of numerous complications, including ONFH and CNS disorders.^50^ The pathophysiological mechanism of GC-induced ONFH remains to be fully elucidated. Growing knowledge reveals that the CNS as an executor by integrating and interpreting information from both external and internal sources.^24^ It then relays a coordinated response through descending sympathetic nerves and neuroendocrine regulation to directly maintain bone homeostasis.^51–54^ Here, our data showed that GC-induced SNS dysfunction was associated with the pathogenesis of osteonecrosis. Briefly, a high dose of GCs suppressed sympathetic outflow by impeding the balance of GR and MR activation in the hypothalamic PVN, thus leading to metabolic alterations and vascular endothelial injury, and knockout of Adrb2 aggravated GC-induced ONFH. We demonstrated that regulation of the sympathetic tone could promote H-type vessel angiogenesis coupled with osteogenesis in the femoral head and ameliorate GC-induced ONFH via the brain-bone axis.

Conventionally, vascular endothelial injury is thought to be the key determinant in the early stage of GC-induced ONFH, and GC tend to induced endothelial cell apoptosis in the early stage of ONFH.^8–10^ We observed that endothelial apoptosis occur exclusively at the femoral head but not at the liver, kidney, or distal femur in response to GC treatment daily for 3 days, indicating that femoral head vessels are more vulnerable than vessels in other tissues during the early stage of GC-induced ONFH. The SNS innervates peripheral organs and maintains physiologic homeostasis by preparing the body for ‘fight or flight’ responses.^55^ SNS dysfunction are characterized by a decrease in TH^+^ sympathetic nerves in the femoral heads of both patients and mice with GC-induced ONFH, a gradual decrease in serum NE with the severity of ONFH in patients, and a decrease in TH expression in both the hypothalamic PVN and the sympathetic postganglionic neurons of ONFH mice. More importantly, sympathetic tone inhibition occurred earlier than vascular endothelial injury in the early stage of GC-treated mice, whereas the bone remodeling was not altered during this period, suggesting that GC-induced sympathetic tone inhibition may be upstream of endothelial injury. In addition to NE, sympathetic tone is fulfilled by releasing other neurotransmitters to regulate endothelial homeostasis, such as E, DA, and NPY.^35,46^ Our results showed that NE, the most abundant sympathetic neurotransmitter, was the essential marker reflecting sympathetic tone changes and ameliorated angiogenesis inhibition in response to GC treatment. Decreased NE levels were observed in both the serum and bone marrow of the femoral head but not the adrenal medulla in GC-induced ONFH mice, suggesting that NE in the femoral head may be mainly derived from the release of postganglionic sympathetic endings.

The PVN of the hypothalamus is crucial in regulating sympathetic outflow for bone homeostasis.^11^ Excessive activation of GR or insufficient activation of MR promotes neuronal apoptosis and dendritic atrophy, which lead to cognitive, emotional and neuroendocrine disorders.^22^ GR and MR interact and even form heterodimers, they may function in complementary or opposite ways depending on the microenvironment.^56,57^ MR contributes to maintaining the physiological sympathetic activities.^58^ Our results firstly showed that an imbalance in GR and MR activation occurred in mice with GC-treated ONFH. Specifically, GCs promoted nuclear GR expression and inhibited nuclear MR expression. Inhibition of GR overactivation by RU486 treatment in the PVN stimulated sympathetic outflow and partially restored the suppressed sympathetic tone. Moreover, the number of H-type vessels and osteoblasts in the femoral head was increased in MPS-treated mice after RU486 treatment, indicating that these beneficial effects may occur mainly through H-type vessel angiogenesis coupled with osteogenesis. Indeed, HPA axis also contributes to PVN-mediated sympathetic outflow and facilitates NE secretion from adrenal medulla under stress condition,^59^ but MPS treatment was insufficient to result in significantly lower NE levels in adrenal medulla in GC-treated mice. This could be related to the differences in the effects of corticosterone and exogenous GCs on the expression of phenylethanolamine N-methyltransferase (PNMT), which could disturb the turnover rate of catecholamines in adrenal medulla.^60^ The selective adrb2 agonist were used to mimic the activation of sympathetic outflow, with unchanged activity of HPA axis in our study. As fat accumulation at the necrotic regions of the femoral head is also related to GC-induced osteonecrosis, we eliminated the classical ascending pathway to determine if leptin, mainly secreted by adipocytes, might inhibit bone formation by hyperactivation of sympathetic tone in the hypothalamus.^40^ We found that GCs inhibited sympathetic tone predominantly due to the imbalance of nuclear GR and MR activation in the PVN of the hypothalamus.

Sympathetic denervation in bone inhibits the activation of HSCs and expedites their senescence;^61^ sympathetic denervation also accelerates surgically induced arthritis and impaired the bone remodeling in healthy adult mice.^62,63^ Recent research has found decreased sympathetic nerves in the femoral heads of patients with GC-induced ONFH, but ignored the role of sympathetic nerves within femoral heads^20^ We found that sympathetic denervation did not induce osteonecrosis in healthy mice but eliminated the beneficial effect of increased sympathetic outflow on the femoral heads and further aggravated osteonecrosis in MPS-treated mice. These results suggest that femoral head homeostasis is highly relay on sympathetic innervation, and sympathetic denervation compromises the spatiotemporal control of the femoral head by hypothalamic-sympathetic descending pathway. However, our data showed that stimulating sympathetic outflow did not relieve bone destruction in the femoral heads of the mice in which GC-induced osteonecrosis and sympathetic denervation had already been initiated. The reasons for sympathetic nerve impairment following vascular injury in the femoral head may be multifaceted, such as nerve ischemia and axon dysfunction.^64^ Preventing and ameliorating GC-induced osteonecrosis development is challenging, as sympathetic nerve damage may expedite this process. Due to the complex interactions between the femoral head and sympathetic nerve, future studies determining the neuropathy time point in GC-induced ONFH and the specific type of skeletal cells beyond ECs that may communicate with the sympathetic nerve are expected.

Clinical research revealed that patients with traumatic brain injury showed increased sympathetic outflow and accelerated fracture healing, whereas blockade of sympathetic adrenergic receptors delayed the healing process.^25,65,66^ Increased sympathetic tone augments mobilization and homing of endothelial progenitor cells (EPCs) to promote wound healing.^67^ It has also been reported that non-selective β-adrenergic receptors agonist stimulates the migration, proliferation and tube formation of ECs, while only blockade of α-adrenergic receptors has no effects on vessel density.^68^ Our data showed that the expression of *Adrb2* was the most abundant in ECs isolated from the femoral head among all adrenergic receptors, and higher levels of *Adrb2* mRNA were observed in H-type ECs of the femoral head. Adrb2 is a component within the NE receptor, which change the sensitivity of cells to NE.^69^ Among bone-related cells, only ECs in the early stage in MPS-treated mice had higher *Adrb2* mRNA levels than the controls, indicating that elevated expression of Adrb2 in ECs in the femoral head is compensated to the decreased sympathetic tone and increase sympathetic sensitivity. Adrb2 signaling has a wide range of functions, helps with facilitating metabolism homeostasis and promoting angiogenesis.^35^ Chronic stress promotes tumor growth and angiogenesis by activating Adrb2 signaling.^33,34^ Here, Adrb2 agonist treatment alleviated GC-induced osteonecrosis and promoted H-type vessel angiogenesis coupled with osteogenesis. Knockout of Adrb2 accelerated microarchitectural destruction of the femoral heads of MPS-treated mice. The beneficial effects of maintaining the GR and MR balance by RU486 treatment were canceled in MPS-treated *Adrb2*^*−/−*^ mice. However, Adrb2 activation mitigated GC-induced osteonecrosis in mice with sympathetic denervation. These findings indicate that Adrb2 is the crucial therapeutic target for coordinating the sympathetic tone to regulate endothelial homeostasis and alleviate GC-induced ONFH. Although hyperactivation of sympathetic tone stimulates bone resorption and bone loss via Adrb2 signaling in osteoblasts, clinical findings of blockade of β-adrenergic receptors to prevent bone destruction are still contradictory.^70–72^ Intriguingly, RU486 treatment rescued the significantly decreased NE levels in the serum and femoral head to a relative normal level in MPS-treated-mice, which facilitated the skeleton and endothelial homeostasis in femoral head. This evidence suggests that GC-induced excessively decreased sympathetic tone contributes to bone deterioration and osteonecrosis due to endothelial impairment.

Angiogenesis requires a large amount of energy to stimulate the ECs proliferation.^27,28^ Sympathetic nerves act as an angio-metabolic switch to help maintain endothelial energy balance, and sympathetic denervation hinders glycolysis and angiogenesis.^35^ PFKFB3 is an important glycolytic enzyme and affects several aspects of cellular homeostasis, including ATP production, autophagy, and redox balance.^73,74^ Notably, endothelial PFKFB3-mediated glycolysis is also necessary for angiogenesis.^30,31^ Our findings demonstrated that PFKFB3 is a key molecule within sympathetic mediated regulation of endothelial metabolism and the improvement of GC-induced vessel injuries. Activation of the sympathetic tone stimulated glycolytic flux, PFKFB3 expression, and endothelial angiogenesis. Adrb2 is a family of G-protein-coupled receptors that can trigger cAMP tone and the phosphorylation of CREB signaling.^48,49^ NE binds to adrenergic receptors to stimulate the expression of angiogenic genes in ECs and osteoblasts.^35,72^ NE also triggered PFKFB3 expression and alleviated MPS-induced glycolytic inhibition via Adrb2-mediated CREB phosphorylation in femoral head ECs. In addition, PFKFB3 overexpression in ECs significantly alleviated endothelial apoptosis at the early stage, mitigated GC-induced microarchitectural damage, and promoted H-type vessel angiogenesis coupled with osteogenesis in the femoral heads of the MPS-treated *Adrb2*^*WT*^ and *Adrb2*^*−/−*^ mice. These results suggest that PFKFB3 may be the key target for the sympathetic regulation of endothelial homeostasis and the amelioration of GC-induced ONFH.

GCs are the external signals sensed and transmitted to the multiple nuclei within the brain, including the hypothalamus, to coordinate sympathetic tone.^11^ Nevertheless, the central projection arrangement regulating skeletal homeostasis is still poorly understood. Due to GR and MR expression in other brain regions, such as the hippocampus, coeruleus, amygdala, and striatum, we cannot rule out the possibility that GC alters the reciprocal projection effects of other regions to the PVN regulating sympathetic outflow.^56,57,75,76^ Furthermore, other neurotransmitters released from sympathetic nerves also play a role in femoral head ECs. The complicated regulatory effect of sympathetic nerves on femoral head homeostasis is still expected to investigation. Skeletal interoception regulates bone homeostasis by sensing skeletal microenvironmental changes via the sensory nerve PGE2-EP4 ascending pathway.^24^ Further studies focusing on skeletal interoception mediated regulation of femoral head homeostasis will lead us to explore novel mechanisms and effective therapies for GC-induced ONFH. In summary, we demonstrated that sustaining the sympathetic tone via the PVN of the hypothalamus alleviated endothelial impairment in the early stage following GC treatment. The increased sympathetic tone stimulates endothelial glycolysis via the Adrb2/CREB/PFKFB3 signals and promotes H-type vessel angiogenesis coupled with osteogenesis, thereby delaying and protecting against GC-induced osteonecrosis.

## Materials and methods

### Collection and grading of femoral head tissue samples

Informed consent to obtain human femoral head tissue was provided by all patients involved in the total hip arthroplasty surgery. The experimental methods were conducted in compliance with the approved guidelines, and the study was authorized by the ethics committee of Wuhan Union Hospital, Huazhong University of Science and Technology (Approval number: 2021-IEC-S016). All participant data are shown in Table S1. The inclusion criteria for each chosen patient with ONFH were exposure to prednisolone over 1800 mg or a threshold of 4 weeks.^77^ ARCO Stages I to IV were used to evaluate the degree of ONFH,^78^ and the comprehensive histological staging system is listed in Table S2. The diagnosis of ONFH was confirmed by X-ray and MRI (FS T2W) images. Human peripheral blood samples were obtained as described previously.^79^ Blood samples were taken from healthy controls and patients with different degrees of ONFH (ARCO stage I to IV) who had laid in a quiet dark room supine for at least 30 minutes in the morning, following a night of rest and a midnight fast. For neurotransmitter measurement, the blood was transferred to a chilled vacuum tube with EDTA and then centrifuged at 1 000 × *g* for 20 min at 4 °C to collect serum. Human femoral head tissues were obtained from patients requiring total hip arthroplasty who developed late-stage ONFH (ARCO stage III to IV) and were diagnosed with femoral neck fracture within 24 h. Normal tissue from patients with femoral neck fractures was used as the control. Exclusion criteria included patients with hematological disorders, malignant tumors, hyperlipemia, poorly managed diabetes (hemoglobin A1C > 8%), infectious illnesses, and immunodeficiency.

### Animals and in vivo treatments

All animal experiments were carried out following the Guidelines of the Experimental Animal Ethics Committee of Tongji Medical College, Huazhong University of Science and Technology (Approval number: S3215). 8-, 11-, or 12-week-old male C57BL/6 J mice weighing 20–24 g were utilized in this study. They were divided into different treatment groups at random. With a consistent 12-hour light/dark cycle and unrestricted access to food and water, every mouse was housed in an environment free of pathogens. Adrb2 knockout (*Adrb2*^*−/−*^) and Adrb2 wild-type (*Adrb2*^*WT*^) mice were purchased from Shanghai Model Organism Center (Stock: 190738). For establishment of the GC-induced ONFH mouse model, 12-week-old wild-type mice were administered intramuscularly with 20 mg/kg MPS (Pfizer, NY, USA) into the thigh, alternating sides on the first 3 days of a week for 3 weeks. Wild-type mice that were received vehicle (DMSO) under the identical circumstances were used as controls. To analyze the potential relationships between sympathetic tone, endothelial injury, and bone remodeling in the femoral head, 12-week-old wild-type mice were treated with MPS once a day for 3 days. To evaluate the effects of the sympathetic tone of the PVN on the femoral head, vehicle- or MPS-treated mice aged 12 weeks were injected with RU486 (1 mg/mL) (M8046; Sigma-Aldrich, St. Louis, MO, USA) or vehicle (PBS) into the PVN of the hypothalamus using a guide cannula every other day for 6 weeks. To investigate the role of the sympathetic tone in the femoral heads of mice whose osteonecrosis had already been initiated by GCs, mice at 3 weeks after the first MPS administration received RU486 injection in the PVN or vehicle treatment every other day for 6 weeks. For analysis of the influence of sympathetic denervation on the femoral head, 11-week-old wild-type mice were intraperitoneally injected with 6-OHDA (H4381; Sigma‒Aldrich) (dissolved in saline with 0.02 mg/mL ascorbic acid) at a first dose of 100 mg/kg and a second dose of 250 mg/kg one week before MPS-treated mice received PVN injection of RU486 or vehicle. 6-OHDA treatments were repeated every 4 weeks to maintain sympathetic denervation. To identify the impact of the downstream sympathetic receptor on the femoral head, clenbuterol, an Adrb2 agonist, was administered subcutaneously at a dose of 2 mg/kg every other day for 6 weeks to activate sympathetic signaling in MPS-treated wild-type mice after sympathetic denervation by 6-OHDA or vehicle. *Adrb2*^*−/−*^ or *Adrb2*^*WT*^ mice received RU486 or vehicle treatment in the PVN before MPS-induced ONFH model establishment. To examine the function of endothelial PFKFB3 in the femoral head during the period of the suppressive sympathetic tone, rAAV-mTie2-*Pfkfb3*-FLAG-GFP or rAAV-mTie2-*Ctrl*-FLAG-GFP was injected into the tail vein of *Adrb2*^*−/−*^ and *Adrb2*^*WT*^ mice aged 8 weeks before MPS-induced ONFH model establishment. All mice were anesthetized, and the femoral heads were harvested 6 weeks after the first MPS administration.

### Stereotaxic procedure

Mice were given isoflurane gas anesthesia (5% for induction and 1% for upkeep; Baxter, TX, USA) and then placed in a stereotaxic apparatus. After the head was shaved with an electric trimmer, the cannula was inserted with its point positioned 1 mm above the PVN (−0.9 mm rostral, ± 0.2 mm lateral, 3.6 mm below skull surface relative to bregma). Acrylic cement was utilized to fix the guide cannula, while two screws made of stainless steel were firmly inserted into the cranium. At least once per day, the mice were observed for indications of pain and distress, and they were monitored attentively for three days following surgery. For at least two to three days, a topical analgesic (prilocaine/lidocaine cream) was applied to prevent the onset of pain and/or discomfort. Enrofloxacin (5 mg/kg) was administered as an antibiotic if clinical indications of postoperative infection were detected. Prior to PVN injection, mice were given 7 days for recovery. A total of 0.5 μL of the GR antagonist RU486 (1 mg/mL) or vehicle (saline) was infused using a 33-gauge needle (65461-01, Hamilton) attached to a microsyringe aimed at the PVN. A 0.5 μl volume of the GR antagonist RU486 (1 mg/mL) or vehicle (saline) was injected into the PVN (0.25 µL per side) over 2 min. The cannula remained positioned for another 2 min so that the medication could permeate entirely from the tip. To prevent guide cannula clogging, a dummy cannula was placed and covered with a dust cap. Evans blue (2%) was injected to verify the injection location.

### μCT analysis

Mice were euthanized and perfusion-fixed with 4% paraformaldehyde via the left ventricle after being flushed with phosphate-buffered saline for 5 min. Femoral heads were dissected and retained in 4% paraformaldehyde for 2 days. High-resolution microcomputer tomography (μCT) (SkyScan 1176, Bruker, Switzerland) was used to scan the femoral head samples. The scanner parameters were as follows: a 9 μm pixel size, 50 kV, 120 μA, 0.5 mm Al filter, 0.9° angular rotation step, 220 projections, and an exposure time of 4.7 s, with a total scan duration of 19 min. Image reconstruction of the femoral heads was performed by NRecon, v1.6, software (Bruker) and then loaded into DataViewer, v1.56, software (Bruker) to export transaxial images as a dataset in a consistent pose, which were utilized to conduct 3D histomorphometric assessments of the femoral head in CTAn v1.11, software (Bruker). Second, the images were then Gaussian-filtered (radius = 0.5, support = 1) and thresholded (23% of the maximal grayscale value), resulting in binarized images containing only bone and background. The circular region of interest (ROI) for the trabecular bone consisted of 150 slices, beginning 0.25 mm below the subchondral bone and extending to the entire femoral head, excluding the marrow cavity and the cortical shell. CTAn v1.11, software (Bruker) was used to evaluate Tb. BV/TV, Tb. Th, Tb. N, and Tb. Sp from the trabecular bones in the femoral head.

### Histological and immunohistochemical staining

The femoral heads and ganglia of the lumbar sympathetic chain from the L2-L4 spinal levels were collected and fixed with 4% PFA for 2 days. The samples received dehydration with 30% sucrose for 24 h after a week of decalcification in 0.5 mol/L EDTA. They were then embedded in paraffin or optimal cutting temperature compound (Sakura Finetek, Torrance, CA). Sections 4 micrometers thick were used for H&E (C0105; Beyotime, Beijing, China) and TRAP (P0332, Beyotime) staining. Histology staining was carried out in accordance with manufacturer instructions. Sections 10 micrometers thick were prepared for brain tissues in accordance with the Franklin atlas of the mouse brain.^80^ Sections 20 micrometers thick of the ganglia of the lumbar sympathetic chain (L2-L4 spinal levels) were co-stained with NeuN and TH for evaluation of the sympathetic tone changes. Coronal sections 20 micrometers thick of the femoral head tissues were co-stained with CD31 and EMCN for H-type vessels, co-stained with EMCN and c-Caspase-3 for vessel apoptosis, co-stained with EMCN and GFP for transfection efficiency of the AAV vector, and co-stained with EMCN and pCREB or PFKFB3 for endothelial signal activation. Forty-micron-thick sections of the femoral heads were co-stained with TH, CD31 and EMCN for sympathetic nerves and vessels. Immunostaining was performed using the following primary antibodies: TH (1:400, AB152; Millipore, Billerica, USA), GAP43 (1:100, 16971-1-AP, Proteintech), NeuN (1:100, ab177487, Abcam), pCREB (1:100, 9198; Cell Signaling Technology, Danvers, MA), Tubb3 (1:100, 657403, Biolegend), OCN (1:100, ab93876; Abcam, Cambridge, UK), CD31 (1:50, AF3628; R&D System, Minneapolis, MN, USA), EMCN (1:50, sc-65495; Santa Cruz Biotechnology, Santa Cruz, USA), Adrb2 (1:100, 13096-1-AP; Proteintech, Wuhan, China), c-Caspase-3 (1:100, ab32042, Abcam), PFKFB3 (1;100, 13763-1-AP, Proteintech), GFP (1:800, ab13970, Abcam), and FLAG (1:200, ab205606, Abcam). After incubation with primary antibodies overnight, sections were incubated with Alexa Fluor-coupled secondary antibodies (Abcam) for 1 h at room temperature and counterstained with DAPI (G1407; Servicebio, Wuhan, China). Images of sections were captured on a fluorescence microscope (Olympus BX51, Tokyo, Japan) or confocal microscope (Zeiss LSM800, Oberkochen, German) and analyzed using ImageJ software (NIH, USA).

### Collection of the supernatant from the bone marrow of the femoral head

Femoral head samples were obtained from the anesthetized mice. After centrifugation at 800 × *g* for 15 min at 4 °C, bone marrow supernatants were kept at −80 °C until the ELISA test.

### ELISA

Blood samples from mice were immediately collected via cardiac puncture after euthanasia. Serum was obtained by centrifugation at 1 000 × *g* for 20 min at 4 °C and stored at −80 °C. The levels of NE, E, NPY, and DA in human and mouse serum, OCN, CTX, and leptin in mouse serum, and NE, E, NPY, and DA in mouse bone marrow of the femoral head were measured using ELISA kits obtained from Elabscience (Wuhan, China) according to the manufacturer’s protocols. The levels of cellular cAMP were determined by a cAMP ELISA kit (581001; Cayman Chemical, Michigan, USA) according to the manufacturers’ instructions.

### Isolation of sympathetic postganglionic neurons

Both sides of the lumbar sympathetic chain are encased in connective tissue anterior to the vertebral column and posterior to the vena cava and aorta. With the help of a dissection stereoscopic microscope, the ganglia of the lumbar sympathetic chain from the L2-L4 spinal levels of 12-week-old male mice were gently isolated as described previously.^81^ Frozen sections of the ganglia were stained and confirmed histologically. Prewarmed collagenase A (SCR136, Sigma-Aldrich) was used to enzymatically digest ganglia at 37 °C for 20 min. Sympathetic postganglionic neurons were transferred into petri dishes coated with poly-D-lysine and laminin following mild mechanical disruption with a Pasteur pipette. The cells were subsequently cultured in Neurobasal Medium with B27 (A3582801, Gibco) serum‐free supplement and Glutamax‐1 and incubated at 37 °C in a 10% CO_2_ humidified atmosphere.

### Isolation of mouse femoral head ECs

For the isolation of primary ECs, the femoral heads were extracted in sterile Ca^2+^- and Mg^2+^- free PBS, crushed, and digested with collagenase A (SCR136, Sigma-Aldrich) for 30 min at 37 °C to obtain single-cell suspensions. With an EMCN antibody (sc-65495, Santa Cruz) and dynabeads sheep anti-Rat IgG (11035, Thermo Fisher Scientific), femoral head ECs were subsequently sorted by magnetic activated cell separation. On culture dishes coated with fibronectin, isolated femoral head ECs were seeded and cultivated in EBM-2 medium (00190860, Lonza) with 10% fetal bovine serum (FBS) (Gibco, New York, USA), 1% penicillin/streptomycin (P/S) (Gibco), and endothelial supplements and growth factors (CC-4176, Lonza). Femoral head ECs were typically utilized within five passages.

### FACS and flow cytometry assay

Femoral heads from mice were obtained, and the soft tissue was extracted. To obtain a single-cell suspension, femoral heads were crushed and digested in sterile Ca^2+^- and Mg^2+^- free PBS with collagenase A (SCR136, Sigma-Aldrich) for 30 min at 37 °C. Cells were stained with EMCN antibody (sc-65495, Santa Cruz) for 30 min on ice. Following the process of washing and filtration, the cells were stained with PE anti-rat secondary antibody (407407, Biolegend), APC-CD31 (160209, Biolegend), PE-Cy7-Ter119 (116221, Biolegend), and PE-Cy7-CD45 (982310, Biolegend) for 30 min on ice, followed by DAPI staining for 5 min ahead of sorting or assessment using FlowJo software (version 10.8). Total femoral head ECs were identified as CD31^+^CD45^−^Ter119^−^DAPI^−^ cells. Gates were set at >10^4^ log Fl-3 (CD31-APC) and >10^3^ log Fl-4 (EMCN-PE) to distinguish the CD31^hi^EMCN^hi^ cells from the total double-positive cells when plotting and sorting H-type ECs.

### RNA-sequencing analysis

Sorted femoral head ECs from MPS-treated mice treated with vehicle or clenbuterol for 1 week were obtained, and total RNA was collected using an RNAprep Pure Kit (DP432, Tiangen Biotech). Wuhan IGENEBOOK Biotechnology constructed an RNA-seq library and utilized the Illumina HiSeq platform to perform RNA-seq. Basically, the quality of each and every RNA sample was evaluated using the Qsep1 equipment. One microgram of total RNA was used to construct RNA libraries with the VAHTS mRNA-seq v3 Library Prep Kit for Illumina. The steps involved RNA extraction using polyA-selected primers, RNA fragmentation, reverse transcription using random hexamer primers, and 150 nt paired-end sequencing by Illumina NovaSeq 6000. Cutadapt (version 1.11) was used to remove the adaptors and low-quality reads. HISAT2 (version 2.1.0) mapped clean reads to mouse reference transcripts, which permitted no fewer than two mismatches. The genes were aligned to publicly available protein databases such as Pfam (Pfam Protein Families) and UniProt (Swiss-Prot). The analysis included the use of RSEM (version 1.2.6) for estimating transcript abundance and normalizing expression levels to fragments per kilobase of transcript per million fragments mapped (FPKM). With a filter cutoff of adjusted *q* value < 0.05 and ∣log_2_FoldChange∣ > 1, DESeq^2^ was utilized to find genes that were differentially expressed. The R package’s ClusterProfiler was utilized to conduct an enrichment analysis using KEGG (Kyoto Encyclopedia of Genes and Genomes, http://www.genome.jp/kegg/), utilizing a hypergeometric distribution and a 0.05 *q* value threshold. Fisher’s exact test q values were corrected for multiple comparisons by the false discovery rate (FDR).

### EC-CM preparation

Femoral head ECs were cleaned 3 times with PBS after different treatments. Then, EC-CM was obtained after cells were treated with a-MEM (12571063, Gibco) for 24 h. After centrifuging EC-CM for 10 min at 300 × *g* and 30 min at 2 000 × *g*, dead cells and debris were eliminated. An Amicon Ultra4 Centrifugal Filter Unit (Millipore) was used to purify the supernatant by centrifugation at 4 000 × *g* for 10 min. By centrifuging at 4 000 × *g*, the supernatant was concentrated to 200 μL. Using a BCA Protein Assay Kit (Thermo Fisher Scientific, USA), the protein content of EC-CM was determined, and the samples were kept at −80 °C.

### PKA activity assay

Using reagents from a PKA kinase test kit (Abcam, ab139435), EC-CM from several groups was incubated. The absorbance at 450 nm was measured using a Thermo Fisher Scientific microplate reader.

### Metabolic measurements

This protocol was performed as described in a previous publication.^82^ In brief, a total of 1.5 × 10^4^ femoral head ECs were cultured on Seahorse XF96 culture cell plates and incubated overnight at 37 °C in EBM-2 EC growth media (00190860, Lonza) with supplements and growth factors (CC-4176, Lonza). After removing the treatment media on day two, the cultured cells were cleaned in assay media (XF base medium with 2 mmol/L glutamine) and the pH was balanced to 7.4 using 0.1 mol/L NaOH. Plates were treated for one hour at 37 °C in a non-CO2 incubator after the medium was changed to assay media with different treatments. The cells were then assessed with an XFe96 extracellular flux analyzer (Seahorse Bioscience). The test used glucose (10 mmol/L), oligomycin (1 μmol/L), and 2-DG (50 mmol/L) as activators and inhibitors. A High Sensitivity Glucose-6-Phosphate Assay Kit (MAK021, Sigma-Aldrich), a Pyruvate Assay Kit (MAK071, Sigma-Aldrich), and a Lactate Assay Kit (MAK064, Sigma-Aldrich) were utilized following the guidelines provided by the manufacturer to determine the levels of glucose-6-phosphate, pyruvate, and lactate in ECs.

### Tube formation analysis

Tube formation assays were performed by using Matrigel Matrix basement membrane (BD Bioscience). Briefly, femoral head ECs were seeded in EBM-2 EC growth medium (00190860, Lonza) with supplements and growth factors (CC-4176, Lonza) at 37 °C overnight. Fifty microliters of Matrigel were added to 96-well culture plates and the plates were incubated at 37 °C for 30 min to allow for solidification. For analysis of the effects of the sympathetic tone on femoral head EC angiogenesis in vitro, cells were seeded on polymerized gel in plates and treated with vehicle (DMSO), NE (0.1 μmol/L), E (0.1 μmol/L), NPY (0.1 μmol/L), and DA (0.1 μmol/L), either alone or in combination with MPS (100 μmol/L). To determine whether cAMP/CREB signaling or PFKFB3 activation contributes to cell angiogenesis, ad-*Pfkfb3* or the cAMP agonist db-cAMP was applied to ECs before MPS plus NE treatment with or without the CREB inhibitor 666-15. Images were obtained using an optical microscope (Olympus BX51, Tokyo, Japan), and total loops were measured by ImageJ software (NIH, USA).

### Osteogenic differentiation assay

At a density of 1 × 10^5^ cells per well, 48-well plates were seeded with mouse BMSCs at passage 3. Then, fresh osteogenic medium (MUXMT-90021, Cyagen) enriched with EC-CM (300 μg/mL at the protein level) from different coculture groups was added to the cells. Fresh osteogenic medium with the appropriate EC-CM was substituted for the induction medium every other day. As controls, BMSCs were cultured in osteogenic medium without EC-CM or a-MEM medium supplemented with 10% FBS and 1% P/S. The cells were stained with ARS solution (C0148S, Beyotime) to assess matrix mineralization following osteogenic induction for 7 days.

### qRT-PCR

Total RNA was extracted from cultivated cells using TRIzol (15596026, Invitrogen) according to the manufacturer’s instructions. Taq SYBR Green Power PCR Master Mix (A25777, Invitrogen) was performed to qPCR using a Step One Plus real-time PCR system (Applied Biosystems, Foster City, CA, USA); β-actin was amplified as an internal control. An examination of the dissociation curve was done for every experiment. Table S3 lists the primer sequences for qRT-PCR that are compatible with mice.

### Western blot analysis

The procedure for Western blotting was as previously mentioned.^83^ In brief, equal amounts of total and nuclear protein extracts (20 μg) were transferred to PVDF membranes, blocked in 5% milk for 1 h, and incubated with primary antibodies at 4 °C overnight. The membranes were washed three times and then incubated with secondary antibodies for 1 h at room temperature. Subsequently, protein bands were observed by a Bio-Rad scanner after preparation with an enhanced chemiluminescence substrate kit (32132, Pierce Biotechnology). The primary antibodies used were as follows: GR (1:2 000, 24050-1-AP, Proteintech), MR (1:1 000, 2185-1-AP, Proteintech), PFKFB3 (1:1 000, 13763-1-AP, Proteintech), pCREB (1:1 000, 9198, Cell Signaling Technology), CREB (1:1 000, 9197, Cell Signaling Technology), Adrb2 (1:1 000, 13096-1-AP, Proteintech), c-Caspase 3 (1:500, ab32042, Abcam), FLAG (1:1 000, ab205606, Abcam), histone H3 (1:2 000, ab1791, Abcam), and β-actin (1:10 000, 81115-1-RR, Proteintech).

### SiRNA transfection

Mouse Adrb2 siRNA (si-*Adrb2*) and negative control siRNA (si*-Control*) were acquired from RiboBio Company (Guangzhou, China). In brief, femoral head ECs were seeded in 6-well plates, cultured for 24 h, and then transfected with Adrb2 siRNA (100 nmol/L) or negative control siRNA using the RiboFECT CP Transfection Kit (C10511, RiboBio) according to the manufacturer’s protocols. Cells were collected for evaluation after further treatments. The siRNA sequences were as follows: si-*Adrb2*-1: 5’-CCATCCTCATGTCGGTTAT-3’; si-*Adrb2*-2: 5’-GCTGCAGA-AGATAGACAAA-3’; and si-*Adrb2*-3: 5’-GGAAGGAACTGTAGTACAA-3’.

### ChIP assay

ChIP assays were carried out with a commercial kit (26156, Thermo Fisher Scientific). Briefly, femoral head ECs were treated with vehicle or NE (0.1 μmol/L) for 2 h. After being fixed for 10 min with 1% formaldehyde at room temperature, the samples were quenched for 5 min with glycine solution, washed with ice-cold PBS, reassembled in buffer, and digested for 20 min with micrococcal nuclease at 37 °C. ChIP-grade antibodies against CREB (1:50, 9198, Cell Signaling Technology), rabbit IgG (negative control), or histone H3 were used to immunoprecipitate the chromatin fragments, which were then incubated with ChIP-grade protein G magnetic beads at 4 °C for 2 h. The eluted DNA was purified and analyzed using qRT-PCR to evaluate the degree of enrichment of the region of the *Pfkfb3* gene promoter after rinsing. Anti-histone H3 antibody pulldown was utilized as a positive control for the enrichment of the ribosomal protein L30 (*Rpl30*) gene. The mouse primers for qRT-PCR were as follows: mouse-*Pfkfb3*: forward, 5’-CCATTCCACCTGTTGTAAGCC-3’ and reverse, 5’- CACACTCTCTTAGTAAAGTTGCAGA-CC-3’; mouse-*Rpl*: forward, 5’- GAGCACACTGGGTGGACGACTT-3’ and reverse, 5’- CGTTTGAGACTTGAACACCACTTG -3’.

### Adenovirus transduction of ECs

Mouse Pfkfb3 adenovirus (Ad-*Pfkfb3*) and negative control adenovirus (Ad-*Control*) were acquired from Huameng Biotechnology (Wuhan, China). The transduction process involved seeding cells in 6-well plates for 24 h, followed by a 2 h incubation period in basal media with Ad-*Ctrl* or Ad-*Pfkfb3* adenovirus at a multiplicity of infection (MOI) of 50. The cells were replaced with new complete cell media and treated and analyzed as stated within 48 h. For transfection of the mouse endothelium, 8-week-old male *Adrb2*^*WT*^ or *Adrb2*^*−/−*^ mice received a tail vein injection of 100 μL (1 × 10^11^ vg) of solution containing either rAAV-m*Pfkfb3*-Tie2-FLAG-GFP or rAAV-*Ctrl*-Tie2-FLAG-GFP. Briefly, the m*Pfkfb3* ORF was recombined with AAV vectors. After DNA sequencing identification, the m*Pfkfb3* recombinant vectors were cotransfected into AAV-293 cells with Phelper and pAAV-RC to package AAV, and the virus titers were determined. The primers were mouse-*Pfkfb3*: forward, 5’-CGCGGATCCGCCACCATGCCGTTGGAACTGACCC-3’ and reverse, 5’-CCGCTCGAGCTTG-TCATCGTCATCCTTGTAATCGAT-3’. After adenovirus injection for 4 and 12 weeks, femoral head ECs and the femoral heads of mice were harvested to identify overexpression efficiency.

### Statistical analysis

Data are presented as the mean ± SD. An unpaired, two-tailed Student’s *t* test was performed to compare mean differences between two groups. For multiple-group comparisons, one-way analysis of variance (ANOVA) or two-way ANOVA combined with a Bonferroni post hoc correction was used. *P* < 0.05 was considered statistically significant. Statistical analysis was performed using GraphPad Prism 8.0 software. The sample size (*n*) for each statistical analysis is detailed in each figure legend.

## Supplementary information

Supporting Information

p-value table for two-way ANOVA analysis

## Data Availability

The data that support the findings of this study are available from the responding author upon reasonable request.
