# Supplementary material for: Inhibition of sympathetic tone via hypothalamic descending pathway propagates glucocorticoid-induced endothelial impairment and osteonecrosis of the femoral head
Source: Bone Res. 2024 Nov 8;12:64. doi: 10.1038/s41413-024-00371-3 (PMC11549335; doi:10.1038/s41413-024-00371-3)
Supplement: Supplementary file 1 — Supporting Information [file 41413_2024_371_MOESM1_ESM.pdf]

# Supporting Information

**Inhibition of sympathetic tone via hypothalamic descending pathway propagates glucocorticoid-induced endothelial impairment and osteonecrosis**

*Wenkai Shao[#], Bo Wang[#], Ping Wang[#], Shuo Zhang, Song Gong, Xiaodong Guo, Deyu Duan, Zengwu Shao, Weijian Liu, Lei He, Fei Gao, Xiao Lv[*], Yong Feng[*]*

[#]Wenkai Shao, Bo Wang, and Ping Wang contributed equally to this work.

[*]Corresponding author: Yong Feng (fengyong@hust.edu.cn) and Xiao Lv (xlyu@hust.edu.cn).

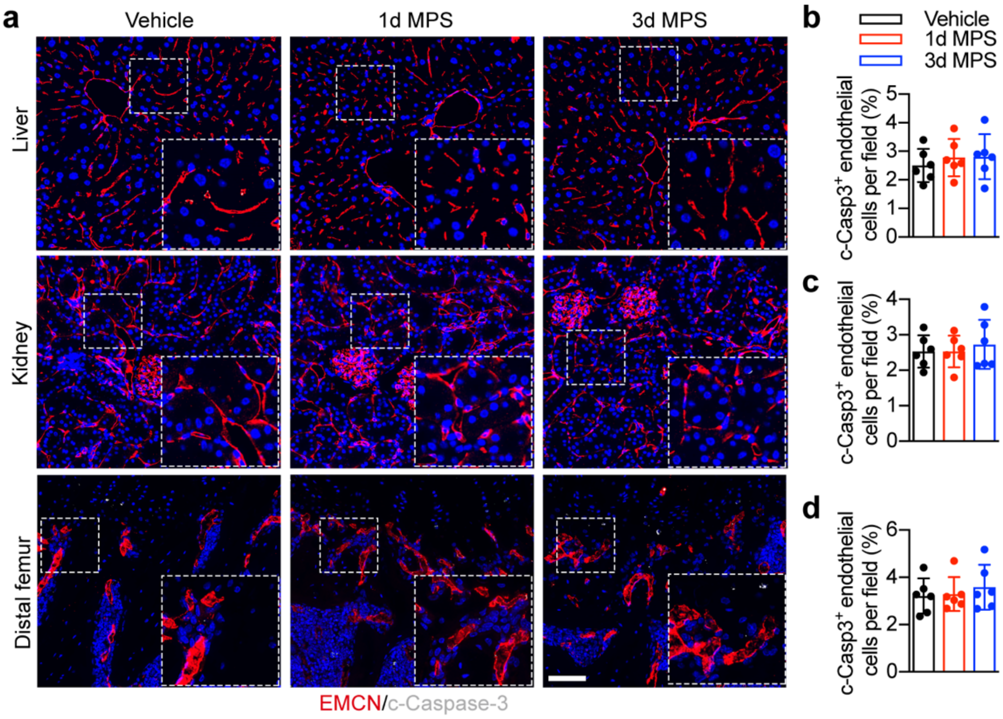


**Fig. S1** Effects of MPS treatment on endothelial apoptosis in distal femur, liver and kidney. **a-d** Representative immunofluorescence co-staining of EMCN (red) and c-Caspase-3 (white) and quantitative analysis for vessels expressing both EMCN and c-Caspase-3 in liver, kidney, and distal femur of mice treated with vehicle or MPS by daily injection for different time periods as indicated. Scale bar: 50 µm. All data were presented as means ± SD, $n = 6$ per group; $^{*}P < 0.05$. $^{**}P < 0.01$. $^{***}P < 0.001$. Statistical significance was determined by one-way ANOVA with Bonferroni post hoc test.

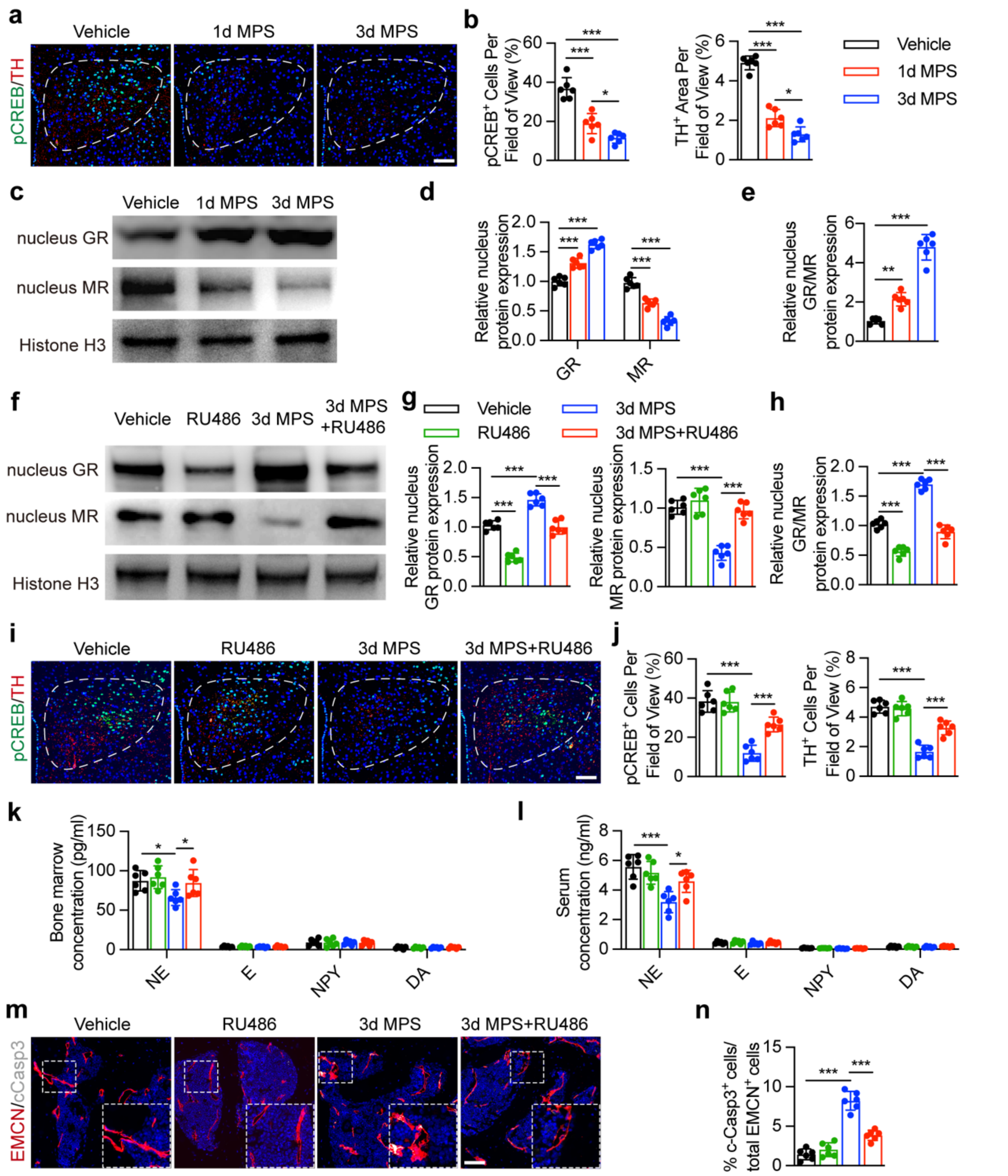


**Fig. S2** GCs undermine hypothalamic sympathetic outflow by disturbing the balance of the nuclear GR and MR in the early stage of MPS-treated mice. **a-b** Representative images of immunofluorescence staining and quantitative analysis of the TH (red) and pCREB (green) in the hypothalamic PVN from mice treated with vehicle or MPS by daily injection for different time periods as indicated. Scale bar: 100 μm. **c-e** Representative images of WB and quantitative analysis of nucleus GR, MR, and Histone H3 expression in the PVN of the hypothalamus from mice treated with vehicle or MPS by daily injection for different time periods as indicated. **f-n** Mice were grouped as vehicle- and MPS-treated mice daily for 3 days and their littermates with vehicle or RU486 treatment in PVN every other day. **f-h** Representative images of WB and quantitative analysis of nucleus GR, MR and Histone H3 expression in the hypothalamic PVN from mice with different treatments as indicated. **i-j** Representative images of immunofluorescence staining and quantitative analysis of the TH (red) and pCREB (green) in the hypothalamic PVN of mice with

different treatments as indicated. Scale bar: 100 µm. **k-l** Quantitative analysis of ELISA assay for NE levels in serum and bone marrow of the femoral heads. **m-n** Representative immunofluorescence co-staining of EMCN (red) and c-Caspase-3 (white) and quantitative analysis for vessels expressing both EMCN and c-Caspase-3 in the femoral heads of mice with different treatments as indicated. Scale bar: 50 µm. All data were presented as means ± SD, $n = 6$ per group; $*P < 0.05$. $**P < 0.01$. $***P < 0.001$. Statistical significance was determined by one-way ANOVA with Bonferroni post hoc test.

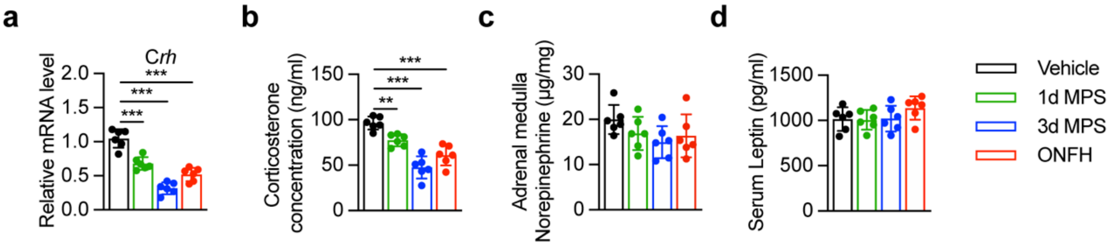


**Fig. S3** Decreased NE levels in the femoral head could be derived from GC inhibitory effects on the sympathetic tone in the CNS. 12-week-old wild-type mice were treated with vehicle and MPS daily for 1 or 3 days, or treated with vehicle and MPS on the first 3 days of a week for 3 weeks and left for another 3 weeks. **a** Quantitative RT-PCR analysis of *Crh* expression for the hypothalamic PVN from mice treated with vehicle or MPS for different time periods as indicated. **b** Quantitative analysis of ELISA assay for serum corticosterone levels from vehicle- or MPS-treated mice for different time periods as indicated. **c** Quantitative analysis of norepinephrine levels in adrenal medulla from vehicle- or MPS-treated mice for different time periods as indicated. **d** Quantitative analysis of ELISA assay for serum leptin levels from vehicle- or MPS-treated mice for different time periods as indicated. All data were presented as means ± SD, $n = 6$ per group; $*P < 0.05$. $**P < 0.01$. $***P < 0.001$. Statistical significance was determined by one-way ANOVA with Bonferroni post hoc test.

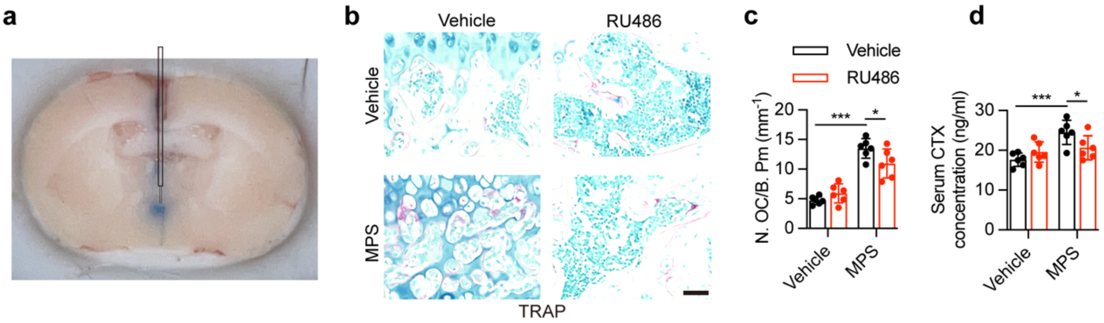


**Fig. S4** An increase in sympathetic outflow by RU486 treatment reduces bone resorption in the femoral heads of MPS-treated ONFH mice. **a** Representative image for identifying the injection sites stained by 2% Evans blue dye from mice injected with vehicle or RU486 (100 nl) in PVN. **b-c** Representative images and quantitative analysis of TRAP staining for the femoral heads from vehicle- and MPS- treated mice and their littermates treated with RU486 or vehicle in PVN. Scale bar: 50 µm. **d** Quantitative analysis of ELISA assay for serum CTX levels in vehicle- and MPS-treated mice and their littermates treated with RU486 or vehicle in PVN. All data were presented as means ± SD, $n = 6$ per group; $*P < 0.05$. $**P < 0.01$. $***P < 0.001$. Statistical significance was determined by two-way ANOVA with Bonferroni post hoc test.

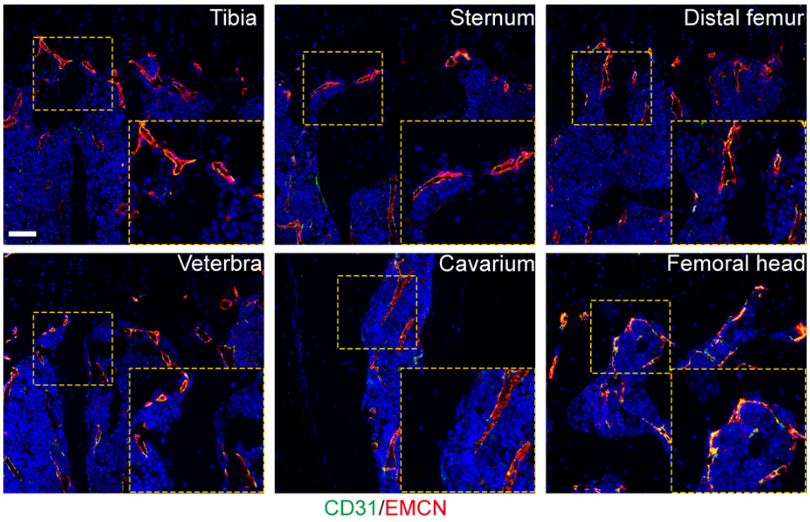


**Fig. S5** H-type vessels are enriched in the femoral heads of adult mice. Representative immunofluorescence co-staining of CD31 (green) and EMCN (red) in tibia, sternum, distal femur, veterbra, cavarium, and femoral head from 12-week-old wildtype mice. Scale bar: 50 µm.

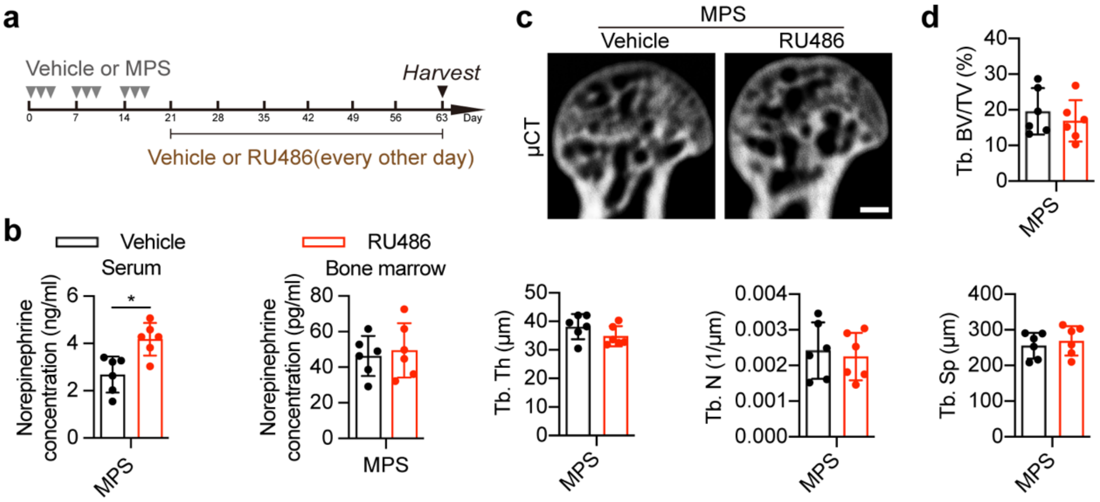


**Fig. S6** Sympathetic outflow has no obvious bone protective effects in the MPS-treated mice. **a** Experimental design graph for exploring the effects of sympathetic outflow on the femoral heads in response to vehicle or RU486 injection in PVN in which MPS-induced ONFH had been developed. Mice were harvested 9 weeks after the first injection of MPS. One down arrow (grey) represents one time vehicle or MPS treatment. **b** Quantitative analysis of ELISA assay for NE levels in serum and bone marrow of the femoral head in MPS-treated mice undergoing vehicle or RU486 treatment. **c-d** µCT reconstruction images and quantitative analysis of Tb. BV/TV, Tb. Th, Tb. N and Tb. Sp of femoral heads in MPS-treated mice undergoing vehicle or RU486 treatment. Scale bar: 1 mm. All data were presented as means ± SD, $n$ = 6 per group; **$P < 0.05$. **$P < 0.01$. ***$P < 0.001$. Statistical significance was determined by two-tailed Student's $t$-test.

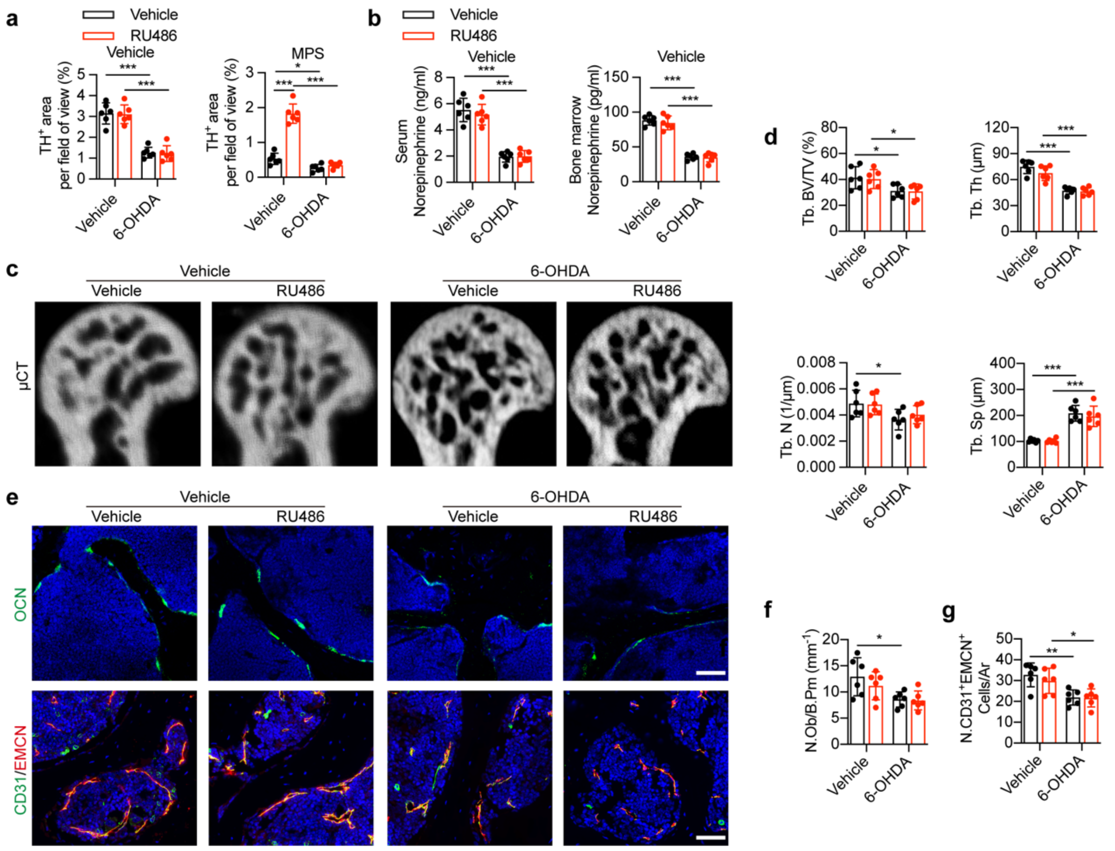


**Fig. S7** Effects of 6-OHDA treatment on the femoral heads of mice following RU486 treatment in PVN. **a** Quantification of TH$^+$ sympathetic nerves for the femoral heads in vehicle- or MPS-treated mice following sympathetic denervation and their littermates with vehicle or RU486 treatment in PVN. **b** Quantitative analysis of ELISA assay for NE levels in serum and bone marrow of the femoral head in vehicle- or 6-OHDA treated mice and their littermates with vehicle or RU486 treatment in PVN. **c-d** μCT reconstruction images and quantitative analysis of Tb. BV/TV, Tb. Th, Tb. N and Tb. Sp of femoral heads. Scale bar: 1 mm. **e** Representative images of immunofluorescence staining of OCN (green) and co-staining of CD31 (green) with EMCN (red) **f-g** and quantitative analysis of the number of OCN$^+$ osteoblasts and CD31$^+$EMCN$^+$ cells in the femoral heads. Scale bar: 50 μm. All data were presented as means ± SD, *n* = 6 per group; **P* < 0.05. ***P* < 0.01. ****P* < 0.001. Statistical significance was determined by two-way ANOVA with Bonferroni post hoc test.

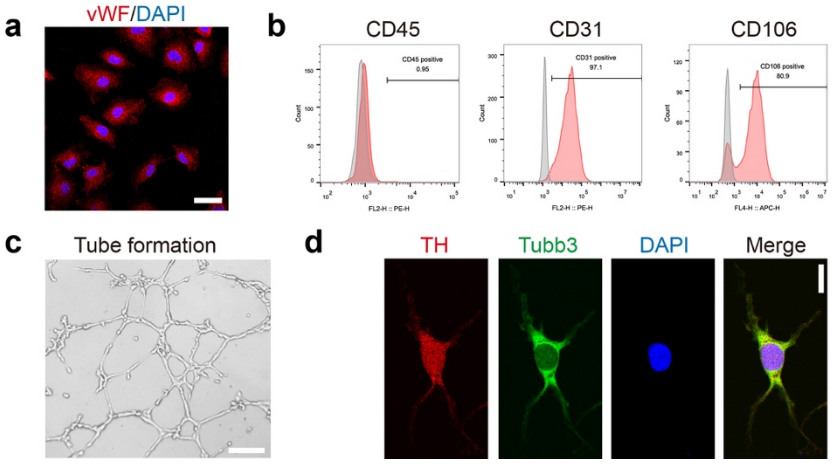

**Fig. S8** Identification of endothelial cells and sympathetic neurons. **a** Representative immunofluorescence image of EC-specific markers (vWF (red)). Scale bar: 50 μm. **b** The purity of the femoral head ECs population was determined by flow cytometry analysis of CD31 and CD106 expression (red: ECs, gray: isotype control). **c** The angiogenesis capacity of femoral head ECs were identified by tube formation assay. Scale bar: 100 μm. **d** Representative immunofluorescence image of sympathetic nerve-specific marker (TH (red)) and neuron-specific marker (Tubb3 (green)). Scale bar: 20 μm.

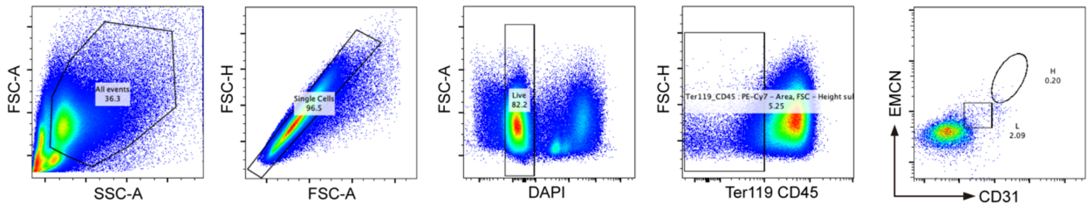


**Fig. S9** Schmatic representation of the strategy used for FACS-sorting of ECs ($DAPI^{-}CD45^{-}Ter119^{-}CD31^{+}$) and type H ECs ($DAPI^{-}CD45^{-}Ter119^{-}CD31^{+}EMCN^{+}$).

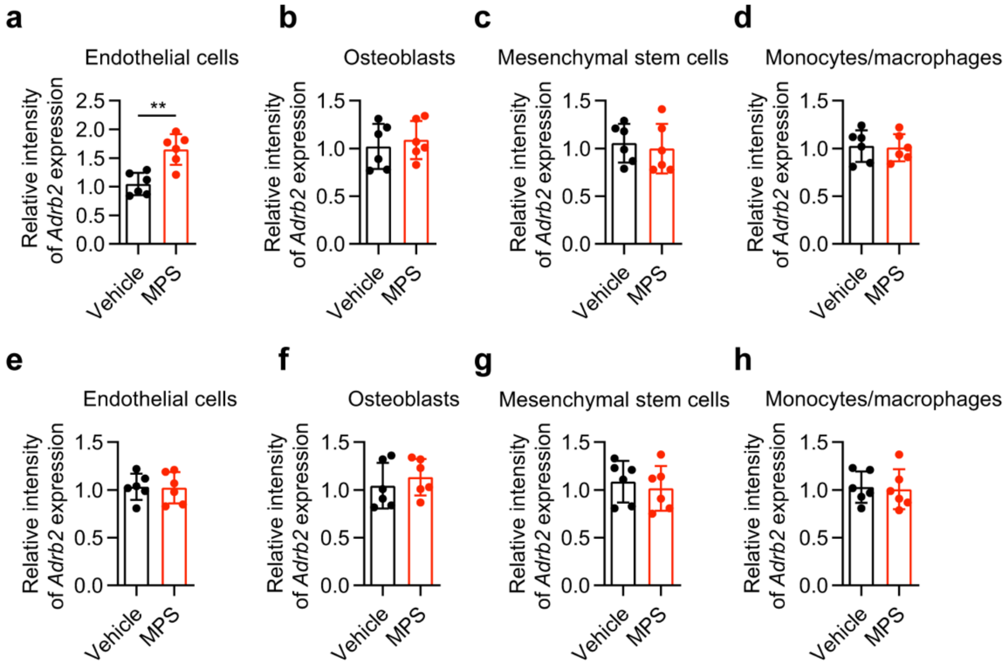


**Fig. S10** *Adrb2* mRNA levels of various cells isolated from the femoral heads of mice. **a-d** Quantitative RT-PCR analysis of *Adrb2* expression for the isolated endothelial cells, osteoblasts, mesenchymal stem cells, and monocytes/macrophages from the femoral heads of mice treated with vehicle or MPS daily for 3 days. **e-h** Quantitative RT-PCR analysis of *Adrb2* expression from the isolated endothelial cells, osteoblasts, mesenchymal stem cells, and monocytes/macrophages following vehicle or MPS treatment in vitro. All data were presented as means ± SD, $n = 6$ per group; $*P < 0.05$. $**P < 0.01$. $***P < 0.001$. Statistical significance was determined by two-tailed Student's *t*-test.

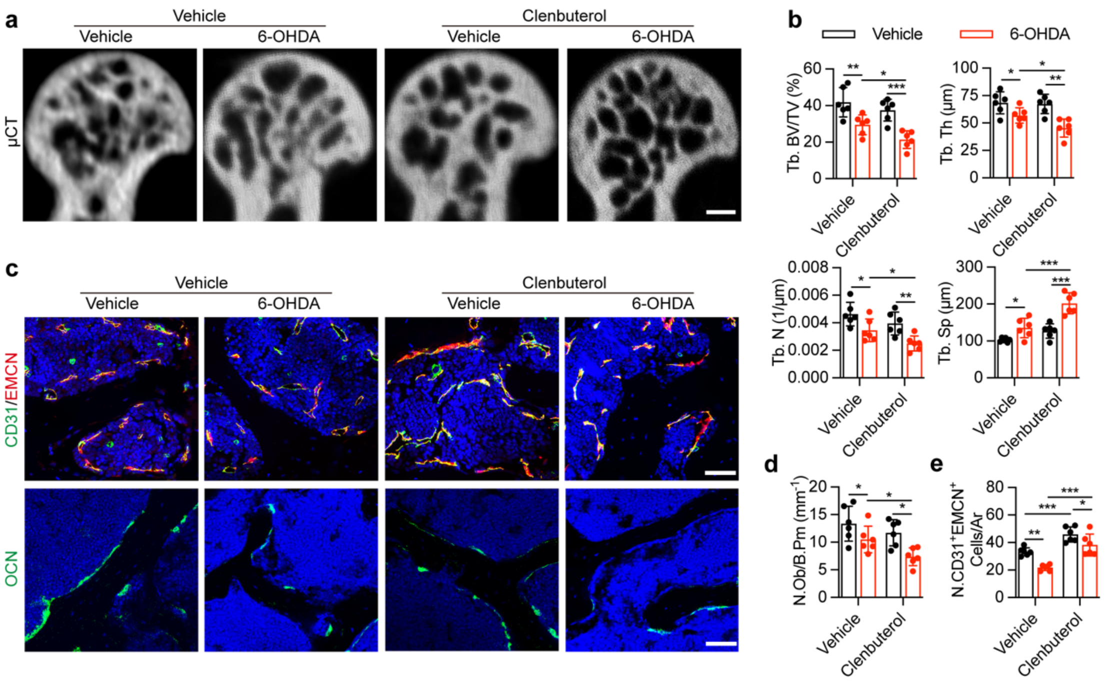


**Fig. S11** Effects of Adrb2 agonist treatment on the femoral heads of mice following sympathetic denervation. **a-b** µCT reconstruction images and quantitative analysis of Tb. BV/TV, Tb. Th, Tb. N and Tb. Sp of femoral heads. Scale bar: 1 mm. **c** Representative images of immunofluorescence staining of OCN (green) and co-staining of CD31 (green) with EMCN (red) **(d-e)** and quantitative analysis of the number of $OCN^+$ osteoblasts and $CD31^+EMCN^+$ cells in the femoral heads. Scale bar: 50 µm. All data were presented as means ± SD, *n* = 6 per group; **P* < 0.05. ***P* < 0.01. ****P* < 0.001. Statistical significance was determined by two-way ANOVA with Bonferroni post hoc test.

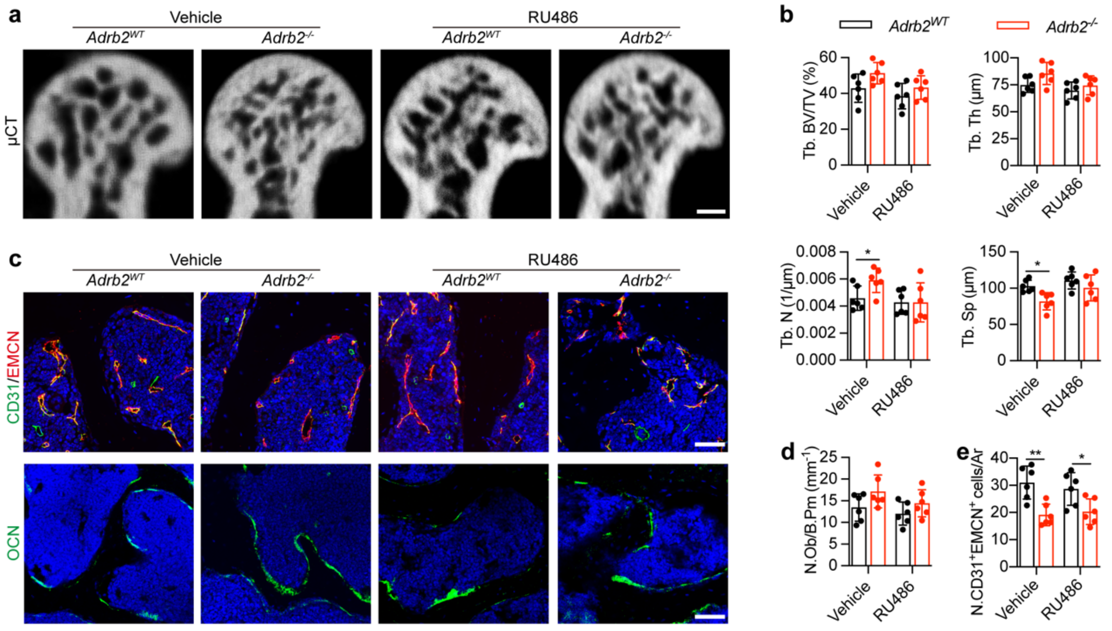


**Fig. S12** Effects of Adrb2 knockout on the femoral heads of mice following RU486 treatment in PVN. **a-b** µCT reconstruction images and quantitative analysis of Tb. BV/TV, Tb. Th, Tb. N and Tb. Sp of femoral heads. Scale bar: 1 mm. **c** Representative images of immunofluorescence staining of OCN (green) and co-staining of CD31 (green) with EMCN (red) **(d-e)** and quantitative analysis

of the number of OCN+ osteoblasts and CD31+EMCN+ cells in the femoral heads. Scale bar: 50 μm. All data were presented as means ± SD, $n = 6$ per group; $*P < 0.05$. $**P < 0.01$. $***P < 0.001$. Statistical significance was determined by two-way ANOVA with Bonferroni post hoc test.

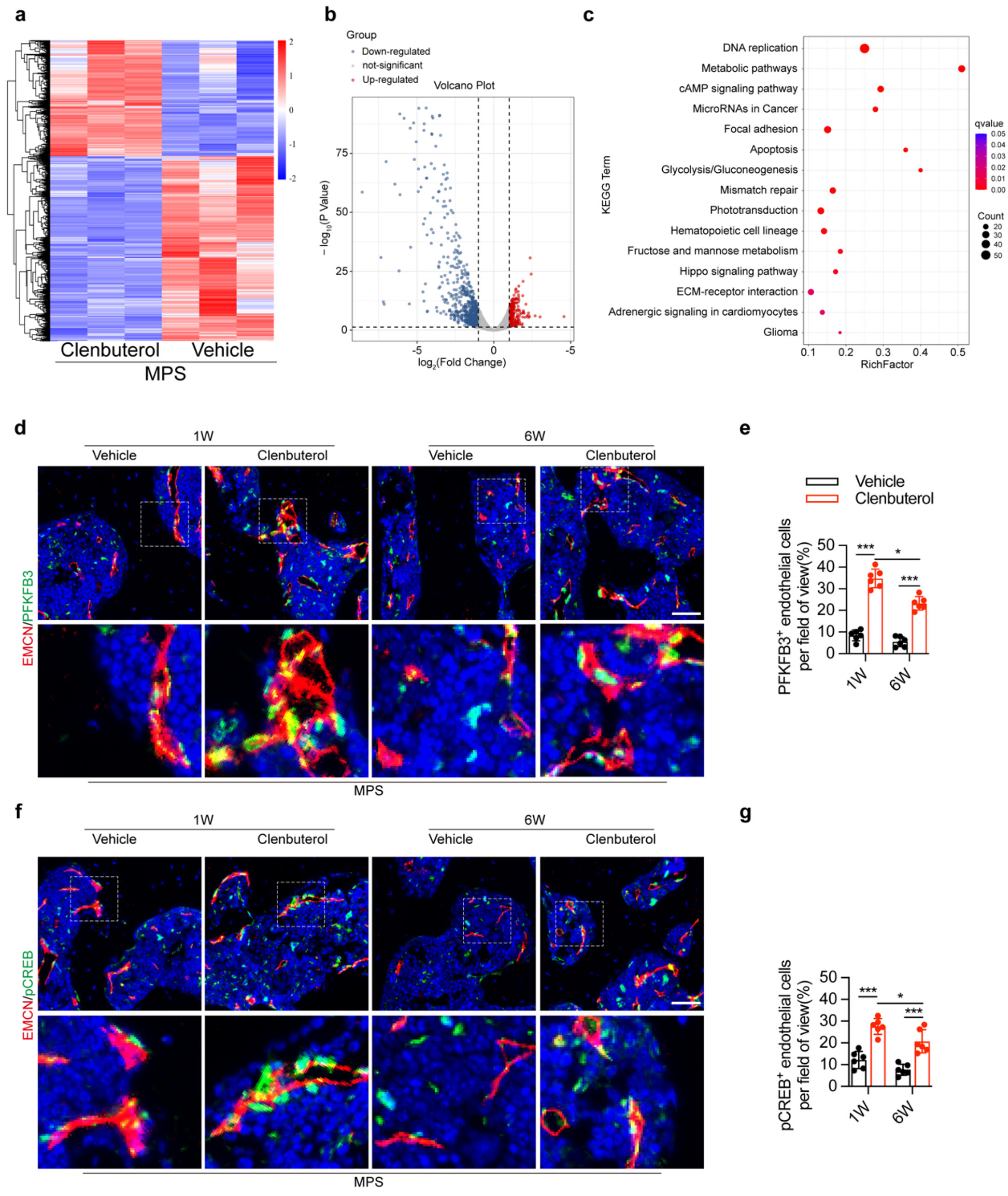


**Fig. S13** Sympathetic nerves stimulate glycolysis pathway and cAMP signaling in femoral head ECs. **a** Heat maps of the expression profiles of gene for the sorted femoral head ECs altered in MPS-treated mice receiving vehicle or clenbuterol treatment. **b** Volcano plots of the differentially expressed gene for the sorted femoral head ECs from MPS-treated mice receiving vehicle or clenbuterol treatment. **c** The enriched pathway for the sorted femoral head ECs from mice under different treatments as indicated through KEGG analysis. **d-e** Representative immunofluorescence co-staining of EMCN (red) and PFKFB3 (green) and quantification of PFKFB3+ endothelial cells for the femoral heads from 1 and 6 weeks after the first MPS treatment in vivo. Scale bar: 50 μm. **f-g** Representative immunofluorescence co-staining of EMCN (red) and pCREB (green) and

quantification of pCREB$^+$ endothelial cells for the femoral heads from 1 and 6 weeks after the first MPS treatment in vivo. Scale bar: 50 μm. All data were presented as means ± SD, $n$ = 6 per group; *$P$ < 0.05. **$P$ < 0.01. ***$P$ < 0.001. Statistical significance was determined by two-way ANOVA with Bonferroni post hoc test.

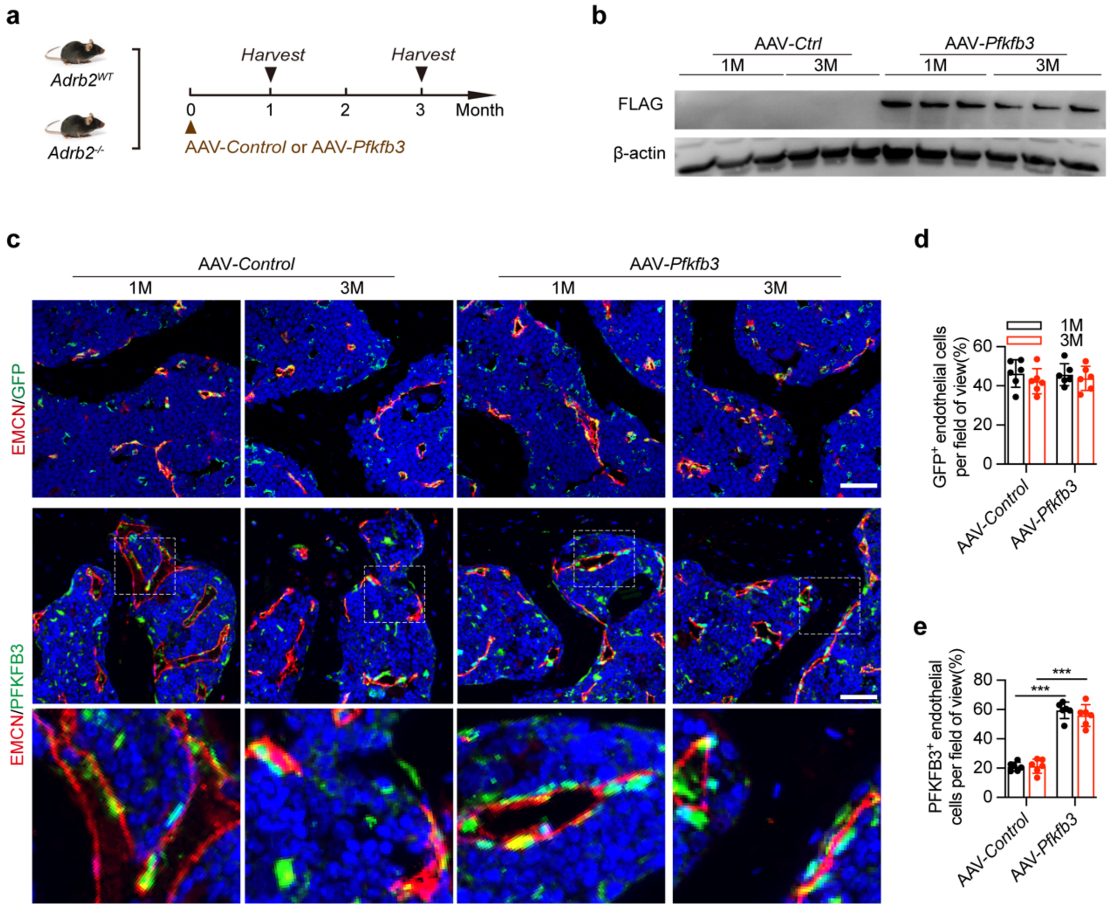


**Fig. S14** Identification of adeno-associated viral transfection for endothelial Pfkfb3 in the femoral heads of mice. **a** Schematic diagram showing the identification of adeno-associated viral (AAV) transfection for endothelial Pfkfb3 in mice; mice were harvested following transfection for 1 and 3 months. **b** Representative images of WB of FLAG expression for the femoral heads from 1 and 3 month after AAV transfection in vivo. **c-e** Representative immunofluorescence co-staining of EMCN (red) and GFP (green) and quantification of GFP$^+$ and PFKFB3$^+$ endothelial cells for the femoral heads from 1 and 3 month after AAV transfection in vivo. Scale bar: 50 μm. All data were presented as means ± SD, $n$ = 6 per group; *$P$ < 0.05. **$P$ < 0.01. Statistical significance was determined by two-way ANOVA with Bonferroni post hoc test.

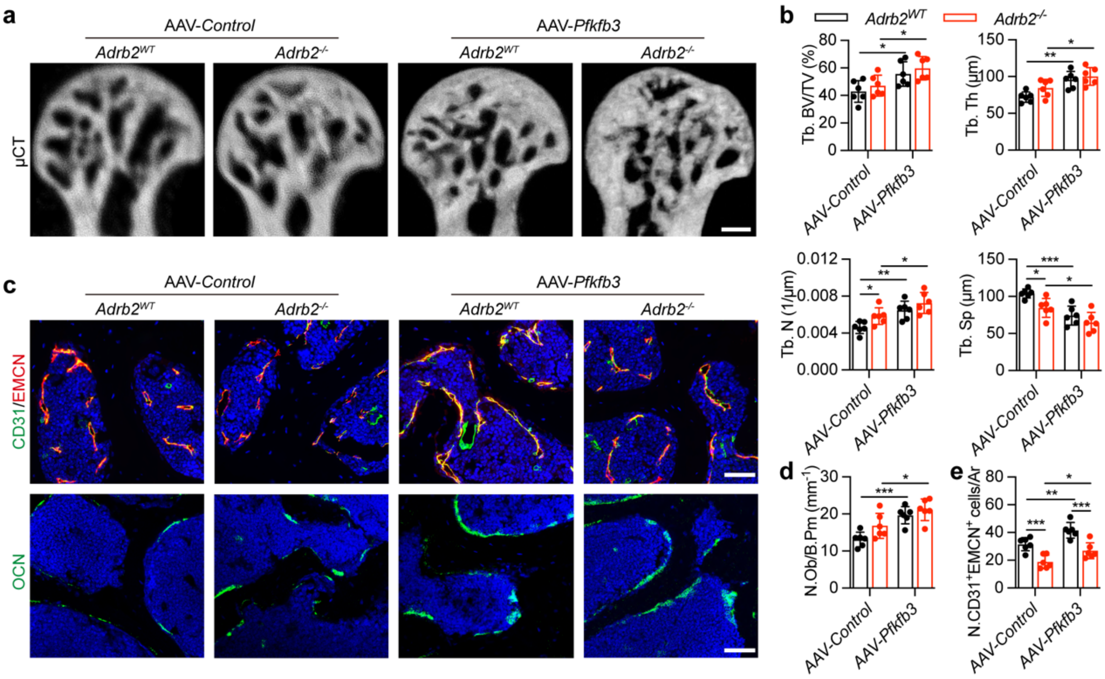


**Fig. S15** Effects of overexpression of endothelial Pfkfb3 on the femoral heads of *Adrb2*$^{WT}$ and *Adrb2*$^{-/-}$ mice. **a-b** μCT reconstruction images and quantitative analysis of Tb. BV/TV, Tb. Th, Tb. N and Tb. Sp of femoral heads. Scale bar: 1 mm. **c** Representative images of immunofluorescence staining of OCN (green) and co-staining of CD31 (green) with EMCN (red) **(d-e)** and quantitative analysis of the number of OCN$^+$ osteoblasts and CD31$^+$EMCN$^+$ cells in the femoral heads. Scale bar: 50 μm. All data were presented as means ± SD, $n$ = 6 per group; *$P$ < 0.05. **$P$ < 0.01. ***$P$ < 0.001. Statistical significance was determined by two-way ANOVA with Bonferroni post hoc test.

**Table S1. Clinical subjects' information**

| Laboratory number | Age | Sex | Direction | Imaging feature | ARCO stage | Duration of steroid(month) |
|---|---|---|---|---|---|---|
| 1 | 36 | M | left | X-ray and MRI normal | Ctrl(Healthy) | 0 |
| 2 | 42 | M | left | X-ray and MRI normal | Ctrl(Healthy) | 0 |
| 3 | 31 | M | left | X-ray and MRI normal | Ctrl(Healthy) | 0 |
| 4 | 29 | M | left | X-ray and MRI normal | Ctrl(Healthy) | 0 |
| 5 | 57 | M | left | X-ray and MRI normal | Ctrl(Healthy) | 0 |
| 6 | 32 | M | left | X-ray and MRI normal | Ctrl(Healthy) | 0 |
| 7 | 48 | M | left | X-ray normal; MRI abnormal | 1 | 2.6 |
| 8 | 40 | M | left | X-ray normal; MRI abnormal | 1 | 1.4 |
| 9 | 46 | M | left | X-ray normal; MRI abnormal | 1 | 7.3 |
| 10 | 39 | M | left | X-ray normal; MRI abnormal | 1 | 2.9 |
| 11 | 48 | M | left | X-ray normal; MRI abnormal | 1 | 4.2 |
| 12 | 50 | M | left | X-ray normal; MRI abnormal | 1 | 5.8 |
| 13 | 46 | M | left | Cystic changes; focal bone mineral density reduction | 2 | 3.2 |
| 14 | 24 | M | left | Cystic changes; focal bone mineral density reduction | 2 | 6.4 |
| 15 | 45 | M | left | Cystic changes; focal bone mineral density reduction | 2 | 8.3 |
| 16 | 38 | M | left | Cystic changes; focal bone mineral density reduction | 2 | 1.0 |
| 17 | 32 | M | left | Cystic changes; focal bone mineral density reduction | 2 | 2.7 |
| 18 | 55 | M | left | Cystic changes; focal bone mineral density reduction | 2 | 1.8 |
| 19 | 42 | M | left | Cystic changes; focal bone mineral density reduction | 2 | 2.6 |
| 20 | 25 | M | left | Cystic changes; focal bone mineral density reduction | 2 | 5.9 |
| 21 | 21 | M | left | subchondral fracture | 3 | 3.5 |
| 22 | 59 | M | left | subchondral fracture | 3 | 8.3 |
| 23 | 57 | M | left | subchondral fracture | 3 | 2.7 |
| 24 | 42 | M | left | subchondral fracture | 3 | 6.4 |
| 25 | 36 | M | left | subchondral fracture | 3 | 3.1 |
| 26 | 40 | M | left | subchondral fracture | 3 | 2.8 |
| 27 | 28 | M | left | subchondral fracture | 3 | 7.4 |
| 28 | 53 | M | left | subchondral fracture | 3 | 2.2 |
| 29 | 60 | M | left | subchondral fracture | 3 | 6.9 |
| 30 | 39 | M | left | subchondral fracture | 3 | 2.7 |
| 31 | 41 | M | left | severe femoral head depression; osteoarthritis | 4 | 8.4 |
| 32 | 58 | M | left | severe femoral head depression; osteoarthritis | 4 | 9.7 |
| 33 | 25 | M | left | severe femoral head depression; osteoarthritis | 4 | 7.3 |
| 34 | 30 | M | left | severe femoral head depression; osteoarthritis | 4 | 6.3 |
| 35 | 54 | M | left | severe femoral head depression; osteoarthritis | 4 | 11.6 |
| 36 | 51 | M | left | severe femoral head depression; osteoarthritis | 4 | 8.5 |

**Table S1. Clinical subjects' information**

| Laboratory number | Age | Sex | Direction | Imaging feature | ARCO stage | Duration of steroid(month) |
|---|---|---|---|---|---|---|
| 37 | 77 | M | left | Femoral neck fracture | Ctrl | 0 |
| 38 | 82 | M | left | Femoral neck fracture | Ctrl | 0 |
| 39 | 86 | M | left | Femoral neck fracture | Ctrl | 0 |
| 40 | 69 | M | left | Femoral neck fracture | Ctrl | 0 |
| 41 | 78 | M | left | Femoral neck fracture | Ctrl | 0 |
| 42 | 84 | M | left | Femoral neck fracture | Ctrl | 0 |

**Table S2. Association research circulation osseous (ARCO) international classification of osteonecrosis (2019 version)[78].**

| ARCO stage | Image findings | Representative images | | Description |
|---|---|---|---|---|
| | | X-ray | MRI | |
| 1 | X-ray normal;<br>MRI abnormal | 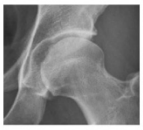 | 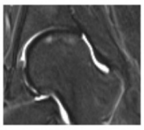 | A band lesion of low signal intensity around the necrotic area is seen on MRI;<br>No changes are seen on plain radiographs. |
| 2 | X-ray abnormal;<br>MRI abnormal | 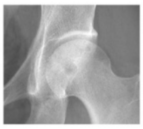 | 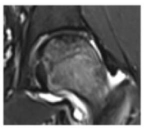 | Osteosclerosis;<br>Focal osteoporosis or cystic changes are seen in the femoral head on plain radiographs;<br>Still there is no evidence of subchondral frature, fracture in the necrotic portion or flattening of the femoral head. |
| 3 | Subchondral fracture | 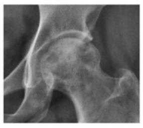 | 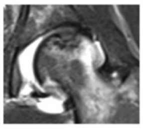 | Subchondral fracture, fracture in the necrotic portion and/or flattening of the femoral head is seen on plain radiograghy. |
| 4 | Severe femoral head depression;<br>Osteoarthritis | 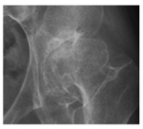 | 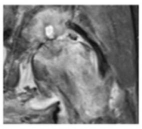 | Osteoarthritis of the hip joint with joint space narrowing. |

**Table S3. Primer sequences for qRT-PCR**

| Gene | Forward (5'-3') | Reverse (5'-3') |
|---|---|---|
| *Crh* | CCTCAGCCGGTTCTGATCC | GCGGAAAAAGTTAGCCGCAG |
| *Vegfa* | CGTCCAACTTCTGGGCTCTT | CAGCTCCGATCGGTTTGTCT |
| *Vegfc* | CCCAAACCAGTCACAATCAG | GGTAATGTTGCTGGCAGAGA |
| *Tgfa* | CCAGCATGTGTTGGTCTGAAG | AGGGGTGTTGTCCCAAGATG |
| *Tgfb2* | CTCCATAGATATGGGCATGC | AATGTGCAGGATAATTGCTGC |
| *Bmp2* | GCCTGCACCCTGTTCTCTGA | ATGTTCAAACACATATCCCTGGAA |
| *Noggin* | GGGGCGAAGTAGCCATAAAG | GGGGCGAAGTAGCCATAAAG |
| *Ptn* | TGGAGAATGGCAGTGGAGTGTGT | TGGTACTTGCACTCAGCTCCAAACT |
| *Adra1a* | GCACAGAGAAGCGGATTT | CGTTGAGGAACAGAGCATT |
| *Adra1b* | CCCTTCTACGCCCTCTTT | TCTTGGTGGTCCTCTTGG |
| *Adra1d* | GCCTTGGTGGTATCTGTGG | GGGGAGGTAGAAGGAGCAT |
| *Adra2a* | CTTCCGTTCTCCCCTCCT | ATTTCCCCTCCTGGGTGT |
| *Adra2b* | CCAGGGTGGGTTTATCAG | CCAAGGACCAAGCTATTCA |
| *Adra2c* | GGTGACGCAAGCGGTAGA | GAGACGAGAGGCGGGAAG |
| *Adrb1* | CAGAAGGCGCTCAAGACA | CCCAGCCAGTTGAAGAAGA |
| *Adrb2* | TGCGCTCACCTGCTAAC | TCGTCCCGTTCCTGAGT |
| *Adrb3* | ACAAGCGGGTGTCTCCA | GAGGGGCGTCCTGTCTT |
| *Pfkfb3* | CAGGATCTTGTCCAACGCCT | AGTAGGCAGCGTAGAACAGC |
| *Pfkl* | CATGAATGCAGCTGTGCGCTCC | CCAGCCCACTTCTTGCACCTGA |
| *Pfkp* | ACAGACACGTGCGACCGCAT | AGTGCACGACGTTGGACTGCA |
| *Eno1* | TGCGTCCACTGGCATCTAC | CAGAGCAGGCGCAATAGTTTTA |
| *Hk1* | AACGGCCTCCGTCAAGATG | GCCGAGATCCAGTGCAATG |
