# Supplementary material for: Inhibition of sympathetic tone via hypothalamic descending pathway propagates glucocorticoid-induced endothelial impairment and osteonecrosis of the femoral head
Source: Bone Res. 2024 Nov 8;12:64. doi: 10.1038/s41413-024-00371-3 (PMC11549335; doi:10.1038/s41413-024-00371-3)
Supplement: Supplementary file 2 — p-value table for two-way ANOVA analysis [file 41413_2024_371_MOESM2_ESM.pdf]

Fig. 3b Serum Norepinephrine (ng/ml)

MPS+Vehicle - MPS+RU486	p value	Vehicle - 6-OHDA	p value
Vehicle	0.003	MPS+Vehicle	<0.001
6-OHDA	0.924	MPS+RU486	<0.001

Fig. 3b Bone marrow Norepinephrine (pg/ml)

MPS+Vehicle - MPS+RU486	p value	Vehicle - 6-OHDA	p value
Vehicle	<0.001	MPS+Vehicle	<0.001
6-OHDA	0.962	MPS+RU486	<0.001

Fig. 3e Tb. BV/TV (%)

MPS+Vehicle - MPS+RU486	p value	Vehicle - 6-OHDA	p value
Vehicle	<0.001	MPS+Vehicle	0.005
6-OHDA	0.836	MPS+RU486	<0.001

Fig. 3e Tb. Sp (μm)

MPS+Vehicle - MPS+RU486	p value	Vehicle - 6-OHDA	p value
Vehicle	<0.001	MPS+Vehicle	0.003
6-OHDA	0.633	MPS+RU486	<0.001

Fig. 3e Tb. N (1/μm)

MPS+Vehicle - MPS+RU486	p value	Vehicle - 6-OHDA	p value
Vehicle	<0.001	MPS+Vehicle	0.021
6-OHDA	0.137	MPS+RU486	<0.001

Fig. 3e Tb. Th (μm)

MPS+Vehicle - MPS+RU486	p value	Vehicle - 6-OHDA	p value
Vehicle	<0.001	MPS+Vehicle	<0.001
6-OHDA	0.507	MPS+RU486	<0.001

Fig. 3h N. OB/B.Pm (mm-1)

MPS+Vehicle - MPS+RU486	p value	Vehicle - 6-OHDA	p value
Vehicle	<0.001	MPS+Vehicle	0.001
6-OHDA	0.947	MPS+RU486	<0.001

Fig. 3i N. CD31+EMCN+ cells/Ar

MPS+Vehicle - MPS+RU486	p value	Vehicle - 6-OHDA	p value
Vehicle	<0.001	MPS+Vehicle	0.001
6-OHDA	0.926	MPS+RU486	<0.001

Fig. 3k *Vegfa*

Control - Sympathetic neuron	p value	$EC^{Vehicle}$ - EC^{MPS}	p value
$EC^{Vehicle}$	<0.001	Control	<0.001
EC^{MPS}	<0.001	Sympathetic neuron	<0.001

Fig. 3k *Vegfc*

Control - Sympathetic neuron	p value	$EC^{Vehicle}$ - EC^{MPS}	p value
$EC^{Vehicle}$	<0.001	Control	<0.001
EC^{MPS}	<0.001	Sympathetic neuron	<0.001

Fig. 3k *Tgfa*

Control - Sympathetic neuron	p value	$EC^{Vehicle}$ - EC^{MPS}	p value
$EC^{Vehicle}$	<0.001	Control	<0.001
EC^{MPS}	<0.001	Sympathetic neuron	<0.001

Fig. 3k *Tgfb2*

Control - Sympathetic neuron	p value	$EC^{Vehicle}$ - EC^{MPS}	p value
$EC^{Vehicle}$	<0.001	Control	<0.001
EC^{MPS}	<0.001	Sympathetic neuron	<0.001

Fig. 3i *Bmp2*

Control - Sympathetic neuron	p value	$EC^{Vehicle}$ - EC^{MPS}	p value
$EC^{Vehicle}$	<0.001	Control	<0.001
EC^{MPS}	<0.001	Sympathetic neuron	<0.001

Fig. 3i *Noggin*

Control - Sympathetic neuron	p value	$EC^{Vehicle}$ - EC^{MPS}	p value
$EC^{Vehicle}$	0.482	Control	<0.001
EC^{MPS}	0.377	Sympathetic neuron	<0.001

Fig. 3i *Ptn*

Control - Sympathetic neuron	p value		p value
$EC^{Vehicle}$	<0.001	Control	<0.001
EC^{MPS}	<0.001	Sympathetic neuron	<0.001

Fig. 3n ARS^{+} area (%)

$EC^{Vehicle}$ - EC^{MPS}	p value	Control - Sympathetic neuron	p value
Control	<0.001	$EC^{Vehicle}$	<0.001
Sympathetic neuron	<0.001	EC^{MPS}	<0.001

Fig. 4l Tb. BV/TV (%)

MPS+Vehicle - MPS+6-OHDA	p value	Vehicle - Clenbuterol	p value
Vehicle	0.003	MPS+Vehicle	<0.001
Clenbuterol	0.135	MPS+6-OHDA	<0.001

Fig. 4l Tb. Sp (μm)

MPS+Vehicle - MPS+6-OHDA	p value	Vehicle - Clenbuterol	p value
Vehicle	<0.001	MPS+Vehicle	<0.001
Clenbuterol	0.127	MPS+6-OHDA	<0.001

Fig. 4l Tb. N (1/μm)

MPS+Vehicle - MPS+6-OHDA	p value	Vehicle - Clenbuterol	p value
Vehicle	0.008	MPS+Vehicle	<0.001
Clenbuterol	0.152	MPS+6-OHDA	<0.001

Fig. 4l Tb. Th (μm)

MPS+Vehicle - MPS+6-OHDA	p value	Vehicle - Clenbuterol	p value
Vehicle	0.002	MPS+Vehicle	<0.001
Clenbuterol	0.037	MPS+6-OHDA	<0.001

Fig. 4o N. OB/B.Pm (mm-1)

MPS+Vehicle - MPS+6-OHDA	p value	Vehicle - Clenbuterol	p value
Vehicle	0.032	MPS+Vehicle	<0.001
Clenbuterol	0.135	MPS+6-OHDA	<0.001

Fig. 4p N. CD31+EMCN+ cells/Ar

MPS+Vehicle - MPS+6-OHDA	p value	Vehicle - Clenbuterol	p value
Vehicle	0.026	MPS+Vehicle	<0.001
Clenbuterol	0.064	MPS+6-OHDA	<0.001

Fig. 5f Tb. BV/TV (%)

Adrb2WT+MPS - Adrb2-/-+MPS	p value	Vehicle - RU486	p value
Vehicle	0.024	*Adrb2*^{WT}+MPS	0.012
RU486	<0.001	*Adrb2*$^{-/-}$+MPS	0.122

Fig. 5f Tb. Sp (μm)

Adrb2WT+MPS - Adrb2-/-+MPS	p value	Vehicle - RU486	p value
Vehicle	0.044	*Adrb2*^{WT}+MPS	0.005
RU486	0.002	*Adrb2*$^{-/-}$+MPS	0.926

Fig. 5f Tb. N (1/μm)

Adrb2WT+MPS - Adrb2-/-+MPS	p value	Vehicle - 6-OHDA	p value
Vehicle	0.002	*Adrb2*^{WT}+MPS	0.009
RU486	0.005	*Adrb2*$^{-/-}$+MPS	0.682

Fig. 5f Tb. Th (μm)

Adrb2WT+MPS - Adrb2-/-+MPS	p value	Vehicle - 6-OHDA	p value
Vehicle	0.003	*Adrb2*^{WT}+MPS	0.008
RU486	0.002	*Adrb2*$^{-/-}$+MPS	0.784

Fig. 5i N. OB/B.Pm (mm-1)

Adrb2WT+MPS - Adrb2-/-+MPS	p value	Vehicle - 6-OHDA	p value
Vehicle	0.035	*Adrb2*^{WT}+MPS	0.002
RU486	<0.001	*Adrb2*$^{-/-}$+MPS	0.873

Fig. 5j N. CD31+EMCN+ cells/Ar

Adrb2WT+MPS - Adrb2-/-+MPS	p value	Vehicle - 6-OHDA	p value
Vehicle	0.008	*Adrb2*^{WT}+MPS	0.003
RU486	0.001	*Adrb2*$^{-/-}$+MPS	0.169

Fig. 6l Total loops

Vehicle	p value	MPS	p value
Vehicle vs. NE	<0.001	Vehicle vs. NE	<0.001
Vehicle vs. E	0.026	Vehicle vs. E	0.074
Vehicle vs. NPY	<0.001	Vehicle vs. NPY	0.002
Vehicle vs. DA	0.008	Vehicle vs. DA	0.871
NE vs. NPY	<0.001	NE vs. NPY	0.001
NE vs. E	<0.001	NE vs. E	<0.001
NE vs. DA	<0.001	NE vs. DA	<0.001

Vehicle - MPS	p value
Vehicle	0.002

Fig. 6l Total tube length (px)

Vehicle	p value	MPS	p value
Vehicle vs. NE	<0.001	Vehicle vs. NE	<0.001
Vehicle vs. E	0.029	Vehicle vs. E	0.046
Vehicle vs. NPY	<0.001	Vehicle vs. NPY	<0.001
Vehicle vs. DA	0.003	Vehicle vs. DA	0.871
NE vs. NPY	<0.001	NE vs. NPY	0.012
NE vs. E	<0.001	NE vs. E	<0.001
NE vs. DA	<0.001	NE vs. DA	<0.001

Vehicle - MPS	p value
Vehicle	<0.001

Fig. 7b Relative intensity of PFKFB3 protein expression

MPS+Vehicle - MPS+NE	p value
Control	<0.001
si-Adrb2	0.847
666-15	0.322

MPS+Vehicle	p value	MPS+NE	p value
Control vs. si-Adrb2	0.008	Control vs. si-Adrb2	<0.001
Control vs. 666-15	0.002	Control vs. 666-15	<0.001
si-Adrb2 vs. 666-15	0.921	si-Adrb2 vs. 666-15	0.963

Fig. 7c Relative intensity of pCREB protein expression

MPS+Vehicle - MPS+NE	p value
Control	<0.001
si-Adrb2	0.922
666-15	0.974

MPS+Vehicle	p value	MPS+NE	p value
Control vs. si-Adrb2	0.009	Control vs. si-Adrb2	<0.001
Control vs. 666-15	0.01	Control vs. 666-15	<0.001
si-Adrb2 vs. 666-15	0.967	si-Adrb2 vs. 666-15	0.892

Fig. 7f Glucose uptake (fold change of control)

MPS+Vehicle - MPS+NE	p value
Control	<0.001
si-Adrb2	0.982
666-15	0.954

MPS+Vehicle	p value	MPS+NE	p value
Control vs. si-Adrb2	0.925	Control vs. si-Adrb2	<0.001
Control vs. 666-15	0.593	Control vs. 666-15	<0.001
si-Adrb2 vs. 666-15	0.921	si-Adrb2 vs. 666-15	0.916

Fig. 7g Glucose-6-phosphate (pmol/mg protein)

MPS+Vehicle - MPS+NE	p value
Control	<0.001
si-Adrb2	0.272
666-15	0.306

MPS+Vehicle	p value	MPS+NE	p value
Control vs. si-Adrb2	0.753	Control vs. si-Adrb2	0.002
Control vs. 666-15	0.608	Control vs. 666-15	0.002
si-Adrb2 vs. 666-15	0.946	si-Adrb2 vs. 666-15	0.97

Fig. 7e ECAR (mpH/min) Glycolysis

MPS+Vehicle - MPS+NE	p value
Control	<0.001
si-Adrb2	0.673
666-15	0.838

MPS+Vehicle	p value	MPS+NE	p value
Control vs. si-Adrb2	0.728	Control vs. si-Adrb2	0.004
Control vs. 666-15	0.681	Control vs. 666-15	0.003
si-Adrb2 vs. 666-15	0.921	si-Adrb2 vs. 666-15	0.896

Fig. 7e ECAR (mpH/min) Glycolysis capacity

MPS+Vehicle - MPS+NE	p value
Control	<0.001
si-Adrb2	0.953
666-15	0.75

MPS+Vehicle	p value	MPS+NE	p value
Control vs. si-Adrb2	0.575	Control vs. si-Adrb2	<0.001
Control vs. 666-15	0.539	Control vs. 666-15	<0.001
si-Adrb2 vs. 666-15	0.962	si-Adrb2 vs. 666-15	0.917

Fig. 7e ECAR (mpH/min) Glycolysis reserve

MPS+Vehicle - MPS+NE	p value
Control	<0.001
si-Adrb2	0.126
666-15	0.374

MPS+Vehicle	p value	MPS+NE	p value
Control vs. si-Adrb2	0.776	Control vs. si-Adrb2	<0.001
Control vs. 666-15	0.931	Control vs. 666-15	<0.001
si-Adrb2 vs. 666-15	0.948	si-Adrb2 vs. 666-15	0.962

Fig. 7h Extracellular lactate level (mg/10^6 cells)

MPS+Vehicle - MPS+NE	p value
Control	<0.001
si-Adrb2	0.938
666-15	0.951

MPS+Vehicle	p value	MPS+NE	p value
Control vs. si-Adrb2	0.946	Control vs. si-Adrb2	<0.001
Control vs. 666-15	0.939	Control vs. 666-15	<0.001
si-Adrb2 vs. 666-15	0.906	si-Adrb2 vs. 666-15	0.935

Fig. 7i Intracellular lactate level (mg/10^6 cells)

MPS+Vehicle - MPS+NE	p value
Control	0.002
si-Adrb2	0.847
666-15	0.322

MPS+Vehicle	p value	MPS+NE	p value
Control vs. si-Adrb2	0.196	Control vs. si-Adrb2	0.003
Control vs. 666-15	0.449	Control vs. 666-15	0.003
si-Adrb2 vs. 666-15	0.913	si-Adrb2 vs. 666-15	0.847

Fig. 7j Pyruvate (nmol/mg protein)

MPS+Vehicle - MPS+NE	p value
Control	<0.001
si-Adrb2	0.13
666-15	0.101

MPS+Vehicle	p value	MPS+NE	p value
Control vs. si-Adrb2	0.913	Control vs. si-Adrb2	<0.001
Control vs. 666-15	0.476	Control vs. 666-15	<0.001
si-Adrb2 vs. 666-15	0.958	si-Adrb2 vs. 666-15	0.963

Fig. 7k *Vegfa* relative gene expression

MPS+NE - MPS+NE+666-15	p value
Control	<0.001
db-cAMP	<0.001
Ad-Pfkfb3	<0.001

MPS+NE	p value	MPS+NE+666-15	p value
Control vs. db-cAMP	<0.001	Control vs. db-cAMP	0.891
Control vs. Ad-Pfkfb3	<0.001	Control vs. Ad-Pfkfb3	<0.001
db-cAMP vs. Ad-Pfkfb3	0.185	db-cAMP vs. Ad-Pfkfb3	<0.001

Fig. 7k *Vegfc* relative gene expression

MPS+NE - MPS+NE+666-15	p value
Control	<0.001
db-cAMP	<0.001
Ad-Pfkfb3	0.064

MPS+NE	p value	MPS+NE+666-15	p value
Control vs. db-cAMP	0.027	Control vs. db-cAMP	0.828
Control vs. Ad-Pfkfb3	<0.001	Control vs. Ad-Pfkfb3	<0.001
db-cAMP vs. Ad-Pfkfb3	0.731	db-cAMP vs. Ad-Pfkfb3	<0.001

Fig. 7k *Tgfa* relative gene expression

MPS+NE - MPS+NE+666-15	p value
Control	<0.001
db-cAMP	<0.001
Ad-Pfkfb3	<0.001

MPS+NE	p value	MPS+NE+666-15	p value
Control vs. db-cAMP	<0.001	Control vs. db-cAMP	0.352
Control vs. Ad-Pfkfb3	<0.001	Control vs. Ad-Pfkfb3	<0.001
db-cAMP vs. Ad-Pfkfb3	0.079	db-cAMP vs. Ad-Pfkfb3	<0.001

Fig. 7k *Tgfb2* relative gene expression

MPS+NE - MPS+NE+666-15	p value
Control	<0.001
db-cAMP	<0.001
Ad-Pfkfb3	<0.001

MPS+NE	p value	MPS+NE+666-15	p value
Control vs. db-cAMP	<0.001	Control vs. db-cAMP	0.81
Control vs. Ad-Pfkfb3	<0.001	Control vs. Ad-Pfkfb3	<0.001
db-cAMP vs. Ad-Pfkfb3	0.114	db-cAMP vs. Ad-Pfkfb3	<0.001

Fig. 7l *Bmp2* relative gene expression

MPS+NE - MPS+NE+666-15	p value
Control	<0.001
db-cAMP	<0.001
Ad-Pfkfb3	<0.001

MPS+NE	p value	MPS+NE+666-15	p value
Control vs. db-cAMP	<0.001	Control vs. db-cAMP	0.827
Control vs. Ad-Pfkfb3	<0.001	Control vs. Ad-Pfkfb3	<0.001
db-cAMP vs. Ad-Pfkfb3	0.032	db-cAMP vs. Ad-Pfkfb3	<0.001

Fig. 7l *Noggin* relative gene expression

MPS+NE - MPS+NE+666-15	p value
Control	0.941
db-cAMP	0.813
Ad-Pfkfb3	0.989

MPS+NE	p value	MPS+NE+666-15	p value
Control vs. db-cAMP	0.597	Control vs. db-cAMP	0.813
Control vs. Ad-Pfkfb3	0.014	Control vs. Ad-Pfkfb3	0.006
db-cAMP vs. Ad-Pfkfb3	0.921	db-cAMP vs. Ad-Pfkfb3	0.108

Fig. 7l *Ptn* relative gene expression

MPS+NE - MPS+NE+666-15	p value
Control	<0.001
db-cAMP	<0.001
Ad-Pfkfb3	<0.001

MPS+NE	p value	MPS+NE+666-15	p value
Control vs. db-cAMP	<0.001	Control vs. db-cAMP	0.295
Control vs. Ad-Pfkfb3	<0.001	Control vs. Ad-Pfkfb3	<0.001
db-cAMP vs. Ad-Pfkfb3	0.328	db-cAMP vs. Ad-Pfkfb3	<0.001

Fig. 7n Total loops

MPS+NE - MPS+NE+666-15	p value
Control	<0.001
db-cAMP	<0.001
Ad-Pfkfb3	<0.001

MPS+NE	p value	MPS+NE+666-15	p value
Control vs. db-cAMP	<0.001	Control vs. db-cAMP	0.953
Control vs. Ad-Pfkfb3	<0.001	Control vs. Ad-Pfkfb3	<0.001
db-cAMP vs. Ad-Pfkfb3	0.981	db-cAMP vs. Ad-Pfkfb3	<0.001

Fig. 7n Total tube length (px)

MPS+NE - MPS+NE+666-15	p value
Control	<0.001
db-cAMP	<0.001
Ad-Pfkfb3	<0.001

MPS+NE	p value	MPS+NE+666-15	p value
Control vs. db-cAMP	<0.001	Control vs. db-cAMP	0.956
Control vs. Ad-Pfkfb3	<0.001	Control vs. Ad-Pfkfb3	<0.001
db-cAMP vs. Ad-Pfkfb3	0.974	db-cAMP vs. Ad-Pfkfb3	<0.001

Fig. 7p ARS^+ area (%)

MPS+NE - MPS+NE+666-15	p value
Control	0.001
db-cAMP	<0.001
Ad-Pfkfb3	<0.001

MPS+NE	p value	MPS+NE+666-15	p value
Control vs. db-cAMP	<0.001	Control vs. db-cAMP	0.952
Control vs. Ad-Pfkfb3	<0.001	Control vs. Ad-Pfkfb3	<0.001
db-cAMP vs. Ad-Pfkfb3	0.928	db-cAMP vs. Ad-Pfkfb3	<0.001

Fig.8c Annexin-V$^+$ cells (%)

Vehicle	p value	MPS	p value
Adrb2WT AAV-Control vs. Adrb2-/- AAV-Control	0.003	Adrb2WT AAV-Control vs. Adrb2-/- AAV-Control	<0.001
Adrb2WT AAV-Control vs. Adrb2WT AAV-Pfkfb3	0.769	Adrb2WT AAV-Control vs. Adrb2WT AAV-Pfkfb3	<0.001
Adrb2-/- AAV-Control vs. Adrb2-/- AAV-Pfkfb3	0.024	Adrb2-/- AAV-Control vs. Adrb2-/- AAV-Pfkfb3	<0.001
Adrb2WT AAV-Pfkfb3 vs. Adrb2-/- AAV-Pfkfb3	0.352	Adrb2WT AAV-Pfkfb3 vs. Adrb2-/- AAV-Pfkfb3	<0.001

Vehicle - MPS	p value
Adrb2WT AAV-Control	<0.001
Adrb2-/- AAV-Control	<0.001
Adrb2WT AAV-Pfkfb3	<0.001
Adrb2-/- AAV-Pfkfb3	<0.001

Fig.8e Total loops

Vehicle	p value	MPS	p value
Adrb2WT AAV-Control vs. Adrb2-/- AAV-Control	<0.001	Adrb2WT AAV-Control vs. Adrb2-/- AAV-Control	0.021
Adrb2WT AAV-Control vs. Adrb2WT AAV-Pfkfb3	<0.001	Adrb2WT AAV-Control vs. Adrb2WT AAV-Pfkfb3	<0.001
Adrb2-/- AAV-Control vs. Adrb2-/- AAV-Pfkfb3	<0.001	Adrb2-/- AAV-Control vs. Adrb2-/- AAV-Pfkfb3	0.004
Adrb2WT AAV-Pfkfb3 vs. Adrb2-/- AAV-Pfkfb3	<0.001	Adrb2WT AAV-Pfkfb3 vs. Adrb2-/- AAV-Pfkfb3	0.001

Vehicle - MPS	p value
Adrb2WT AAV-Control	<0.001
Adrb2-/- AAV-Control	0.042
Adrb2WT AAV-Pfkfb3	<0.001
Adrb2-/- AAV-Pfkfb3	<0.001

Fig.8e Total tube length (px)

Vehicle	p value	MPS	p value
Adrb2WT AAV-Control vs. Adrb2-/- AAV-Control	<0.001	Adrb2WT AAV-Control vs. Adrb2-/- AAV-Control	0.001
Adrb2WT AAV-Control vs. Adrb2WT AAV-Pfkfb3	<0.001	Adrb2WT AAV-Control vs. Adrb2WT AAV-Pfkfb3	<0.001
Adrb2-/- AAV-Control vs. Adrb2-/- AAV-Pfkfb3	<0.001	Adrb2-/- AAV-Control vs. Adrb2-/- AAV-Pfkfb3	<0.001
Adrb2WT AAV-Pfkfb3 vs. Adrb2-/- AAV-Pfkfb3	<0.001	Adrb2WT AAV-Pfkfb3 vs. Adrb2-/- AAV-Pfkfb3	<0.001

Vehicle - MPS	p value
Adrb2WT AAV-Control	<0.001
Adrb2-/- AAV-Control	<0.001
Adrb2WT AAV-Pfkfb3	<0.001
Adrb2-/- AAV-Pfkfb3	<0.001

Fig.8h c-Casp3+ endothelial cells per field of view (%)

Vehicle	p value	MPS	p value
Adrb2WT AAV-Control vs. Adrb2-/- AAV-Control	0.043	Adrb2WT AAV-Control vs. Adrb2-/- AAV-Control	<0.001
Adrb2WT AAV-Control vs. Adrb2WT AAV-Pfkfb3	0.697	Adrb2WT AAV-Control vs. Adrb2WT AAV-Pfkfb3	<0.001
Adrb2-/- AAV-Control vs. Adrb2-/- AAV-Pfkfb3	0.031	Adrb2-/- AAV-Control vs. Adrb2-/- AAV-Pfkfb3	<0.001
Adrb2WT AAV-Pfkfb3 vs. Adrb2-/- AAV-Pfkfb3	0.728	Adrb2WT AAV-Pfkfb3 vs. Adrb2-/- AAV-Pfkfb3	<0.001

Vehicle - MPS	p value
Adrb2WT AAV-Control	<0.001
Adrb2-/- AAV-Control	<0.001
Adrb2WT AAV-Pfkfb3	0.168
Adrb2-/- AAV-Pfkfb3	<0.001

Fig. 8j Tb. BV/TV (%)

Adrb2WT+MPS - Adrb2-/-+MPS	p value	AAV-Control - AAV-Pfkfb3	p value
AAV-*Control*	0.003	*Adrb2*^{WT}+MPS	<0.001
AAV-*Pfkfb3*	<0.001	*Adrb2*$^{-/-}$+MPS	<0.001

Fig. 8j Tb. Sp (μm)

Adrb2WT+MPS - Adrb2-/-+MPS	p value	AAV-Control - AAV-Pfkfb3	p value
AAV-*Control*	<0.001	*Adrb2*^{WT}+MPS	<0.001
AAV-*Pfkfb3*	<0.001	*Adrb2*$^{-/-}$+MPS	<0.001

Fig. 8j Tb. N (1/μm)

Adrb2WT+MPS - Adrb2-/-+MPS	p value	AAV-Control - AAV-Pfkfb3	p value
AAV-*Control*	0.002	*Adrb2*^{WT}+MPS	0.002
AAV-*Pfkfb3*	0.039	*Adrb2*$^{-/-}$+MPS	<0.001

Fig. 8j Tb. Th (μm)

Adrb2WT+MPS - Adrb2-/-+MPS	p value	AAV-Control - AAV-Pfkfb3	p value
AAV-*Control*	0.002	*Adrb2*^{WT}+MPS	0.001
AAV-*Pfkfb3*	0.032	*Adrb2*$^{-/-}$+MPS	<0.001

Fig. 8m N. OB/B.Pm (mm-1)

Adrb2WT+MPS - Adrb2-/-+MPS	p value	AAV-Control - AAV-Pfkfb3	p value
AAV-*Control*	0.001	*Adrb2*^{WT}+MPS	<0.001
AAV-*Pfkfb3*	0.027	*Adrb2*$^{-/-}$+MPS	<0.001

Fig. 8n N. CD31+EMCN+ cells/Ar

Adrb2WT+MPS - Adrb2-/-+MPS	p value	AAV-Control - AAV-Pfkfb3	p value
AAV-*Control*	0.041	*Adrb2*^{WT}+MPS	<0.001
AAV-*Pfkfb3*	0.003	*Adrb2*$^{-/-}$+MPS	<0.001

SFig. 4c N. OC/B. Pm (mm^{-1})

Vehicle - RU486	p value	Vehicle - MPS	p value
Vehicle	0.491	Vehicle	<0.001
MPS	0.032	RU486	0.017

SFig. 4d Serum CTX concentration (ng/ml)

Vehicle - RU486	p value	Vehicle - MPS	p value
Vehicle	0.403	Vehicle	<0.001
MPS	0.021	RU486	0.046

SFig. 7a TH+ area per field of vew (%)

Vehicle - Vehicle+RU486	p value	Vehicle - 6-OHDA	p value
Vehicle	0.981	Vehicle	<0.001
6-OHDA	0.953	Vehicle+RU486	<0.001

SFig. 7a TH+ area per field of vew (%)

MPS+Vehicle - MPS+RU486	p value	Vehicle - 6-OHDA	p value
Vehicle	<0.001	MPS+Vehicle	0.029
6-OHDA	0.84	MPS+RU486	<0.001

SFig. 7b Serum Norepinephrine (ng/ml)

Vehicle - Vehicle+RU486	p value	Vehicle - 6-OHDA	p value
Vehicle	0.732	Vehicle	<0.001
6-OHDA	0.983	Vehicle+RU486	<0.001

SFig. 7b Bone marrow Norepinephrine (pg/ml)

Vehicle - Vehicle+RU486	p value	Vehicle - 6-OHDA	p value
Vehicle	0.775	Vehicle	<0.001
6-OHDA	0.942	Vehicle+RU486	<0.001

SFig. 7d Tb. BV/TV (%)

Vehicle - RU486	p value	Vehicle - 6-OHDA	p value
Vehicle	0.952	Vehicle	0.034
6-OHDA	0.996	RU486	0.048

SFig. 7d Tb. Sp (μm)

Vehicle - RU486	p value	Vehicle - 6-OHDA	p value
Vehicle	0.932	Vehicle	<0.001
6-OHDA	0.858	RU486	<0.001

SFig. 7d Tb. N (1/μm)

Vehicle - RU486	p value	Vehicle - 6-OHDA	p value
Vehicle	0.971	Vehicle	0.043
6-OHDA	0.869	RU486	0.227

SFig. 7d Tb. Th (μm)

Vehicle - RU486	p value	Vehicle - 6-OHDA	p value
Vehicle	0.128	Vehicle	<0.001
6-OHDA	0.982	RU486	<0.001

SFig. 7f N. OB/B.Pm (mm-1)

Vehicle - RU486	p value	Vehicle - 6-OHDA	p value
Vehicle	0.507	Vehicle	0.022
6-OHDA	0.921	RU486	0.136

SFig. 7g N. CD31+EMCN+ cells/Ar

Vehicle - RU486	p value	Vehicle - 6-OHDA	p value
Vehicle	0.669	Vehicle	0.003
6-OHDA	0.926	RU486	0.021

SFig. 11b Tb. BV/TV (%)

Vehicle - 6-OHDA	p value	Vehicle - Clenbuterol	p value
Vehicle	0.005	Vehicle	0.473
Clenbuterol	<0.001	6-OHDA	0.042

SFig. 11b Tb. Sp (μm)

Vehicle - 6-OHDA	p value	Vehicle - Clenbuterol	p value
Vehicle	0.043	Vehicle	0.058
Clenbuterol	<0.001	6-OHDA	<0.001

SFig. 11b Tb. N (1/μm)

Vehicle - 6-OHDA	p value	Vehicle - Clenbuterol	p value
Vehicle	0.031	Vehicle	0.276
Clenbuterol	0.008	6-OHDA	0.039

SFig. 11b Tb. Th (μm)

Vehicle - 6-OHDA	p value	Vehicle - Clenbuterol	p value
Vehicle	0.021	Vehicle	0.945
Clenbuterol	0.002	6-OHDA	0.032

SFig. 11d N. OB/B.Pm (mm-1)

Vehicle - 6-OHDA	p value	Vehicle - Clenbuterol	p value
Vehicle	0.042	Vehicle	0.522
Clenbuterol	0.037	6-OHDA	0.048

SFig. 11e N. CD31+EMCN+ cells/Ar

Vehicle - 6-OHDA	p value	Vehicle - Clenbuterol	p value
Vehicle	0.002	Vehicle	<0.001
Clenbuterol	0.025	6-OHDA	<0.001

SFig. 12b Tb. BV/TV (%)

Adrb2WT - Adrb2-/-	p value	Vehicle - RU486	p value
Vehicle	0.086	Adrb2WT	0.571
RU486	0.488	Adrb2-/-	0.124

SFig. 12b Tb. Sp (μm)

Adrb2WT - Adrb2-/-	p value	Vehicle - RU486	p value
Vehicle	0.017	Adrb2WT	0.598
RU486	0.373	Adrb2-/-	0.052

SFig. 12b Tb. N (1/μm)

Adrb2WT - Adrb2-/-	p value	Vehicle - RU486	p value
Vehicle	0.047	Adrb2WT	0.955
RU486	0.924	Adrb2-/-	0.069

SFig. 12b Tb. Th (μm)

Adrb2WT - Adrb2-/-	p value	Vehicle - RU486	p value
Vehicle	0.086	Adrb2WT	0.618
RU486	0.682	Adrb2-/-	0.074

SFig. 12d N. OB/B.Pm (mm-1)

Adrb2WT - Adrb2-/-	p value	Vehicle - RU486	p value
Vehicle	0.128	Adrb2WT	0.914
RU486	0.427	Adrb2-/-	0.317

SFig. 12e N. CD31+EMCN+ cells/Ar

Adrb2WT - Adrb2-/-	p value	Vehicle - RU486	p value
Vehicle	0.002	Adrb2WT	0.896
RU486	0.034	Adrb2-/-	0.938

SFig. 13e PFKFB3+ endothelial cells per field of view(%)

Vehicle - Clenbuterol	p value	1W - 6W	p value
MPS 1W	<0.001	Vehicle	0.124
MPS 6W	<0.001	Clenbuterol	0.019

SFig. 13g pCREB+ endothelial cells per field of view(%)

Vehicle - Clenbuterol	p value	Vehicle - RU486	p value
MPS 1W	<0.001	Vehicle	0.279
MPS 6W	<0.001	Clenbuterol	0.026

SFig. 14d GFP+ endothelial cells per field of view

1M - 3M	p value	AAV-Control - AAV-Pfkfb3	p value
AAV-Control	0.573	1M	0.702
AAV-Pfkfb3	0.824	3M	0.847

SFig. 14e PFKFB3+ endothelial cells per field of view

1M - 3M	p value	AAV-Control - AAV-Pfkfb3	p value
AAV-Control	0.816	1M	<0.001
AAV-Pfkfb3	0.589	3M	<0.001

SFig. 15b Tb. BV/TV (%)

Adrb2WT - Adrb2-/-	p value	AAV-Control - AAV-Pfkfb3	p value
AAV-Control	0.785	Adrb2WT	0.028
AAV-Pfkfb3	0.722	Adrb2-/-	0.036

SFig. 15b Tb. Sp (μm)

Adrb2WT - Adrb2-/-	p value	AAV-Control - AAV-Pfkfb3	p value
AAV-Control	0.027	Adrb2WT	<0.001
AAV-Pfkfb3	0.373	Adrb2-/-	0.021

SFig. 15b Tb. N (1/μm)

Adrb2WT - Adrb2-/-	p value	AAV-Control - AAV-Pfkfb3	p value
AAV-Control	0.02	Adrb2WT	0.005
AAV-Pfkfb3	0.329	Adrb2-/-	0.032

SFig. 15b Tb. Th (μm)

Adrb2WT - Adrb2-/-	p value	AAV-Control - AAV-Pfkfb3	p value
AAV-Control	0.11	Adrb2WT	0.002
AAV-Pfkfb3	0.897	Adrb2-/-	0.036

SFig. 15d N. OB/B.Pm (mm-1)

Adrb2WT - Adrb2-/-	p value	AAV-Control - AAV-Pfkfb3	p value
AAV-Control	0.063	Adrb2WT	<0.001
AAV-Pfkfb3	0.724	Adrb2-/-	0.019

SFig. 15e N. CD31+EMCN+ cells/Ar

Adrb2WT - Adrb2-/-	p value	AAV-Control - AAV-Pfkfb3	p value
AAV-Control	<0.001	Adrb2WT	0.007
AAV-Pfkfb3	<0.001	Adrb2-/-	0.034
